# Volumetric additive manufacturing of pristine silk-based (bio)inks

**DOI:** 10.1038/s41467-023-35807-7

**Published:** 2023-01-13

**Authors:** Maobin Xie, Liming Lian, Xuan Mu, Zeyu Luo, Carlos Ezio Garciamendez-Mijares, Zhenrui Zhang, Arturo López, Jennifer Manríquez, Xiao Kuang, Junqi Wu, Jugal Kishore Sahoo, Federico Zertuche González, Gang Li, Guosheng Tang, Sushila Maharjan, Jie Guo, David L. Kaplan, Yu Shrike Zhang

**Affiliations:** 1 Division of Engineering in Medicine, Department of Medicine, Brigham and Women’s Hospital, Harvard Medical School, Cambridge, MA 02139 USA; 2 The Sixth Affiliated Hospital of Guangzhou Medical University, Qingyuan People’s Hospital; School of Biomedical Engineering, Guangzhou Medical University, Guangzhou, 511436 P.R. China; 3 Department of Biomedical Engineering, Tufts University, Medford, MA 02155 USA

**Keywords:** Tissue engineering, Biomaterials - proteins, Design, synthesis and processing

## Abstract

Volumetric additive manufacturing (VAM) enables fast photopolymerization of three-dimensional constructs by illuminating dynamically evolving light patterns in the entire build volume. However, the lack of bioinks suitable for VAM is a critical limitation. This study reports rapid volumetric (bio)printing of pristine, unmodified silk-based (silk sericin (SS) and silk fibroin (SF)) (bio)inks to form sophisticated shapes and architectures. Of interest, combined with post-fabrication processing, the (bio)printed SS constructs reveal properties including reversible as well as repeated shrinkage and expansion, or shape-memory; whereas the (bio)printed SF constructs exhibit tunable mechanical performances ranging from a few hundred Pa to hundreds of MPa. Both types of silk-based (bio)inks are cytocompatible. This work supplies expanded bioink libraries for VAM and provides a path forward for rapid volumetric manufacturing of silk constructs, towards broadened biomedical applications.

## Abstract (web summary)

Volumetric additive manufacturing of protein scaffolds has a wide range of possible biomedical applications. Here the authors report on the bioprinting of unmodified silk sericin and silk fibroin inks with shape-memory and tuneable mechanical properties and demonstrate the potential of the inks in different applications.

## Introduction

**Fig. 1:**
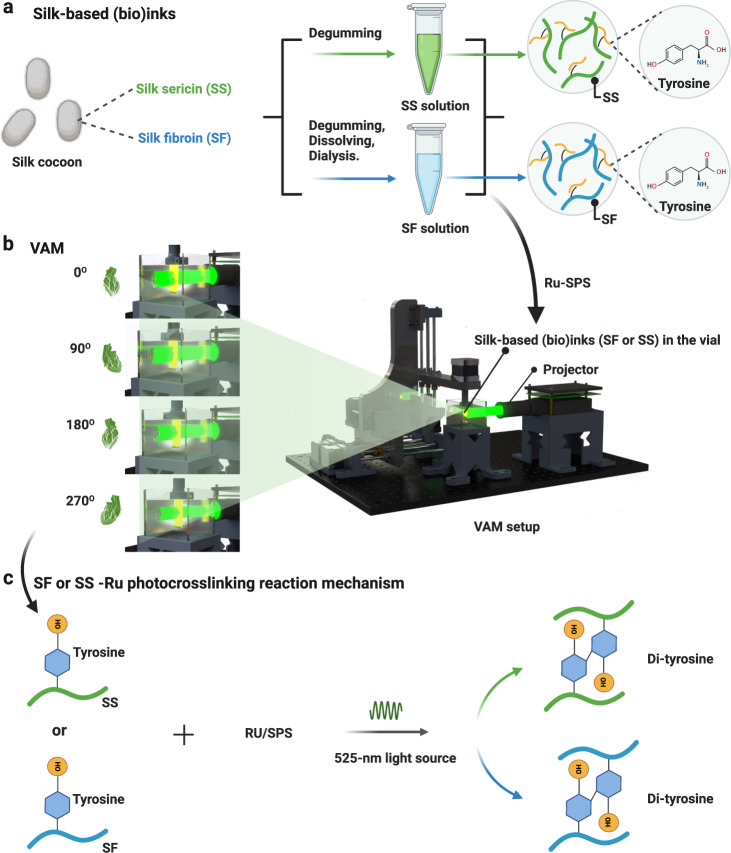
Silk-based (bio)inks (SF and SS) utilized towards VAM. **a** Silk-based (SS and SF) (bio)ink preparation. **b** VAM setup, which mainly includes a projector, a rotation platform, and a transparent (bio)ink-containing vial, as well as a typical (bio)printing process. **c** Photocrosslinking mechanism of silk-based materials (SS or SF)/Ru-SPS system. SS silk sericin, SF silk fibroin, VAM volumetric additive manufacturing, Ru tris-bipyridyl-ruthenium (II) hexahydrate, SPS sodium persulfate.

Advances in additive manufacturing (AM) have facilitated numerous biomedical applications, from medical devices^1,2^ to bioprinting of tissues and organs^3–6^. To overcome geometric constraints and improve the printing speed of the conventional layer-by-layer vat polymerization-based AM technique, volumetric AM (VAM) has been developed^7^. VAM is based on the irradiation of a specific portion of the volume of a photosensitive resin by illumination with dynamically evolving light patterns (Fig. 1). A transparent container of the resin is rotated while being irradiated with computed patterns of light, which are perpendicular to the axis of rotation. The light patterns are emitted from a projector module, and displayed in synchronization with the rotation speed of the vial. The projecting patterns, from the different rotational angles, are computed by a Radon transform; a process similar to that of a computed tomography (CT) scans but applied in reverse^8^. A three-dimensional (3D) distribution of the accumulated light dose is generated after the vial is illuminated from every angle by the light patterns, which results in resin photocrosslinking^9^, leading to the solidification of the desired object. Though VAM has the potential to print sophisticated shapes and architectures at a higher throughput than line-by-line or layer-by-layer AM, the very limited options for (bio)inks to date, mostly due to the early-stage development of the technology, is an issue in fostering future applications using VAM.

Of specific interest here is silkworm silk, as a unique natural protein mainly contains silk sericin (SS) and silk fibroin (SF). SS is a family of adhesive silk proteins, which acts as a protein glue to fix fibroin fibers together in a cocoon^10^. Normally, SS is discarded as a waste after extracting from the silk material. Interestingly, several studies have shown that SS possesses many unique characteristics such as good hydrophilicity, anti-inflammation, anti-oxidation, and anti-bacteria, implying a promising potential of SS in the biomedical area^11,12^. Further, SS is a good additive in bioinks for 3D extrusion bioprinting, for example, when combined with gelatin methacryloyl (GelMA) for applications in wound healing^13^. Nonetheless, bioprinting of pristine, pure SS (bio)inks has not been explored so far in any (bio)printing modalities, let alone VAM.

On the other hand, the clinical translational potential and the wide availability of silkworm silk has resulted in the expansion in the development of SF-based biomaterials^14,15^. In particular, the unique structure, versatility in aqueous processing, biocompatibility, ease of sterilization, thermal stability, controllable degradability and tunable mechanical properties, and water-solubility, as well as the United States Food and Drug Administration (FDA)-approved use of SF in certain medical devices, make SF a promising biomaterial^16,17^.

Recently, SF has elicited considerable interest in 3D bioprinting and tissue biofabrication applications^18,19^. SF is a favorable additive in bioinks for extrusion 3D bioprinting, which has shown potential in crosslinker-free (structural stability due to physical forces, thus no need for chemical crosslinking) gelation and freeform bioprinting^20,21^. Moreover, it is reported that SF can be used for digital light processing (DLP)-based 3D bioprinting through methacryloyl-substitution^22,23^. Of significance, the abundant tyrosine groups (~5%) in SF has, in parallel, prompted its use in the unmodified state as a bioink for 3D bioprinting^24^. Indeed, DLP bioprinting of pristine SF bioinks in combination with the visible-light photoinitiator tris-bipyridyl-ruthenium (II) hexahydrate (Ru)/sodium persulfate (SPS) was lately proposed^25^. Nevertheless, most extrusion and DLP bioprinting of SF alone (without adding any other materials or additives) prefers a high-concentration SF protein (normally 10% or higher) within the bioinks^26^, leading to solution-preparation challenges pre-printing, and oftentimes stiff constructs post-printing. In practice, these above methods normally do not support rapid (bio)printing of volumetrically sophisticated SF shapes and architectures at low SF concentrations (<5%).

In this work, we present VAM of natural, unmodified, pure SS and SF (bio)inks. We evaluate the printabilities of the SS and SF (bio)inks at different concentrations as well as various photoinitiator ratios for optimizations. Due to the unique advantage of VAM to decouple printability of SS and SF (bio)inks with their mechanical properties, unlike that required for extrusion or DLP bioprinting, low concentrations of SS and SF (2.5%, in w/v unless otherwise noted) could be used for rapid (bio)printing with the ability to simultaneously achieve sophisticated volumetric structures, otherwise almost impossible with conventional (bio)printing modalities, within tens to a couple hundred of seconds. The shrinkage and expansive properties, mechanical properties, and other physicochemical properties of both SS and SF prints are characterized; and the cytocompatibilities of SS and SF bioinks are evaluated. Lastly, proof-of-concept biomedical applicational examples of the VAM-(bio)printed SS and SF constructs are demonstrated.

## Results

### Printabilities of SS and SF (bio)inks

**Fig. 2:**
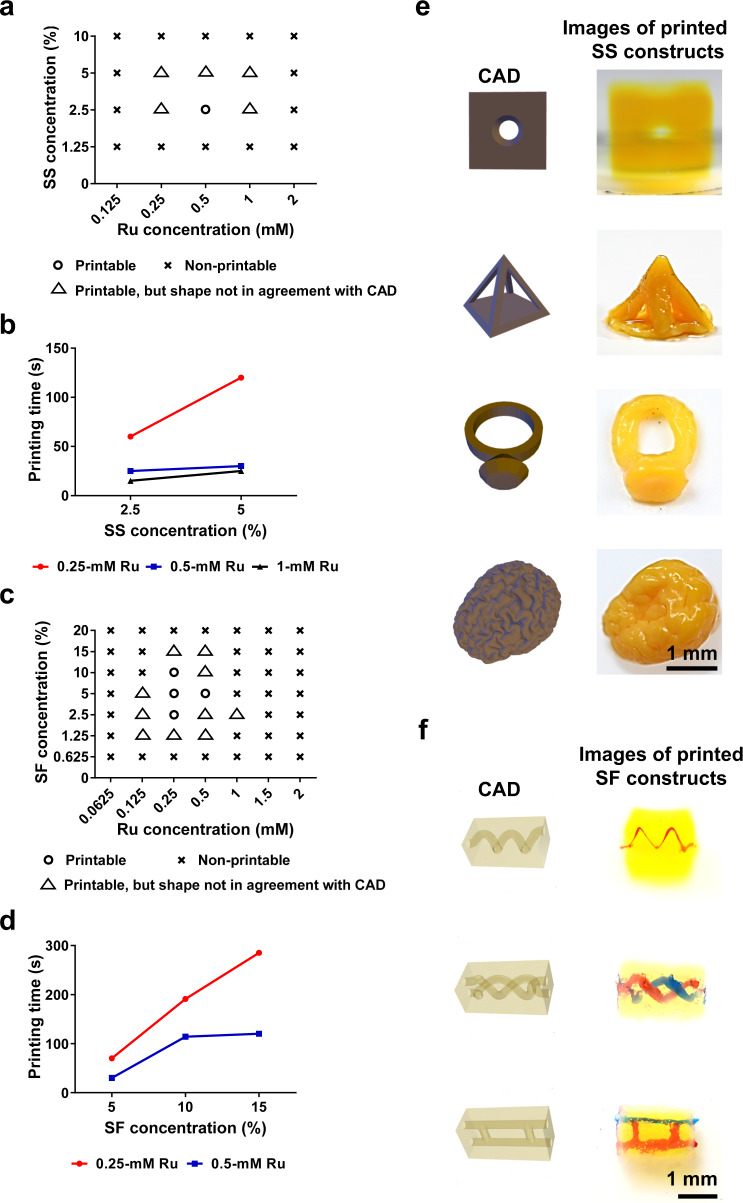
Printing performance of SS and SF (bio)ink formulations. **a** Printability map of SS (bio)ink with different formulations; o- printable, △: printable, but the shape was not agreement with CAD, x: non-printable. **b** Printing times for the same shape (temple of heaven) of SS with different formulations. **c** Printability map of SF (bio)ink with different formulations; o- printable, △: printable, but the shape was not agreement with CAD, x: non-printable. **d** Printing times for a same shape (screw) of SF with different formulations. **e** CAD and photographs of VAM-printed SS objects with printing parameters: 2.5% SS with 0.5-mM Ru/5-mM SPS, light intensity: 3 mW cm^−2^. A channel in cube construct (80 s of printing time; Jaccard similarity index is 84%), hollow triangle construct (60 s of printing time; Jaccard similarity index is 79%), diamond ring construct (80 s of printing time; Jaccard similarity index is 64%), a brain-like construct (65 s of printing time; Jaccard similarity index is 84%). **f** CAD and photographs of VAM-printed SF objects under 3 mW cm^−2^ of light intensity. Construct with a single-spiral channel: 10% SF with 0.25-mM Ru, 165 s of printing time, Jaccard similarity index is 69%; construct with dual spiral channel construct: 5% SF with 0.25-mM Ru/2.5-mM SPS, 57 s of printing time, Jaccard similarity index is 70%; construct with H-shaped channel: 2.5% SF with 0.25-mM Ru/2.5-mM SPS, 117 s of printing time, Jaccard similarity index is 68%. SS: silk sericin. SF: silk fibroin. Ru: ruthenium (II) hexahydrate. CAD: computer-aided design.

VAM of pristine, pure SS and SF (bio)inks in combination with the visible-light photoinitiator Ru/SPS was performed (Fig. 1) on our setup^7^, where the wavelength of the projector light to achieve photocrosslinking was 525 nm. For SS (bio)inks, below 2.5%, there was insufficient material for crosslinking (Fig. 2a); while above 5% SS, crosslinking failed likely due to an insufficient Ru/SPS concentration for crosslinking and higher absorbance at 525 nm than 2.5% SS. Further, the crosslinking times of 2.5% SS were shorter than 5% SS at the same Ru concentrations (Fig. 2b), which might be due to its lower absorption at 525 nm than the 5% and 10% silk, the former of which could accumulate the required light dose in a shorter time during VAM. In summary, the printable range of SS (bio)inks was determined at 2.5-5%.

Specifically, 0.53 was a ratio of width-to-height of the designed computer-aided design (CAD) model of the temple of heaven (Supplementary Fig. 1a). The experimental results from the prints with parameters of 2.5% SS with 0.5-mM Ru and 5-mM SPS (Ru/SPS ratio fixed unless otherwise noted) showed the closest value to the digital design than the other parameters (Supplementary Fig. 1b and c), indicating that these were the optimal conditions for VAM of SS (bio)inks. However, high Ru/SPS concentrations (>0.5-mM Ru/5-mM SPS) resulted in crosslinking only occurring close to the surfaces of the vials, which was also due to the reduced penetration depth of light in the presence of the higher concentrations of Ru/SPS (Supplementary Fig. 2a and b).

In contrast, the SF (bio)inks had a wider printable range of concentrations from 1.25% to 15% (Fig. 2c). For SF (bio)inks, 2.5% to 10% SF showed better printing shape fidelity (i.e., agreement with CAD design (screw)) than higher (>10%) or lower (<2.5%) SF concentrations (with printing parameters of 3 mW cm^−2^ of light intensity) (Supplementary Fig. 3). Below 1.25%, there was insufficient material for crosslinking; while above 15%, crosslinking failed likely due to increased absorbance of SF at the wavelength used (Supplementary Fig. 4) and thereby decreased penetration depth of light. Furthermore, 2.5% to 10% SF combined with 0.25-mM Ru and 2.5-mM SPS could be volumetrically printed with good shape fidelity (i.e., the small values of thread thicknesses of the printed screws; Fig. 2c and Supplementary Fig. 5). Noticeably, 0.5-mM Ru and 5-mM SPS resulted in poorer printing fidelities (i.e., the large values of thread thickness of the printed screw indicating poorer printing fidelities) than 0.25-mM Ru and 2.5-mM SPS for SF (bio)inks at the same corresponding concentrations (Supplementary Fig. 5). This result might also be attributed to the reduced penetration depths of light in the presence of the higher concentrations of Ru/SPS (Supplementary Table 1). Similar to SS, the crosslinking time of 5% SF was shorter than those for 10% SF and 15% SF at the same corresponding Ru/SPS concentrations (Fig. 2d).

Using these inks and under 3 mW cm^−2^ of light illumination, a variety of complex morphologies were printed. For the SS bioinks, a channel-in-cube construct (Supplementary Movie 1; 2.5% SS with 0.5-mM Ru/5-mM SPS, 80 s of printing time), a hollow triangle construct (Supplementary Movie 2; 2.5% SS with 0.5-mM Ru/5-mM SPS, 60 s of printing time), a diamond ring construct (Supplementary Movie 3; 2.5% SS with 0.5-mM Ru/5-mM SPS, 80 s of printing time), and a C60 structure (Supplementary Movie 4; 2.5% SS, 0.5-mM Ru/5-mM SPS, 57 s of printing time) were volumetrically printed (Fig. 2e and Supplementary Fig. 6). Moreover, sophisticated human organ-like structures such as a brain (Fig. 2e and Supplementary Movie 5) and an ear (Supplementary Fig. 6 and Supplementary Movie 6), were readily produced with 2.5% SS combined with 0.5-mM Ru/5-mM SPS, within ~65 s and ~57 s, respectively.

Similarly, for the SF bioinks, sophisticated hollow patterns including a single-spiral channel (10% SF with 0.25-mM Ru/2.5-mM SPS, 165 s of printing time), dual-spiral channels (5% SF with 0.25-mM Ru/2.5-mM SPS, 57 s of printing time), and H-shaped channels (2.5% SF with 0.25-mM Ru/2.5-mM SPS, 117 s of printing time) with diameters from 200 μm to 500 μm embedded in cubes, were volumetrically manufactured (Fig. 2f). Additionally, a lobster shape (Supplementary Movie 7; 2.5% SF, 0.25-mM Ru/2.5-mM SPS, 30 s of printing time), a nut construct (10% SF, 0.25-mM Ru/2.5-mM SPS, 228 s of printing time), and a tooth-like construct (5% SF, 0.25-mM Ru/2.5-mM SPS, 168 s of printing time) were volumetrically printed (Supplementary Fig. 7). Also, a kidney-like construct (5% SF with 0.25-mM Ru/2.5-mM SPS, 57 s of printing time) and a heart-like construct (Supplementary Movie 8; 10% SF with 0.25-mM Ru/2.5-mM SPS, 90 s of printing time) were achievable.

Overall, the volumetric manufacturing ability of these complex shapes and architectures of broad size, pattern, and concentration ranges from both pristine, pure SS and SF bioinks, within tens to a couple hundred of seconds, will likely expand their biomedical utilities.

### Resolutions of volumetric additive manufacturing using SS and SF (bio)inks

**Fig. 3:**
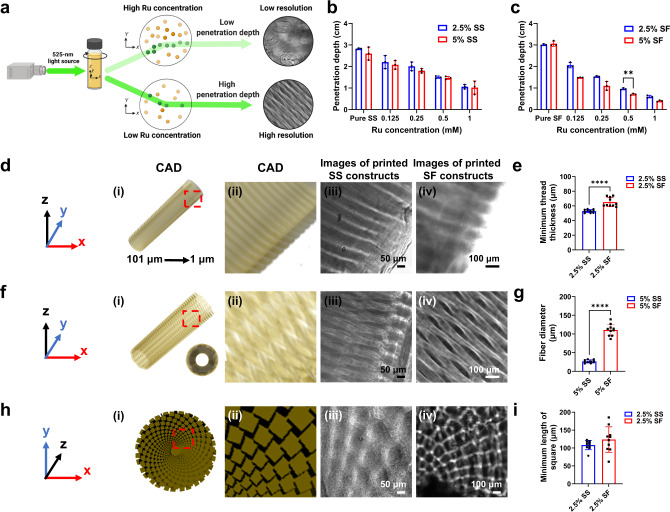
Resolutions with VAM printing of SS and SF (bio)ink formulations. **a** Illustration showing the effect of Ru concentration on printing resolution. **b**, **c** Penetration depths of SS and SF with different formulations. (**d(i)** and **(ii)**) CAD image of the solid bar; thicknesses of the threads from 1 μm to 101 μm. (**d(iii)** and **(iv)**) Microscopic images of the printed solid bar of 2.5% SS and 2.5% SF. The printing parameters were 0.5-mM Ru/5-mM SPS for SS, 0.25-mM Ru/2.5-mM SPS for SF, light intensity: 3 mW cm^−2^. (**e**) Minimum thread thickness of volumetrically printed 2.5% SS and 2.5% SF solid bars. (**f(i)** and **(ii)**) CAD image of the hollow mesh tube. (**f(iii)** and **(iv)**) Microscopic images of the printed hollow mesh tube of 5% SS and 5% SF. The printing parameters were 0.5-mM Ru/5-mM SPS for SS, 0.25-mM Ru/2.5-mM SPS for SF, light intensity: 3 mW cm^−2^. (**g**) Fiber diameter of volumetrically printed 5% SS and 5% SF hollow mesh tubes. (**h(i)** and **(ii)**) CAD image of the radially arranged array of cubes. (**h(iii)** and **(iv)**) Microscopic images of the printed arrays of cubes of (**iii**) 2.5% SS and (**iv**) 2.5% SF. The printing parameters were 0.5-mM Ru/5-mM SPS for SS, 0.25-mM Ru/2.5-mM SPS for SF, light intensity: 3 mW cm^−2^. (**i**) Lengths (x-y plane) of minimum squares of volumetrically printed 2.5% SS and 2.5% SF arrays of cubes. SS: silk sericin. SF: silk fibroin. Ru: ruthenium (II) hexahydrate. CAD: computer-aided design. (**c**) Statistical significances are expressed as **p = 0.00591. Two-way ANOVA. Data are presented as mean values ± SDs. *n* = 3 independent experiments. (**e**), (**g**) and (**i**) Statistical significances are expressed as ****p < 0.0001. Unpaired t test. Data are presented as mean values ± SDs. *n* = 10 indepe*n*dent experiments.

Penetration depth of the projected light is deemed a key factor that affects printing resolution (Fig. 3a)^7^, which was also confirmed in our pre-experiments. Besides, the Ru/SPS concentrations would significantly influence the penetration depth in both SS and SF (bio)ink formulations according to the Beer-Lambert law (Fig. 3b and c).

The printing resolutions of the SS and SF (bio)inks were explored by manufacturing different structures with quantifiable features (Fig. 3d, f, and h). Specifically, a solid rod with bars featuring a set of threads of different thicknesses (from 1 μm to 101 μm) was designed to quantify minimum feature sizes achievable in the various scenarios (Fig. 3d(i)-(iii)). The thickness of the printed 2.5% and 5% SS threads increased with printing time (Supplementary Fig. 8). Of note, the combination of 5% SS, 0.5-mM Ru/5-mM SPS, 3 mW cm^−2^ of light intensity, and 30 s of printing time revealed the smallest thickness of the printed threads (45.9 μm) (Supplementary Fig. 8e). The results indicated that, under a fixed light intensity, the effect of printing time on printing resolution depended on SS concentration; typically, after formation of the object, a prolonged printing time would decrease the printing resolution due to over-crosslinking on the surface of the object.

For SF (bio)inks, the printing resolutions were systematically analyzed by performing a full-factorial design of three factors (SF concentration, SF molecular weight (M_w_), and Ru concentration) each at 2 levels (Supplementary Table 2). The purpose of the full-factorial design was to find out the most impactful factor in determining the printing resolution among these factors and explore the interactions among them. Similarly, a solid rod with bars featuring a set of threads of different thicknesses (from 1 μm to 101 μm) was printed (Fig. 3d(vi) and (v)). For single-factor analyses, the results indicated that Ru/SPS concentration was a more important factor in determining the printing resolution than M_w_ or concentration of SF (Supplementary Fig. 9a and b). Specifically, high Ru/SPS concentrations and M_w_ of SF led to lower printing resolutions, while high SF concentrations resulted in higher printing resolutions (Supplementary Fig. 9c). This decreased printing resolution was again, likely due to the reduced penetration depth for the projected light (Fig. 3c and Supplementary Table 1). Thus, the optimized (bio)ink parameters (Ru/SPS concentration at 0.25 mM/2.5 mM, M_w_ of SF at 88.9 kDa (Supplementary Fig. 10), and SF concentration at 10%) from the full-factorial design, generated the smallest feature size of ~57 μm. Furthermore, there was significant impact of interactional effects between SF concentration and Ru/SPS concentration on the printing resolution (Supplementary Fig. 9d); at a constant Ru/SPS concentration at 0.5 mM/5 mM, 10% SF demonstrated significantly improved printing resolution compared to 2.5% SF, which implied that the SF-Ru/SPS ratio might also be a critical factor in improving the printing resolution.

The effect of different SF concentrations in relation to printing times on printing resolution was subsequently assessed, used in combination with 0.25-mM Ru and 2.5-mM SPS. The thickness of the printed 2.5% silk threads increased with printing time (Supplementary Fig. 9j-n). For 10% SF, the thread was formed when the printing time was above 65 s (Supplementary Fig. 9j(vii), k(vii), n). In contrast, 5% SF improved the printing resolution better than 2.5% SF when utilizing the same printing time (Supplementary Fig. 11).

Beyond solid structures, tubular meshes were printed as well. The mean fiber diameter of 5% SS tubular meshes was 26 μm, with the conditions of 0.5-mM Ru/5-mM SPS, 3 mW cm^−2^ of light intensity, printing time: 90 s; while the mean fiber diameter of 5% SF tubular meshes was 110 μm, with the conditions of 0.25-mM Ru/2.5-mM SPS, 3 mW cm^−2^ of light intensity, 50 s of printing time (Fig. 3f, g). The differences in the resolution of SS and SF might be owing to the different penetration depths (Fig. 3b and c). It was observed as well that the fiber diameter increased with printing time (Supplementary Fig. 12 and Fig. 13).

Additional to the axial direction, the resolutions in the x-y plane were evaluated by printing an array of cubes with different diameters. The minimal feature size (x-y plane) was 108 μm for SS (2.5% SS with 0.5-mM Ru/5-mM SPS, 3 mW cm^−2^ of light intensity, and 57 s of printing time), and 124 μm for SF (2.5% SF with 0.25-mM Ru/2.5-Mm SPS, 3 mW cm^−2^ of light intensity, and 57 s of printing time) (Fig. 3h and i). These results indicated that the different structures might have an impact on the printing resolutions of silk-based (bio)inks. Interestingly, for all the printed structures in Fig. 3, SS showed higher printing resolutions than SF (bio)ink formulations at the same concentrations under their respective optimized conditions (Fig. 3e, g, and i).

### Reversible and repeated shrinkage and expansive properties of VAM-printed SS constructs

Interestingly, the volumetrically printed SS structure, for example, a hollow square (Supplementary Movie 9; 2.5% SS with 0.5-mM Ru/5-mM SPS, 3 mW cm^−2^ of light intensity, printing time: 57 s), shrunk by immersion in >80% ethanol solutions for 2 h (Supplementary Fig. 14). In contrast, the dimensions did not reduce with immersion in <80% ethanol solutions for up to 24 h. Specifically, SS structures, including a hollow square and a C60 structure were volumetrically printed (2.5% SS with 0.5-mM Ru/5-mM SPS, 3 mW cm^−2^ of light intensity, printing time: 57 s) to study their shrinkage and expansive properties (Supplementary Fig. 15 a and b and Supplementary Fig. 16a-g). With post-printing ethanol (100%)-treatment, the diameter and pore size of the printed C60 structure shrunk gradually with increased time (Supplementary Fig. 16b-d). Following that, the same shrunken C60 structure was transferred into the water to induce a dimensional re-expansion (Supplementary Fig. 16e-g), where the diameter and pore size of the shrunken C60 structure readily recovered within 30 min, and then continued to swell to a larger size after 24 h of immersion in water, almost 2 times than the as-printed one. Of note, the change in transparency of the C60 structure during the shrinking and expanding process was apparent. During shrinking, the structure became opaque; and during re-expansion, it restored its transparency again. Moreover, the shrinkage and expansive function of the SS prints (e.g., a rook, 2.5% SS with 0.5-mM Ru/5-mM SPS, 3 mW cm^−2^ of light intensity, printing time: 57 s) were reversible and might be repeated (Supplementary Fig. 17).

Beyond shrinkage, a shape-memory function of the SS constructs was demonstrated (Supplementary Fig. 18a-d). The volumetrically printed “channel-in-cube” structure (2.5% SS with 0.5-mM Ru/5-mM SPS, 3 mW cm^−2^ of light intensity, printing time: 80 s) was shrunken first (induced by immersion in 100% ethanol for 2 h), and then pressed to a deformed shape. Without immersion in water, the deformed shape could not recover (Supplementary Fig. 18a and b); in comparison, when submerged in water, the deformed shape showed complete recovery within 1 h (Supplementary Fig. 18c and d), indicating the swelling mechanism played a key role in shape-recovery of constructs printed from the SS (bio)inks. However, the as-printed SS structures were easily broken without undergoing a shrinking process first (Supplementary Fig. 19), suggesting that a shrinking process would enhance their mechanical performances due to densified polymer chains.

Of note, unlike SS prints, the volumetrically printed SF constructs did not show an obvious re-expansion process (immersion in water for up to 2 h) after shrinking. The SF prints shrunk in size post-treatment by 70% ethanol for 24 h followed by 72 h of air-drying, due to the loss of water content. The diameters, heights, and volumes of printed SF cylinders before (i.e., before air-drying) and after (i.e., after air-drying) shrinking are shown in Supplementary Fig. 20. The lower SF concentrations resulted in greater water losses, leading to increased shrinkage. Specifically, the shrinking ratio of the 2.5% SF prints was ~93%, while that of the 10% SF prints was ~87%.

### Mechanical properties of VAM-printed SS constructs

The compressive and tensile moduli of the SS objects at different SS concentrations and different status (as-printed, shrunken (induced by immersion in 100% ethanol for 2 h), re-expanded (induced by immersion in 100% ethanol for 2 h first, and then immersion in water for 2 h) were compared (Supplementary Fig. 21a-c and Supplementary Fig. 22a-c). For the various SS structures, 2.5% SS resulted in higher compressive moduli than the 5% SS at the same corresponding Ru/SPS concentrations (Supplementary Fig. 21a-c). Meanwhile, the shrunken SS structures in the wet state possessed a much higher compressive modulus (~1000 times) than both as-printed and re-expanded ones in the wet state.

For tensile elastic moduli, the printed 2.5% SS objects resulted in higher tensile elastic moduli than the printed 5% SS objects (Supplementary Fig. 22a); while for both shrunken and re-expanded objects, 5% SS in the wet state showed improved tensile elastic moduli than 2.5% SS in the wet state (Supplementary Fig. 22b and c). Similarly, the shrunken SS structures (induced by immersion in 100% ethanol for 2 h) in the wet state possessed a much higher tensile elastic modulus (~1000 times) than both as-printed and re-expanded ones in the wet state. Of note, after re-expansion from shrinkage, the SS objects could recover both their compressive and tensile moduli to the values of as-printed state (Supplementary Fig. 21c and Supplementary Fig. 22c).

**Fig. 4:**
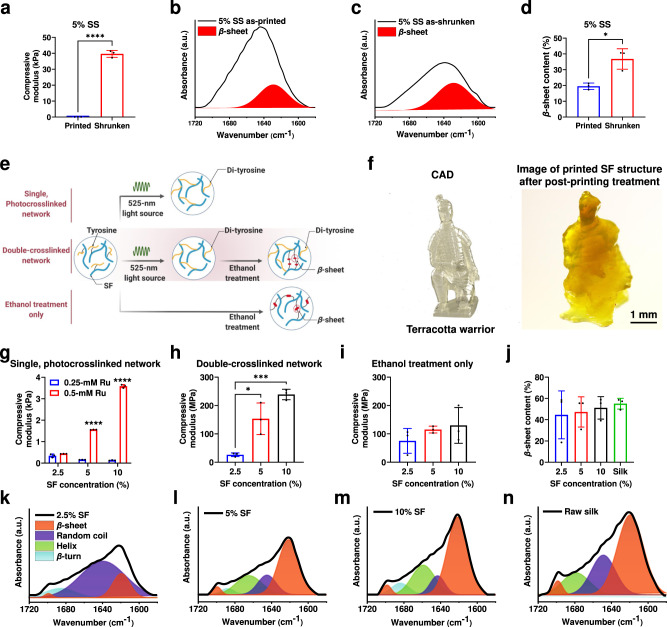
Tunable mechanical strengths and secondary structures of volumetrically printed silk objects. The printing parameters for following SS objects tests were 0.5-mM Ru/5-mM SPS and 3 mW cm^−2^ of light intensity. **a** Compressive moduli of volumetrically printed 5% SS structures in the as-printed and shrunken states. **b–d** FTIR spectra and *β*-sheet content quantification of the 5% SS objects in the as-printed and shrunken states. The printing parameters for following SF objects tests were 0.25-mM or 0.5-mM Ru/2.5-mM or 5-mM SPS and 3 mW cm^−2^ of light intensity. **e** Proposed mechanism of SF constructs with different crosslinking configurations. **f** CAD and photograph of volumetrically printed SF terracotta warrior (2.5% SF, 0.5-mM Ru/5-mM SPS, 3 mW cm^−2^ of light intensity, printing time: 114 s). **g–i** Compressive moduli of volumetrically printed SF objects with different SF concentrations and crosslinking configurations. For both (**h**) double-crosslinked network-group and (**i**) ethanol treatment only-group, the SF objects were treated with 70% ethanol for 24 h followed by 72 h of air-drying. (**j–n**) *β*-sheet content quantification and FTIR spectra of raw silk and volumetrically printed SF objects with a double-crosslinked network (induced by 24 h-treatment of 70% ethanol for 24 h followed by 72 h of air-drying) at different SF concentrations. SS: silk sericin. SF: silk fibroin. CAD: computer-aided design. **a, d** Statistical significances are expressed as *two-tailed *p* < 0.0117, ****two-tailed *p* < 0.0001, compared to the printed group. Unpaired t test. Data are presented as mean values ± SDs. *n* = 3 independent experiments. **g** Statistical significances are expressed as *adjusted *p* < 0.0117, ****adjusted *p* < 0.0001, compared to the 2.5% SF group. Two-way ANOVA. Data are presented as mean values ± SDs. *n* = 3 independent experiments. **h** Statistical significances are expressed as *adjusted *p* = 0.0115, ***adjusted *p* = 0.0008, compared to the 2.5% SF group. One-way ANOVA. Data are presented as mean values ± SDs. *n* = 3 independent experiments.

Fourier-transform infrared spectroscopy (FTIR) analyses provided further insight into the secondary structure of the silk constructs obtained in the as-printed and shrunken states (Fig. 4b-d). For printed SS constructs with post-treatment, the *β*-sheet contents were higher than those in the as-printed states at the same SS concentrations.

### Tunable mechanical properties of VAM-printed SF constructs through post-printing treatment

On the other hand, SF structures such a terracotta warrior (Supplementary Movie 10; 0.25-mM Ru/2.5-mM SPS, 3 mW cm^−2^ of light intensity, printing time: 114 s) were volumetrically printed at a low SF concentration (2.5%), and then processed post-printing with ethanol (70%) for 24 h to induce the formation of a double-crosslinked network (di-tyrosine combined with *β*-sheet conformation), to modulate mechanical properties (Fig. 4e and f).

The compressive moduli of the objects at different SF concentrations and different crosslinking modes were compared (Fig. 4g-i). For SF prints with a single photocrosslinked network, 0.5-mM Ru/5-mM SPS resulted in higher compressive moduli than the 0.25-mM Ru/2.5-mM SPS at the same corresponding SF concentrations (Fig. 4g). Meanwhile, 3.8 mW cm^−2^ of light intensity used during printing improved compressive moduli for the constructs compared to those produced with 3 mW cm^−2^ of light intensity (Supplementary Fig. 23). However, the compressive moduli of the constructs decreased with higher SF concentrations, when 0.25-mM Ru/2.5-mM SPS was used; even longer printing times did not significantly enhance the mechanical strengths of the 2.5-10% silk constructs. The compressive moduli of the 2.5% silk constructs were still higher than those of the 5% and 10% constructs with increased printing times (Supplementary Fig. 24).

With the same printing parameters of 0.25-mM Ru/2.5-mM SPS and 3 mW cm^−2^ of light intensity, the compressive moduli of the 10% SF prints with double-crosslinked networks in the dry state (induced by immersion in 70% ethanol for 24 h followed by 72 h of air-drying) increased to approximately 250 MPa, nearly 8000 times higher than when the 10% SF prints with single photocrosslinked networks were in the wet state, and nearly 2 times higher than the 10% SF objects with *β*-sheets in dry state (i.e., treated with 70% ethanol but without photocrosslinking, followed by 72 h of air-drying) (Fig. 4g-i). This observation was mainly due to the combination of the photocrosslinked di-tyrosine network with small-sized, uniformly distributed *β*-sheet domains (physical crosslinks) (Fig. 4e)^27^. Moreover, the compressive moduli, elongations at break, and compressive stresses at break of SF prints with double-crosslinked networks all increased with increased SF concentration from 2.5% to 10% (Fig. 4h and Supplementary Fig. 25). However, for objects with only the physically crosslinked *β*-sheet network, the elongations at break and compressive stresses at break decreased with higher SF concentrations (Supplementary Fig. 26). This contradictory trend between the double-crosslinked network and physically crosslinked network is likely due to the growth of the *β*-sheet domains being restricted by the pre-existing di-tyrosine crosslinking sites within the double-crosslinked network, and therefore the improved uniformity in the distribution of the small-sized *β*-sheet domains, which improved both elasticity and strength (Fig. 4e)^27^.

FTIR analyses provide additional insights into the secondary structure of the silk materials obtained with the different SF concentrations and network configurations (0.25-mM Ru/2.5-mM SPS and 3 mW cm^−2^ of light intensity; this combination of parameters was used throughout the rest of studies unless otherwise specified). The *β*-sheet content in the amide I region was compared as shown in Fig. 4j-n, Supplementary Figs. 27 and 28. For printed SF constructs with post-treatment, the *β*-sheet contents were higher than those featuring single photocrosslinked networks at the same SF concentrations; moreover, the *β*-sheet contents of the SF objects with double-crosslinked networks increased with SF concentration from 2.5% to 10% (Fig. 4j).

### Additional characterizations of VAM-printed SS constructs

**Fig. 5:**
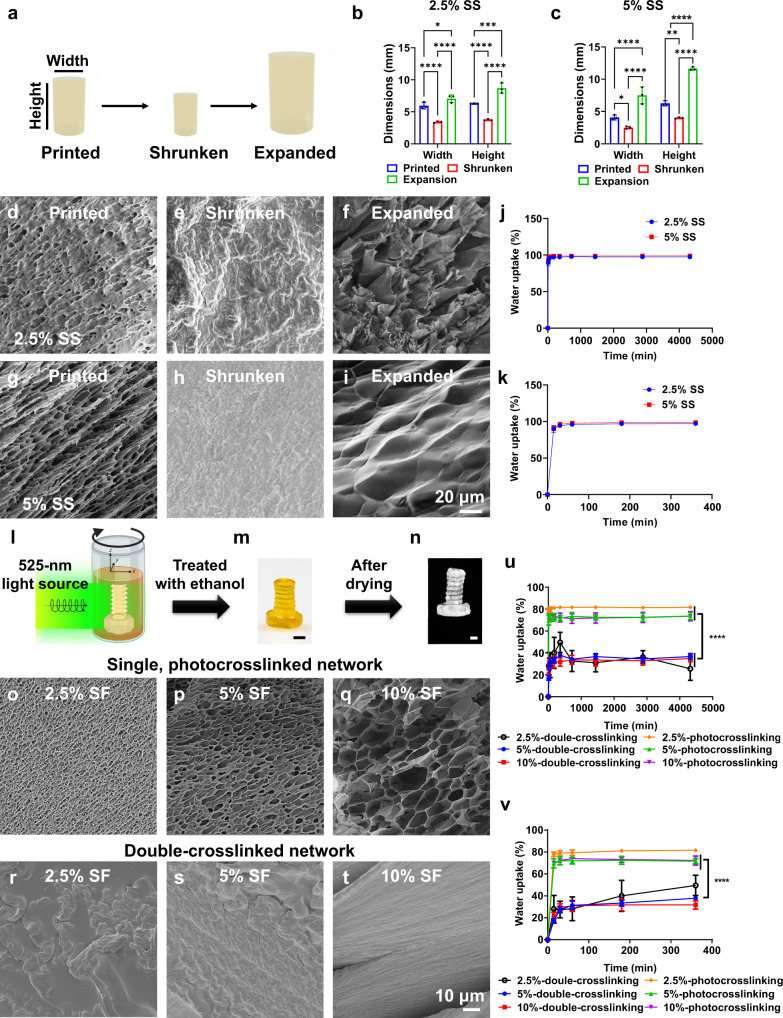
Additional physical properties of volumetrically printed SS and SF objects. The printing parameters for following SS objects tests were 0.5-mM Ru/5-mM SPS and 3 mW cm^−2^ of light intensity. **a** Illustration of the dimensional change property of the volumetrically printed SS cylinder after shrinkage and re-expansion. (**b,** **c**) Width and height change profiles of the volumetrically printed (**b**) 2.5% SS cylinders and (**c**) 5% SS after shrinkage and re-expansion. **d-i** SEM images of cross-sections of the volumetrically printed silk structures at different SS concentrations in the as-printed, shrunken, and re-expanded states. **j,** **k** Water-uptake profiles of the volumetrically printed SS cylinders in the as-printed state after freeze-drying. (**j**) over 72 h and (**k**) over 6 h. The printing parameters for following SF objects tests were 0.25-mM Ru/2.5-mM SPS and 3 mW cm^−2^ of light intensity. (**l**) Illustration of the VAM process, where a silk screw was printed upon simultaneous photopolymerization of the SF ink in the rotating vial. **m** Photograph of the same screw after 70% ethanol treatment for 24 h. Scale bar: 500 μm. (**n**) Microscopic image of the same screw after evaporating the ethanol solution. Scale bar: 500 μm. (**o-t**) SEM images of cross-sections of the volumetrically printed SF screws with a single, photocrosslinked network or a double-crosslinked network (treated with 70% ethanol for 24 h followed by 72 h of air-drying) at different SF concentrations. (**u** and **v**) Water-uptake profiles of the volumetrically printed SF screws with a single, photocrosslinked network and a double-crosslinked network (treated with 70% ethanol for 24 h followed by 72 h of air-drying) (**u**) over 72 h and (**v**) over 6 h. SS: silk sericin. SF: silk fibroin. **b,** **c** Statistical significances are expressed as *adjusted *p* < 0.0347, ***adjusted *p* = 0.0001, ****adjusted *p* < 0.0001. Two-way ANOVA. Data are presented as mean values ± SDs. *n* = 3 independent experiments. **u,** **v** Statistical significances are expressed as *adjusted *p* = 0.0185, ** adjusted *p* = 0.016, **** adjusted *p* < 0.0001. Two-way ANOVA. Data are presented as mean values ± SDs. In figures (**u and v**) all groups were compared to the 5% double-crosslinked group. (**j**), (**k**), (**u**) and (**v**) *n* = 4 independent experiments.

The printing resolution of volumetrically printed SS structures (e.g., a solid bar; 2.5% SS, 0.5-mM Ru/5-mM SPS, 3 mW cm^−2^ of light intensity, and 30 s of printing time) changed accordingly after the dimensional shrinkage and re-expansion (Supplementary Fig. 29). Specifically, the resolution of volumetrically printed SS structures improved after being shrunken (induced by immersion in 100% ethanol for 2 h), while decreased after expansion (induced by immersion in 100% ethanol for 2 h first, and then immersion in water for 2 h). The dimensional changes and cross-sectional images of volumetrically printed SS cylinders (2.5% and 5% SS with 0.5-mM Ru/5-mM SPS, 3 mW cm^−2^ of light intensity, printing time: 28 s) in the as-printed, shrunken, and re-expanded states are shown in Fig. 5a-i. Both as-printed and re-expanded SS objects showed porous structures. Of note, the re-expanded SS cylinders suggested increased pore sizes and irregular arrays compared to the as-printed ones. However, the shrunken SS objects indicated solid and dense structures after dehydration without any noticeable pores present.

The in vitro water-uptake properties of volumetrically manufactured SS structures (2.5% and 5% SS) before and after shrinkage in the dry state were evaluated (Fig. 5j and k and Supplementary Fig. 30). Those before and after shrinkage exhibited similar water-uptake rates (~100%) in phosphate-buffered saline (PBS) at 37 °C within 3 days for all SS concentrations assessed. Moreover, the expansion in dimension of the SS structures occurred following the water-uptake process (Supplementary Fig. 31), and the 2.5% SS showed a higher dimensional expansion rate than the 5% SS. Further, the SS constructs (2.5% and 5% SS) in the as-printed and shrunken states both degraded in protease XIV solution and *α*-chymotrypsin solution at 37 °C within 12 h (Supplementary Fig. 32).

### Additional characterizations of VAM-printed SF constructs

Similarly, the changes in resolution of printed SF screws (Supplementary Movie 11) before and after shrinking were compared (Supplementary Fig. 33). The resolution of the printed SF screws, with concentrations from 2.5% to 10%, all improved after shrinkage. However, severe shape-deformation was observed in the printed 2.5% silk screws after shrinking (Supplementary Fig. 33a), likely due to excessively rapid, anisotropic water-loss. Figure 5l-n shows schematic and images of a printed silk screw (10% SF) with a single photocrosslinked network before ethanol treatment, and then with a double-crosslinked network after 70% ethanol treatment for 24 h (Fig. 5m and n). As described, the as-printed SF screws were first immersed in water to remove uncrosslinked SF and Ru/SPS residues and then treated with 70% ethanol aqueous solution for 24 h to induce the formation of*β*-sheets, becoming double-crosslinked (Fig. 5m); consequently, the printed silk screws were dehydrated and thus shrunken (Fig. 5n). The VAM-printed SF (2.5-10%) screws with a single photocrosslinked network showed porous structures (Fig. 5o-t). In comparison, volumetrically manufactured SF (2.5-10%) screws with a double-crosslinked network indicated solid and dense structures after dehydration without any noticeable pores present.

Meanwhile, the in vitro water-uptake properties of volumetrically manufactured SF screws (2.5-10% SF) with a single photocrosslinked network or a double-crosslinked network were evaluated as well (Fig. 5u and v). Those with a single photocrosslinked network exhibited significantly faster water-uptake rates (~80%) in PBS at 37 °C within 30 min when compared to their counterparts with a double-crosslinked network (<40%) for all SF concentrations assessed. Specifically, for both the single- and double-crosslinked networks, the 2.5% SF screws showed the highest water-uptake capability compared to the 5% and 10% screws at the initial 6 h. The results indicated that the water-uptake ability of volumetrically manufactured SF prints could be tuned by the crosslinking network configuration as well as SF concentration.

Further, the in vitro degradation profiles of the VAM-printed silk screws (2.5-10% SF) (without cells) with single and double-crosslinked networks were evaluated in PBS, protease XIV solution, and *α*-chymotrypsin solution at 37 °C for up to 6 months (Supplementary Fig. 34). Generally, SF screws with a double-crosslinked network showed significantly better stability and thus slower degradation at all SF concentrations than those containing only a single, photocrosslinked network. Furthermore, for both types of networks, the 10% SF screws suggested the slowest degradation rates in comparison to both the 2.5% and 5% screws. Of note, though both protease XIV and*α*-chymotrypsin can degrade these protein materials effectively, the 10% SF screws featuring a double-crosslinked network notably degraded after 4 months of incubation with protease XIV PBS solution, while retaining stability over 6 months of incubation with the *α*-chymotrypsin PBS solution. These data demonstrated the tunability of the degradation profiles of volumetrically manufactured silk objects.

### Bioprinting and cytocompatibility

**Fig. 6:**
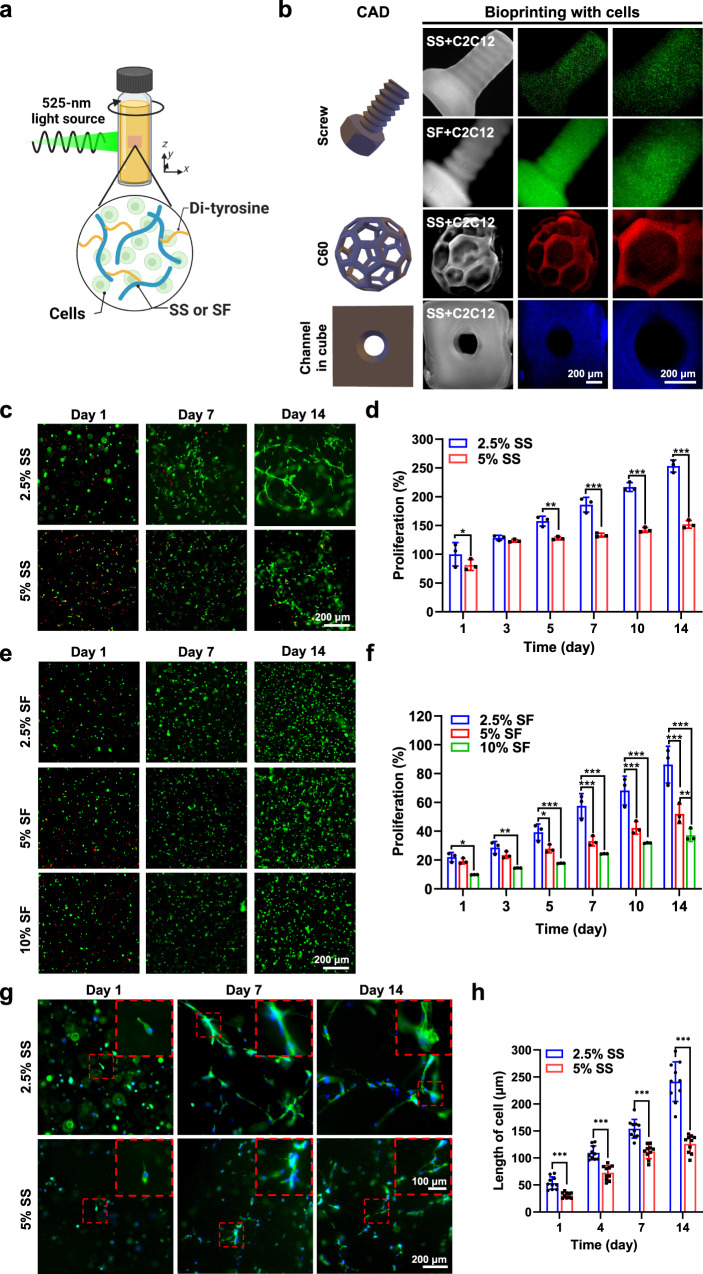
Volumetric bioprinting and cytocompatibility of SS and SF constructs. The printing parameters for the following tests were 0.5-mM Ru/5-mM SPS for SS, 0.25-mM Ru/2.5-mM SPS for SF, 5 × 10^6^ cells mL^−1^, and 3 mW cm^−2^ of light intensity. **a** Illustration and (**b**) microscopic images of the volumetrically bioprinted SS or SF structures with C2C12 cells embedded. A screw structure (45 s of printing time for SS + C2C12, 57 s of printing time for SF + C2C12), a C60 structure (57 s of printing time), and a channel in cube structure (45 s of printing time). (**c**) Live/dead images and (**d**) proliferation profiles of C2C12 cells cultured within the volumetrically bioprinted 2.5% and 5% SS constructs for 14 days. **e** Live/dead images and (**f**) proliferation profiles of C2C12 cells cultured within the volumetrically bioprinted 2.5%, 5% and 10% SF constructs for 14 days. Live cells shown in green and dead in red. (**g**) Microscopic images and (**h**) length of cells of the growth of C2C12 stained for F-actin (green) and nuclei (blue) within the volumetrically bioprinted 2.5% and 5% SS constructs. SS: silk sericin. SF: silk fibroin. CAD: computer-aided design. Statistical significances are expressed as *adjusted *p* < 0.05, **adjusted *p* < 0.01, ***adjusted *p* < 0.001. Two-way ANOVA. Data are presented as mean values ± SDs. (**d**) and (**f**) *n* = 3 independent experiments. (**h**) *n* = 3 independent experiments.

The SS and SF bioinks enabled the encapsulation of myoblasts (C2C12, 5×10^6^ cells mL^−1^) using VAM, for bioprinting and the evaluation of in vitro cytocompatibility (Fig. 6 and Supplementary Figs. 35 and 36). With the parameters of 5 × 10^6^ cells mL^−1^, 0.5-mM Ru/5-mM SPS for SS and 0.25-mM Ru/2.5-mM SPS for SF, and 3 mW cm^−2^ of light intensity, a screw structure (45 s of printing time for 2.5% SS, 57 s of printing time for 2.5% SF), a C60 structure (2.5% SS, 57 s of printing time), and a channel-in-a-cube structure (2.5% SS, 45 s of printing time) were bioprinted, indicating that the embedded cells at the density used did not noticeably affect the printability of the SS and SF bioinks (Fig. 6a and b). Additionally, the embedded cells remained viable and maintained high cell metabolic activities within both printed SS (2.5% and 5%, 5 × 10^6^ cells mL^−1^, 0.5-mM Ru/5-mM SPS, 3 mW cm^−2^ of light intensity, printing time: 28 s) and SF (2.5%, 5%, and 10%, 5×10^6^ cells mL^−1^, 0.25-mM Ru/2.5-mM SPS, 3 mW cm^−2^ of light intensity, printing time: 28 s) hydrogels for up to 14 days evaluated (Fig. 6c-f and Supplementary Figs. 35a and 36a-c). Specifically, the 2.5% SS hydrogels supported higher cell metabolic activities than the 5% SS hydrogels; and the 2.5% SF hydrogels supported higher cell metabolic activities than both the 5% and 10% SF hydrogels for fibroblasts (NIH/3T3), myoblasts (C2C12), and breast cancer cells (MDA-MB-231) (Supplementary Fig. 36).

It is interesting to note that nonetheless, the embedded myoblasts (C2C12) showed spreading performances only within the bioprinted SS structures, cultured for up to 14 days, indicating their good spreading activities within the SS hydrogels, with the 2.5% SS hydrogels supporting better cell spreading than the 5% SS hydrogels (Fig. 6g and h and Supplementary Fig. 35b). On the other hand, spreading of the same cells was found to be minimum within the printed SF structures at all concentrations (2.5-10%) over the course of culture.

### Proof-of-concept medical applications for VAM-printed silk devices

**Fig. 7:**
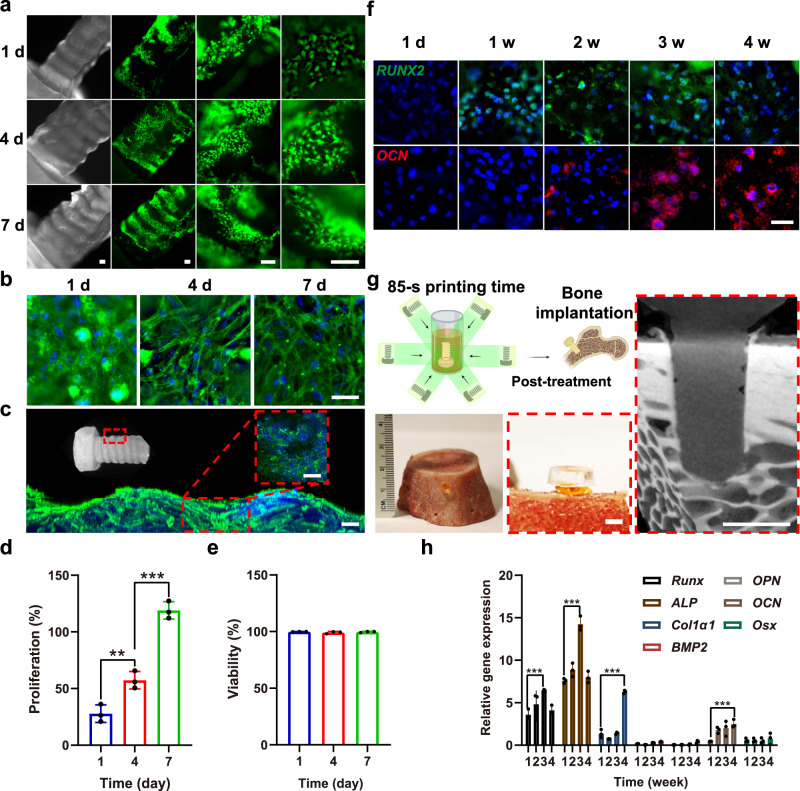
Proof-of-concept medical applications for volumetrically printed SF screws. The printing parameters for the following tests were 0.25-mM Ru/2.5-mM SPS, 5×10^6^ cells mL^−1^, and 3 mW cm^−2^ of light intensity. **a** Live/dead images of MSCs cultured on the surfaces of volumetrically printed 10% SF screws with a double-crosslinked network induced by immersed in 70% ethanol for 24 h followed by 72 h of air-drying. Live cells shown in green and dead in red. Scale bar: 200 μm. **b, c** Microscopic and confocal images showing the growth of MSCs stained for F-actin (green) and nuclei (blue) on the surfaces of volumetrically printed 10% SF screws with a double-crosslinked network induced by immersed in 70% ethanol for 24 h. Scale bar in b: 50 μm; in c: 100 μm; in inset: 200 μm. **d, e** Proliferation and viability profiles of MSCs cultured on the surfaces of volumetrically printed 10% SF screws with a double-crosslinked network induced by immersed in 70% ethanol for 24 h. **f** MSC immunostaining images showing *RUNX2* and *OCN* expressions. Scale bar: 50 μm. **g** Schematic, photograph, and micro-CT scanning image showing ex vivo implantation test of the volumetrically printed 10% SF screws with a double-crosslinked network induced by immersed in 70% ethanol for 24 h followed by 72 h of air-drying in a porcine femur. Scale bar: 1.5 mm. **h** Expression levels of representative genes indicating osteoblast-formation during the 4-week culture period. *ALP*: alkaline phosphatase. *Runx:* runt-related transcription factor. *COL1α1*: collagen type I alpha 1 chain. *OCN*: osteocalcin. *BMP2*: bone morphogenetic protein 2. *Osx*: osterix. *OPN*: osteopontin. **d, e** Statistical significance expressed as **adjusted *p* = 0.008, ***adjusted *p* < 0.001. One-way ANOVA. Data are presented as mean values ± SDs. *n* = 3 indepe*n*dent experiments. (**h**) Statistical significance expressed as ***adjusted *p* = 0.0001. Two-way ANOVA. Data are presented as mean values ± SDs. *n* = 3 indepe*n*dent experiments.

The volumetrically manufactured 10% SF screws (without cells) after postprinting 70% ethanol-treatment for 24 h followed by 72 h of air-drying, were seeded with human mesenchymal stem cells (MSCs) to evaluate cell differentiation (Fig. 7). MSCs sustained proliferation and viability at close to 100% over 7 days of culture (Fig. 7a, d, and e), indicating cytocompatibility of the double-crosslinked SF screws. Furthermore, extensive spreading of MSCs was observed on the surfaces of the double-crosslinked SF screws (Fig. 7b and c).

Interestingly, immunostaining and Alizarin Red S (ARS) staining revealed that the MSCs differentiated into osteoblasts after adhering and proliferating on the surfaces of the 10% SF screws with a double-crosslinked network, when biochemically induced towards osteogenic differentiation for 4 weeks (Fig. 7f and Supplementary Fig. 37). The expression of osteogenesis-related genes (alkaline phosphatase (*ALP*), runt-related transcription factor (*Runx*), collagen type I alpha 1 chain (*COL1α1*), osteocalcin (*OCN*), bone morphogenetic protein 2 (*BMP2*), osterix (*Osx*), and osteopontin (*OPN*)) were consistent with the staining results, suggesting support from the VAM-printed SF screws for osteogenic outcomes (Fig. 7f and h).

The VAM-printed 10% SF screws (0.25-mM Ru/2.5-mM SPS and 3 mW cm^−2^ of light intensity) (without cells) with a double-crosslinked network (after 70% ethanol treatment for 24 h and air drying for 3 days, therefore no shrinkage and defamation would occur after this process) were also implanted in an ex vivo porcine femur model. The screw could be tightened into the cortical bone via a hole drilled slightly smaller in diameter than the screw, without any noticeable breakage or deformation (Fig. 7g), revealing the feasibility of bone-implantation or device-fixation with these VAM-printed silk screws (without cells) containing a double-crosslinked network.

### Chicken chorioallantoic membrane (CAM) assay

The volumetrically bioprinted constructs (i.e., cylinders and cylinders with channels) were implanted on day-7 *ex ovo* CAM and were collected after 7 days of additional incubation^28^ (Supplementary Fig. 38a and b). The extents of angiogenesis were determined using an image-processing method (Supplementary Fig. 38c). The average lengths of newly formed blood vessels (BVs) were measured to quantify the angiogenic responses surrounding the different volumetrically bioprinted constructs. As presented in Supplementary Fig. 38c, the highest average length of BVs was associated with the SS + vascular endothelial growth factor (VEGF) + human umbilical vein endothelial cell (HUVEC) group (Supplementary Fig. 39a). We also quantified the BV densities within the constructs (Supplementary Fig. 39b). BV density was calculated as the number of BVs within 1 mm of the interface along the scaffold/tissue border. Comparable to the other results, the density of the BVs in the SS + VEGF + HUVEC group was significantly higher than those in all the other groups. These observations proved that the volumetric bioprinting procedure did not affect the functionality of HUVECs or VEGF.

## Discussion

In this study, volumetric (bio)printing of silk-based (SS and SF) (bio)inks was demonstrated. Especially, the pristine naturally derived SS, without modifications, was used as the sole bioink component for printing^25^. Based on previous studies^7,25,29^, Ru/SPS reacts with tyrosine groups in the SS and SF materials that facilitate their crosslinking without requiring additional chemical modifications. Thus, Ru/SPS combinations that enable reactions between tyrosine groups under green light were chosen as the photoinitiator in our study. The bioink molecules scatter the projection light, but this effect should be very weak according to our full-factorial analyses. High concentrations of SS, SF, and Ru/SPS would reduce penetration depth due to their high absorption at 525 nm. Under 3 mW cm^−2^ of light intensity, the favorable SS/Ru/SPS and SF/Ru/SPS combinations for VAM were 2.5% SS containing 0.5-mM Ru/5-mM SPS and 5-10% SF containing 0.25-mM Ru/2.5-mM SPS, respectively. The reason why the 2.5% SS required higher Ru (0.5 mM) than the 2.5% SF (0.25 mM) may be due to the antioxidative property of SS contributing to radical-scavenging during Ru-mediated photocrosslinking^30,31^. Further, the difference in M_w_ of SS and SF might have contributed to such differences observed in their biofabrication windows.

The volumetric manufacturing ability of these complex shapes and architectures (including geometrical structures, organ-like structures, and hollow vascular-like structures) from both pristine, pure SS and SF bioinks, indicated the broad application potentials of VAM of both SS and SF bioinks in biomedical areas.

In general, high SF concentrations (>5-10%) are needed in extrusion-based bioprinting to maintain shape fidelities of bioprinted constructs^20,23^. However, high SF concentrations lead to dense polymer networks and decrease the activities of embedded cells after bioprinting^32^. Due to the unique advantage of VAM to decouple printability of the silk-based (bio)ink with concentration and mechanical properties, low concentrations of SS (2.5%) and SF (2.5%) could be used for volumetric (bio)printing with the ability to simultaneously achieve sophisticated spatial architectures.

The printing resolution of the volumetrically printed silk objects mainly depends on photoinitiator concentration and printed structure (Fig. 3)^7,9^. Besides, the volumetric printing resolution can be affected by many other factors, such as the type of resin, crosslinking mechanism, crosslinking density, pixel size of the projector, optics, accuracy of volumetric dose-reconstruction, and print time, among others^33^. However, the intrinsic characteristics of the bioink, such as composition, affect the volumetric printing resolution, a topic that is rarely explored. In this study, we found that Ru concentration was a more important factor in determining the resolution than M_w_ and concentration of SF (Supplementary Fig. 9a and b). Additionally, the interactions between the (bio)ink concentration and printing parameter (printing time) had a significant influence on the printing resolution, which can be possibly considered as a factor for VAM of other photoresins as well.

Further, a shrinkage and re-expansion function of volumetrically printed SS structures was observed. The shrinkage property of volumetrically printed structures was induced by immersion in 100% ethanol solution for a least 2 h; during the shrinking process, the water molecules were replaced by the ethanol molecules, and the dimension, opacity, and mechanical strength of volumetrically printed SS structures would change accordingly (Supplementary Figs. 14-16). The SS constructs formed *β*-sheets when contacted with ethanol solution during the shrinking process. Unlike SF constructs, as they were again immersed in an aqueous medium, water molecules penetrated into the networks of the shrunken SS hydrogels to cause swelling and an increase in size^34^, possibly due to the photo-oxidation of di-tyrosine resulting in some inhibition of large-sized *β*-sheet formation^25^. More importantly, the shrinkage and re-expansion process could be reversed and repeated (Supplementary Fig. 17), suggesting a series of potential biomedical applications such as four-dimensional (4D) printing, soft robotics, shape-memory devices, and drug delivery, among others.

The reason why low-silk-concentration prints (2.5%) in the as-printed state showed higher compressive moduli compared to those with high silk concentrations (5%) in the as-printed state, could be likely attributed to the lower penetration depths of high-concentration silk (bio)inks that decreased their overall crosslinking densities. For SF, further combined with post-printing processing (immersed in 70% ethanol for 24 h followed by 72 h of air-drying), the printed 2.5-10% SF objects (0.25-0.5 mM Ru/2.5-5 mM SPS, and 3 or 3.8 mW cm^−2^ of light intensity; without cells) showed tunable compressive moduli from hundreds of Pa to approximately 250 MPa (Fig. 4g and h), with increased mechanical performance due to the double-crosslinked network that combined photocrosslinked di-tyrosine network with the small-sized and uniformly distributed *β*-sheet domains for physical crosslinks^27^. The compression modulus (250 MPa) of VAM-printed 10% SF screws with a double-crosslinked network (in the dry state) (without cells) was also higher than those of previously reported silk materials (in the dry state) that mainly possessed secondary molecular domains alone (e.g., *α*-coils, *β*-sheets)^35^. The growth of the *β*-sheet domains was potentially restricted by the pre-existing di-tyrosine crosslinking sites within the double-crosslinked network; therefore, the small-sized *β*-sheet domains became uniformly distributed inside the silk constructs as the physical crosslinker, contributing to improved mechanics^27^.

Compared to a previous study on silk screws produced with 30% silk and a centrifugal casting technique (mainly with a *β*-sheet conformation alone) where ~30% of the mass was degraded after 7 weeks in the in vitro enzymatic degradation tests (5 U mL^−1^ of protease XIV PBS solution)^36^, the VAM-printed 10% SF screws with a double-crosslinked network maintained ~100% of their initial masses over 4 months under the same degradation conditions (Supplementary Fig. 34). At the same concentrations of SF, the formation of uniform and small-sized *β*-sheets within the printed constructs beyond the photocrosslinked network reduced in vitro degradation rates in both PBS and the two enzyme (protease XIV and *α*-chymotrypsin) solutions evaluated. Therefore, considering the mechanical, water-uptake, and degradation properties, these newer-generation VAM-printed 10% SF screws featuring a double-crosslinked network were utilized for the subsequent proof-of-concept cell and medical device applications.

Importantly, the cell-laden silk (bio)inks (2.5% and 5% SS, 0.5-mM Ru/5-mM SPS and 3 mW cm^−2^ of light intensity; 2.5-10% SF with a single photocrosslinked network, 0.25-mM Ru/2.5-mM SPS and 3 mW cm^−2^ of light intensity) showed favorable cytocompatibility. Specifically, both 2.5% SS and 2.5% SF constructs supported better proliferation rates and viability than constructs with higher silk concentrations (5% SS, or 5% and 10% SF; Fig. 6). Our results are consistent with previous reports where low SS and SF concentrations are more suitable for the proliferation of embedded cells than them at high concentrations^37–39^. The cytocompatibility of low-concentration (2.5%) SS and SF bioinks made them suitable for VAM applications where living cells are encapsulated. Of note, unlike SF, SS constructs supported desired activities (such as spreading) of encapsulated cells, implying a more promising biomedical potential than SF for cell culture-related applications.

Further, seeded on the surfaces of the VAM-printed SF screws featuring a double-crosslinked network, MSCs retained high proliferation rates for 7 days, verifying the cytocompatibility of the material. Spreading of the MSCs was also observed. The MSCs differentiated into osteoblasts over 4-weeks when subjected to osteogenic medium, suggesting the potential for cellular integration and bone-formation with these volumetrically printed and secondarily crosslinked SF screws. Both *ALP* and *Runx* (known as a precocious transcriptional factor during the osteoblastic differentiation) are involved in the initial phases (normally first 3 weeks) of the osteogenic differentiation, which was perhaps the reason why the expression of these osteogenesis-related genes (i.e., *ALP* and *Runx*) were reduced after 4 weeks of culture^38^. In addition, ex vivo implantation tests indicated bone-fixation potential of the SF screws (Fig. 7g). Moreover, VAM-printed SS constructs containing HUVECs, VEGF, or their combination could maintain their functions when investigated in the CAM assay. There was no significant difference observed in the lengths of BVs between the groups of solid cylinders and cylinders with channels. These *ex ovo* CAM assays shed light on the efficacy of VAM as a functional approach for creating cell-laden implants or tissue models.

In summary, the natural, unmodified SS and SF (bio)inks were successfully applied to fabrication conditions optimized for VAM, both of which showed excellent printability of sophisticated shapes and architectures at concentrations as low as 2.5%. The shrinkage and re-expansion characteristic of the SS prints was reversable, indicating potential applications in shape-memory materials, 4D printing, soft robotics, and drug delivery. The compressive moduli of volumetrically the prepared cell-free SF prints were tunable from a few hundred Pa to approximately 250 MPa, with further post-print processing as an option to introduce the double-crosslinked network beyond photocrosslinking. Of note, both SS and SF (bio)inks exhibited cytocompatibility and favorable cell growth in vitro. Our proof-of-concept studies clearly demonstrated bone-formation potential ex vivo of VAM-printed SF screws. In conclusion, this work provides expanded (bio)ink libraries for VAM, while simultaneously offering additional avenues for rapid volumetric manufacturing of silk-based materials for broadened applications in biomedicine.

## Methods

### Materials and cell lines

Silkworm (*Bombyx mori*) cocoons were purchased from Tajima Shoji Co., Ltd., Japan. Ru and SPS were obtained from Advanced Biomatrix, USA. Human MSCs and osteogenic differentiation medium bullet kit were purchased from Lonza, Switzerland. Bis-Tris- gel (NuPAGE 4-12%) was purchased from Invitrogen, USA. Dimethyl sulfoxide (DMSO), bovine serum albumin (BSA), 4’,6-diamidino-2-phenylindole (DAPI), formalin, Triton X-100, ethanol, protease XIV, *α*-chymotrypsin, Na_2_CO_3_, and lithium bromide (LiBr) were purchased from Sigma-Aldrich, USA. Dulbecco’s PBS (DPBS), Dulbecco’s modified Eagle medium (DMEM), PrestoBlue, 3-(4,5-dimethylthiazol-2-yl)−5-(3-carboxymethoxyphenyl)−2-(4-sulfophenyl)−2H-tetrazolium (MTS), Live/dead viability/cytotoxicity kit, antibiotic-antimycotic (Anti-Anti), fetal bovine serum (FBS), trypsin-ethylenediaminetetraacetic acid (EDTA), Alexa 488-phalloidin, dialysis membranes (M_w_ cutoff = 12,000-14,000 Da), and SYBR™ Green qPCR Master Mix were purchased from Thermo Fisher Scientific, USA. Sterile syringe filters (0.22 μm in pore size) and vacuum filtration systems (0.22 μm in pore size) were purchased from VWR International, USA. Fluorescent color dyes were purchased from CreateX Colors, USA. Mouse anti-RUNX2 antibody (ab76956), mouse anti-osteocalcin antibody (ab198228), and goat anti-mouse IgG H&L (Alexa Fluor 488) (ab150113) were purchased from Abcam, USA. RNeasy mini kit and QuantiTect reverse transcription kit were purchased from Qiagen, Germany. ARS was purchased from Sciencell, USA. Myoblasts (C2C12), fibroblasts (NIH/3T3), breast cancer cells (MDA-MB-231) and HUVEC were purchased from ATCC, USA. Catalog numbers for all commercial reagents are provided in the Supplementary Catalog Number List.

### SS and SF (bio)ink preparations

The SS and SF solutions were prepared according to our previous protocols^40–42^. For SS, *B. mori* cocoons were cut into pieces and boiled in 0.02-M aqueous Na_2_CO_3_ solution for 30 min to remove SS from SF, then the SS solution (mean M_w_ of SS: 59.7 kDa) was dialyzed with distilled water for 3 days (Supplementary Fig. 10). For SF, *B. mori* cocoons were cut into pieces and boiled in 0.02-M aqueous Na_2_CO_3_ solution for 15 min (high M_w_ of SF: 108.7 kDa) or for 30 min (low M_w_ of SF: 88.9 kDa)^32^. Then, the degummed SF was sufficiently rinsed in distilled water for two times, dried overnight in hood, and dissolved in 9.3-M LiBr at a ratio of 1:4 (w/v) at 60 °C for 4 h. After dialyzing the SF/LiBr solution with distilled water for 3 days, the solution was centrifuged twice at 9,000 rpm for 20 min. Next, the precipitate was discarded, and the supernatant was filtered with 0.22-μm filters. The SS and SF solutions were loaded into dialysis bags and placed in the hood for concentrating. The final concentration was calculated by weighting the remaining mass after evaporating the water of the SS or SF solution. The SS and SF (bio)inks were prepared by mixing with the Ru-SPS photoinitiator complex (Ru and SPS were dissolved to stock concentrations separately, and then mixed at a fixed ratio of 1:10 (v/v) before use) at desired ratios in the dark at room temperature.

### VAM procedure

Silk objects were volumetrically printed with a customized volumetric bioprinter setup equipped with an LED light engine (PRO4500, Wintech, USA), with a maximum output power of 1100 mW^35^. This projector had WXGA resolution (912×1140 pixels), and 50-μm pixel size, illuminating the resin in a rotating vial. The Radon transform algorithm, iterative algorithm and filtered back projection algorithm were used in VAM. All projection algorithms were controlled **via** custom MATLAB scripts and programmed to output patterned intensity-modulated images using the green channel. In brief, the SS or SF (bio)ink was contained in a transparent glass vial with a diameter of 1.2 cm. The vial rotated around its longitudinal axis using a rotation mount at a 6.3° s^−**1**^ of rotation rate. The oxygen content within the SS or SF (bio)ink was allowed to equilibrate prior to printing by storing it in a container exposed to room air for several days at 4 °C.

### Printing resolution evaluation

A solid bar featuring a set of separated parallel threads of different thicknesses (from 1 μm to 101 μm) was designed and printed for print resolution evaluation. The thread thickness was defined as the mean thickness of every single thread at the surface of the solid bar; first, the thread thickness (projected thickness of the entire conical shape) was observed, and then images were taken using an inverted fluorescence microscope at the suitable focal plane (Ti-E, Nikon, Japan) and measured using ImageJ (National Institutes of Health, USA).

### Jaccard similarity index calculation

Jaccard similarity index is a measure of similarity for two samples, with a range from 0 to 100%^43^. The higher percentage means better similarity. The Jaccard similarity indices between the CADs and the resulting prints were calculated by a customized Python (version 3.8, USA) program.

### Absorption characterization

All samples were measured using UV-VIS spectrophotometry (SpectraMax M3, USA) at a wavelength from 400 nm to 700 nm. The thickness of the samples used in the absorbance measurements was 2.64 mm.

### Penetration depth assessment

The penetration depth (D_P_) is defined as the depth at which the intensity of the radiation inside the material falls to 1/e (roughly 37%) of its original value at (or more properly, just beneath) the surface. D_P_ of 525-nm projected light into SS or SF with or without Ru/SPS was calculated as follow:

1 $${{{{{{\boldsymbol{D}}}}}}}_{{{{{{\boldsymbol{P}}}}}}}{{{{{\boldsymbol{=}}}}}}{{{{{\bf{1}}}}}}{{{{{\boldsymbol{/}}}}}}{{{{{\boldsymbol{\alpha }}}}}}$$

2 $${{{{{\boldsymbol{\alpha }}}}}}={{{{{\boldsymbol{ln}}}}}}\,\left({{{{{\bf{10}}}}}}\right)\times ({{{{{\boldsymbol{A}}}}}}/{{{{{\boldsymbol{t}}}}}}),$$

where A is the absorbance value of materials at 525-nm wavelength (For VAM, the printing strategy relies on the penetration depth of the projection light; thus, the lower absorption of the Ru/SPS system at 525 nm is more desirable for VAM than at 400-450 nm, although at the expense of reduced photocrosslinking efficiency.), and t is the height of the tested material.

### M_w_ measurement of silk materials

M_w_ of SF (15 and 30 min-degummed) and SS were determined using sodium dodecyl sulphate-polyacrylamide gel electrophoresis (SDS-PAGE). In a typical experiment, the Bis-Tris gel was run in a gel electrophoresis mini-chamber. Then, SDS PAGE was performed^44^.

### Post-printing processing for inducing shrinkage and expansion

The volumetrically printed SS constructs were immersed in 100% ethanol aqueous solution to induce shrinkage for least 2 h (if not specially noted) at room temperature. For inducing expansion, the same shrunken SS constructs were put in water solution for at least 2 h (if not specially noted).

### Shrinkage and expansion property evaluations

The dimensional changes in diameter, width and height of a given shape in the as-printed, shrunken, and expanded states were measured by a vernier caliper; and the measured data were used to evaluate the shrinking and expanding behaviors of the volumetrically printed silk constructs. The printing parameters for SS structures were 2.5 and 5% SS, 0.5-mM Ru/5-mM SPS, and 3 mW cm^−**2**^ of light intensity; and for SF structures they were 2.5-10% SF, 0.25-mM Ru/2.5-mM SPS, and 3 mW cm^−**2**^ of light intensity. The shrinking behavior of the printed SS objects was mainly induced by 2 h of 100% ethanol solution treatment; and the shrinking behavior of the printed silk cylinders was mainly induced by 24 h of 70% ethanol solution treatment followed by 72 h of air-drying to remove the remaining ethanol. The expanding behavior of the printed SS objects was mainly induced by 2 h of water solution treatment, where the dimensional changes of the structures in the as-printed, shrunken, and expanded states were calculated. For evaluating pore sizes of C60 shapes and resolution changes of solid rod with bars in the shrunken state (0-24 h of 100% ethanol solution treatment, in the wet state) and in the expanded state (0-24 h of water solution treatment after shrinking, in the wet state), similar procedures for inducing shrinking and expanding behavior were performed as mentioned above, and then microscopic images of the volumetrically printed structures in the as-printed, shrunken, and expanded states were taken on an inverted fluorescence microscope, and the changes in pore sizes of C60 shapes and thread thickness of the bars in the as-printed, shrunken, and expanded states are used to define the resolution change.

### Shape-memory property evaluation

With the printing parameters of 2.5% SS with 0.5-mM Ru/5-mM SPS, 3 mW cm^−**2**^ of light intensity and printing time: ~80 s, the volumetrically printed SS structures (channel in a cube) were treated without or with immersion in 100% ethanol for 2 h to induce size-shrinkage; then the same un-shrunken and shrunken objects were pressed by force to deformed the shapes, which were then treated without or with immersion in water solution to observe their shape-recovery processes within 1 h, and the heights of the structures were measured by a vernier caliper to quantify the shape-recovery performances.

### Post-printing processing for formation of double-crosslinked networks of SF prints

The volumetrically printed SF objects (without cells) with single photocrosslinked networks were immersed in 70% ethanol aqueous solution to induce the formation of *β*-sheet conformation within the same SF prints for 2 h (only for taking photographs after printing) or 24 h (for all other experiments related to induction of *β*-sheet conformation) at room temperature. After 70% ethanol treatment, the SF prints possessing a double-crosslinked network were left in a hood to allow evaporation of the remaining ethanol for 72 h for further use.

### Mechanical property measurement

Compression and tensile tests were performed on a uniaxial tensile testing system (Instron 3366, USA).

### Raman spectroscopy

Raman spectra were obtained using a confocal Raman spectrometer (XploRA plus, Horiba Scientific) equipped with a 785-nm laser (41.8 mW), a 1200 g mm^−**1**^ of grating, a 50× objective, and a Synapse charge-coupled device (CCD) detector. The confocal hole and the entrance slit were fixed at 500 and 200 μm, respectively. The nominal spectral resolution was 1 cm^−**1**^. The Raman spectra were calibrated using a silicon wafer with a characteristic peak at 520 cm^−**1**^ and were post processed via the LabSpec 6 software (Horiba Scientific), including averaging, DeNoise smoothing, and baseline subtraction.

### FTIR spectroscopy

FTIR analyses were carried out on a FTIR spectrometer (JASCO FTIR 6200, USA). We set 32 scans and a resolution of 4 cm^−1^ to record the spectrum for each test. For each sample, multiple spectra (*n* = 3) were collected, and multiple samples (*n* = 3) were tested and measured in every different experiment condition. The secondary structure contents of SF were determined by performing peak deconvolution over the amide I region (1720 cm^−1^ to 1580 cm^−1^) using the Origin software (OriginLab, USA). Deconvolution was carried out using a secondary derivative method with five primary peaks assigned to a variety of secondary structures respectively: i) *β*-sheet (strong, 1618 cm^−1^ to 1629 cm^−1^), ii) random coil (1630 cm^−1^ to 1657 cm^−1^), iii) helix (1658 cm^−1^ to 1667 cm^−1^), iv) *β*-turn (1670 cm^−1^ to 1696 cm^−1^), and v) *β*-sheet (weak, 1696 cm^−1^ to 1673 cm^−1^). The band shape was analyzed with a Gaussian model.

### SEM

The surface and cross-sections morphologies of the volumetrically printed silk constructs were observed with SEM (Ultra 55 field-emission SEM, Carl-Zeiss, Germany). The samples were sputter-coated with a 5-nm layer of Pt/Pd. For each sample, we took at least three images at different fields of views.

### In vitro water-uptake tests

The printed silk constructs (*n* **=** 4) were placed in PBS (37 °C) for 0 min, 15 min, 30 min, 60 min, 180 min, 360 min, 720 min, 1440 min, 2880 min, and 4320 min; the changes in mass signify fluid uptake. Surface water was removed before weighing; the wet weight of the sample was noted as W_s_ and the dry weight of sample as W_d_. The water-uptake (%) was calculated as follow:

3 $${{{{{\bf{Water}}}}}}\,{{{{{\bf{updatke}}}}}}\,\left(\%\right)=\left[\left({{{{{{\bf{W}}}}}}}_{{{{{{\bf{S}}}}}}}-{{{{{{\bf{W}}}}}}}_{{{{{{\bf{d}}}}}}}\right)/{{{{{{\bf{W}}}}}}}_{{{{{{\boldsymbol{s}}}}}}}\right]\times {{{{{\bf{100}}}}}}$$

### In vitro degradation assay

The volumetrically printed silk constructs (*n* = 4) were transferred into PBS, or 5 U mL^−1^ of protease XIV PBS solution, or 40 U mL^−1^ of*α*-chymotrypsin PBS solution in the wells of 48-well plates at 37 °C. The incubation solution was changed with fresh solution every 2 days. At the desired time points, the samples were washed in distilled water 2 times. Then, the remaining dry masses of the samples were measured. The dry weight of each remaining sample at n day was noted as W_n_ and the initial dry weight of each sample at 0 day as W_0_^28^. The residue mass ratio (%) was calculated as follow:

4 $${{{{{\bf{Residue}}}}}}\,{{{{{\bf{mass}}}}}}\,{{{{{\bf{ratio}}}}}}\,\left(\%\right)=\left({{{{{{\boldsymbol{W}}}}}}}_{{{{{{\boldsymbol{n}}}}}}}/{{{{{{\boldsymbol{W}}}}}}}_{{{{{{\boldsymbol{0}}}}}}}\right)\times {{{{{\bf{100}}}}}}$$

### Bioprinting with cells

For validating their bioactive properties, C2C12 were embedded within the SS and SF bioinks. Screw (45 s of printing time for SS + C2C12, 57 s of printing time for SF + C2C12), C60 (SS + C2C12, 57 s of printing time), and channel in cube shape (SS + C2C12, 45 s of printing time) were bioprinted with the parameters of 0.5-mM Ru for SS, 0.25-mM Ru for SF, 5×10^6^ cells mL^−1^, and 3 mW cm^−2^ of light intensity.

### Cell proliferation

The metabolic activities of C2C12, NIH/3T3, and MDA-MB-231 cells were measured by the PrestoBlue reagent according to the manufacturer’s instructions. The cell (5 × 10^6^ cells mL^−1^)-laden volumetrically printed silk hydrogels were washed once with PBS and placed individually in the wells of a 24-well plate, where a working solution composed of the culture medium and the reagent at a proportion of 9:1 (v/v) was added into each well. After incubating for 3 h at 37 °C, the supernatants were read by a spectrophotometer (excitation: 570 nm, emission: 600 nm) (I-control, Tecan, Switzerland) to quantify. Similarly, the metabolic activities of the embedded MSCs were measured by MTS reagent according to the manufacturer’s instructions.

### Live/dead and F-actin imaging

Cell viability was measured by the Live/dead viability/cytotoxicity kit. The cell (5×10^6^ cells mL^−1^)-laden volumetrically printed silk hydrogels were washed with PBS twice and placed individually in the wells of a 24-well plate. Next, a working solution containing 2 μL mL^−1^ of ethidium homodimer-1 and 0.5 μL mL^−1^ of calcein-AM in PBS was added. The samples were incubated at 37 °C in the incubator for 30 min. Finally, the samples were washed with PBS twice and resuspended in PBS for taking fluorescence images on an inverted fluorescence microscope. The numbers of live and dead cells were counted using ImageJ.

For F-actin staining, the volumetrically printed SF screws seeded with MSCs (5 × 10^6^ cells mL^−1^) were fixed at 1, 4, and 7 days with 10% formaldehyde (Sigma-Aldrich, USA) solution for 20 min and permeabilized with 0.1% Triton X-100 in PBS for 10 min at room temperature. After 1 h of blocking with 1% BSA in PBS at 4 °C overnight, MSCs were incubated with a phalloidin PBS solution at a ratio of 1:1000 (v/v) at 4 °C overnight. Lastly, the samples were incubated with a DAPI PBS solution at a ratio of 1:5000 for 15 min. Fluorescence micrographs were obtained by a confocal fluorescence microscope (TCS SP5, Leica, Germany). Similar procedures were used for volumetrically printed SS constructs.

### Cell differentiation

For osteogenesis-related immunostaining, the volumetrically printed SF screws seeded with MSCs (5×10^6^ cells mL^−1^) were cultured in the osteogenic differentiation medium and fixed at 1, 7, 14, 21, and 28 days with 10% formaldehyde solution for 20 min and permeabilized with 0.1% Triton X-100 in PBS for 10 min at room temperature. After 1 h of blocking with 1% BSA in PBS at 4 °C overnight, the samples were separately incubated with the two primary antibody solutions at 4 °C overnight, i.e., mouse anti-RUNX2 antibody PBS solution at a ratio of 1:500 (v/v) and mouse anti-osteocalcin antibody PBS solution at a ratio of 1:1000 (v/v). The samples stained with primary antibodies were then stained with the secondary antibody (goat anti-mouse IgG H&L PBS solution at a ratio of 1:400 (v/v)) after washing with PBS for 3 times. Then, the samples were incubated with a DAPI PBS solution at a ratio of 1:5000 (v/v) for 15 min. For evaluation of calcium deposits during osteogenic differentiation, the volumetrically printed SF screws seeded with MSCs were analyzed with ARS staining at 1, 7, 14, 21, and 28 days. The images were taken with an air-immersion 10X objective (Olympus, Japan) and stitched utilizing ImageJ.

### RNA-isolation and real-time reverse transcription-quantitative polymerase chain reaction (qPCR)

The gene expression levels of the MSC-specific osteogenic marker genes including *ALP*, *Runx*, *COL1α1*, *OCN*, *BMP2*, *Osx*, and *OPN* were examined using qPCR. Nucleotide sequences of qPCR primers are shown in Supplementary Table 4. qPCR procedure was performed as follow: first, RNA of cells digested by trypsinization from the silk screws was extracted using RNeasy mini kit. Then, all the extracted RNA was reversed-transcribed into complementary DNA (cDNA) by using the QuantiTect reverse transcription kit. qPCR was performed using the SYBR™ Green qPCR Master Mix. The expression of each target messenger RNA (mRNA) relative to glyceraldehyde 3-phosphate dehydrogenase (GAPDH) in different days was calculated based on the threshold cycle (Ct) as 2^-Δ(ΔCt)^, where ΔCt = Ct (sample)–Ct (GAPDH) and Δ(ΔCt) = ΔCt (the cells undergoing osteogenic differentiation)–ΔCt (control). The expression at 1 day was set as the control group for each gene. Quantitative gene expression levels were measured from three identical samples at 1, 7, 14, 21, and 28 days. Gene expression plots were generated in the GraphPad Prism software (GraphPad Software, USA).

### Ex vivo implantation test

The volumetrically printed 10% SF screws (0.25-mM Ru/2.5-mM SPS and 3 mW cm^−2^ of light intensity) with a double-crosslinked network were used for the ex vivo implantation test. Fresh porcine femur was bought from a local supermarket; and meat on the femur was removed. A transparent cap with a notch (cut from acrylic boards) was attached onto the head of each SF screw assist with implantation. The femur was pre-drilled using a drill bit (1-mm wide) to create holes (1-mm deep) that were slightly smaller than the diameter of the volumetrically printed SF screws, followed by the implantation of the screws using a screwdriver.

### Micro-CT analysis

For a qualitative 3D evaluation, the porcine femur was examined with a micro-CT X-ray imaging system (X-Tek HMXST 225, Nikon Metrology, Japan) at Harvard University Center for Nanoscale Systems. Micro-CT scanning was performed using a microfocus reflection tungsten X-ray source with tube voltage of 70 kV and target current of 180 µA (12.6 W). 3142 projection images were captured per sample on a 16-bit 2000 × 2000 flat panel detector with each exposure time at 1.0 s. The achievable voxel resolution was 24 μm. Data was later reconstructed using CT Pro 3D software (Nikon Metrology) and exported to images using VGStudio MAX software (Volume Graphics, Germany).

### CAM assay

The *ex ovo* chick CAM culture was undertaken^28^. For preparing the VEGF group, the SS bioink was supplemented with 100 ng mL^−1^ of VEGF (PeproTech). The eggshells were carefully cracked to start the *ex ovo* culture 3 days after incubation. The volumetrically bioprinted SS constructs were implanted in chick embryo CAM on day 7. The samples were then incubated for another 7 days until they were collected for imaging.

We used 10% (v/v) formalin to fix the samples for inspecting the angiogenesis. An optical camera (EOS 60D, Canon) was used for photographing. The discernible BV lengths on the volumetrically bioprinted constructs were measured using the NeuronJ tracing toolbox of ImageJ to quantify the extents of vascularization.

### Statistical analyses

All data were expressed as means ± standard deviations (SDs) for n ≥ 3. GraphPad Prism software was used to perform a one-way analysis of variance (ANOVA) or two-way ANOVA with Tukey’s post hoc multiple comparison test or unpaired t-test to determine statistical significance (*p or *adjusted *p* ≤ 0.05, **p or **adjusted *p* ≤ 0.01, ***p or ***adjusted *p* ≤ 0.001, ****p or ****adjusted *p* ≤ 0.0001).

### Reporting summary

Further information on research design is available in the Nature Portfolio Reporting Summary linked to this article.

## Supplementary information

Supplementary Information

Description of Additional Supplementary Files

Supplementary Movie 1

Supplementary Movie 2

Supplementary Movie 3

Supplementary Movie 4

Supplementary Movie 5

Supplementary Movie 6

Supplementary Movie 7

Supplementary Movie 8

Supplementary Movie 9

Supplementary Movie 10

Supplementary Movie 11

Reporting Summary

## Source data

Source Data

## Data Availability

All relevant data supporting the key findings of this study are available within the article and its Supplementary Information files as well as Source Data. Additional datasets are available from the corresponding author upon request. Source data are provided with this paper.

## Code availability

The codes developed in this study are available from the corresponding author upon request.

## References

[1] Liang, K., Carmone, S., Brambilla, D. & Leroux, J. C. 3D printing of a wearable personalized oral delivery device: A first-in-human study. *Sci. Adv.* **4**, eaat2544 (2018). 29750201 10.1126/sciadv.aat2544 PMC5942915

[2] Martin, J. H. et al. 3D printing of high-strength aluminium alloys. *Nature* **549**, 365–369 (2017). 28933439 10.1038/nature23894

[3] Murphy, S. V., De Coppi, P.& Atala, A. Opportunities and challenges of translational 3D bioprinting. *Nat. Biomed. Eng*, 1–11 (2019). 10.1038/s41551-019-0471-7 31695178

[4] Moroni, L. et al. Biofabrication strategies for 3D in vitro models and regenerative medicine. *Nat. Rev. Mater.* **3**, 21–37 (2018). 31223488 10.1038/s41578-018-0006-y PMC6586020

[5] Heinrich, M. A. et al. 3D Bioprinting: from Benches to Translational Applications. *Small* **15**, 1805510 (2019). 10.1002/smll.201805510 PMC6752725 31033203

[6] Ravanbakhsh H. et al. Emerging Technologies in Multi-Material Bioprinting. *Adv. Mater.* **33**, 2104730 (2021). 10.1002/adma.202104730 PMC8971140 34596923

[7] Kelly, B. E. et al. Volumetric additive manufacturing via tomographic reconstruction. *Science* **363**, 1075–1079 (2019). 30705152 10.1126/science.aau7114

[8] Cook, C. C. et al. Highly Tunable Thiol-Ene Photoresins for Volumetric Additive Manufacturing. *Adv. Mater.* **32**, e2003376 (2020). 33002275 10.1002/adma.202003376

[9] Loterie, D., Delrot, P. & Moser, C. High-resolution tomographic volumetric additive manufacturing. *Nat. Commun.* **11**, 852 (2020). 32051409 10.1038/s41467-020-14630-4 PMC7015946

[10] Fan, L. P., Li, J. L., Cai, Z. X., & Wang, X. G. Bioactive hierarchical silk fibers created by bioinspired self-assembly. *Nat. Commun.* **12**, 2375 (2021). 10.1038/s41467-021-22673-4 PMC8062673 33888723

[11] Xu, S. et al. Genetically engineered pH-responsive silk sericin nanospheres with efficient therapeutic effect on ulcerative colitis. *Acta Biomater*, (2022). 10.1016/j.actbio.2022.03.012 35288310

[12] Xu, S. et al. Fabrication of a Silk Sericin Hydrogel System Delivering Human Lactoferrin Using Genetically Engineered Silk with Improved Bioavailability to Alleviate Chemotherapy-Induced Immunosuppression. *ACS Appl Mater. Interfaces* **13**, 45175–45190 (2021). 34525798 10.1021/acsami.1c08409

[13] Chen, C. S. et al. Three-Dimensionally Printed Silk-Sericin-Based Hydrogel Scaffold: A Promising Visualized Dressing Material for Real-Time Monitoring of Wounds. *ACS Appl Mater. Interfaces* **10**, 33879–33890 (2018). 30204403 10.1021/acsami.8b10072

[14] Mu, X., Fitzpatrick, V. & Kaplan, D. L. From Silk Spinning to 3D Printing: Polymer Manufacturing using Directed Hierarchical Molecular Assembly. *Adv. Health. Mater.* **9**, e1901552 (2020). 10.1002/adhm.201901552 PMC7415583 32109007

[15] Xue, B. et al. Curcumin-Silk Fibroin Nanoparticles for Enhanced Anti-Candida albicans Activity In Vitro and In Vivo. *J. Biomed. Nanotechnol.* **15**, 769–778 (2019). 30841969 10.1166/jbn.2019.2722

[16] Mu X. et al. Recent Advances in 3D Printing with Protein-Based Inks. *Prog. Polym. Sci.* **115**, 101375 (2021). 10.1016/j.progpolymsci.2021.101375 PMC7996313 33776158

[17] Xie, M. et al. An implantable and controlled drug-release silk fibroin nanofibrous matrix to advance the treatment of solid tumour cancers. *Biomaterials* **103**, 33–43 (2016). 27376557 10.1016/j.biomaterials.2016.06.049

[18] Zheng, Z. et al. 3D Bioprinting of Self-Standing Silk-Based Bioink. *Adv. Health. Mater.* **7**, e1701026 (2018). 10.1002/adhm.201701026 29292585

[19] Schacht, K. et al. Biofabrication of cell-loaded 3D spider silk constructs. *Angew. Chem. Int Ed. Engl.* **54**, 2816–2820 (2015). 25640578 10.1002/anie.201409846

[20] Sakai, S., Yoshii, A., Sakurai, S., Horii, K. & Nagasuna, O. Silk fibroin nanofibers: a promising ink additive for extrusion three-dimensional bioprinting. *Mater. Today Bio* **8**, 100078 (2020). 33083780 10.1016/j.mtbio.2020.100078 PMC7552084

[21] Rodriguez, M. J. et al. 3D freeform printing of silk fibroin. *Acta Biomater.* **71**, 379–387 (2018). 29550442 10.1016/j.actbio.2018.02.035 PMC5899947

[22] Hong, H. et al. Digital light processing 3D printed silk fibroin hydrogel for cartilage tissue engineering. *Biomaterials* **232**, 119679 (2020). 31865191 10.1016/j.biomaterials.2019.119679

[23] Kim, S. H. et al. Precisely printable and biocompatible silk fibroin bioink for digital light processing 3D printing. *Nat. Commun.* **9**, 1620 (2018). 29693652 10.1038/s41467-018-03759-y PMC5915392

[24] Mu, X., Sahoo, J. K., Cebe, P. & Kaplan, D.L. Photo-Crosslinked Silk Fibroin for 3D Printing. *Polymers (Basel)* **12**, 2936 (2020). 10.3390/polym12122936 PMC7763742 33316890

[25] Cui, X. et al. Rapid Photocrosslinking of Silk Hydrogels with High Cell Density and Enhanced Shape Fidelity. *Adv. Health. Mater.* **9**, e2001801 (2020). 10.1002/adhm.202001801 33205605

[26] Brif, A., Laity, P., Claeyssens, F. & Holland, C. Dynamic photo-cross-linking of native silk enables macroscale patterning at a microscale resolution. *ACS Biomater. Sci. Eng.* **6**, 705–714 (2020). 33463209 10.1021/acsbiomaterials.9b00993

[27] Su, D. et al. Enhancing Mechanical properties of silk fibroin hydrogel through restricting the growth of beta-sheet domains. *ACS Appl Mater. Interfaces* **9**, 17489–17498 (2017). 28470062 10.1021/acsami.7b04623

[28] Hossein, R. et al. Freeform cell-laden cryobioprinting for shelf-ready tissue fabrication and storage. *Matter* **5**, 573–593 (2021). 35695821 10.1016/j.matt.2021.11.020 PMC9173715

[29] Kim, H. et al. Light-Activated Decellularized Extracellular matrix-based bioinks for volumetric tissue analogs at the centimeter scale. *Adv. Func. Mater.* **31**, 2011525 (2021).

[30] Lim, K. S. et al. The influence of silkworm species on cellular interactions with novel PVA/silk sericin hydrogels. *Macromol. Biosci.* **12**, 322–332 (2012). 22493796 10.1002/mabi.201100292

[31] Lim, K. S. et al. Promoting cell survival and proliferation in degradable poly(vinyl alcohol)-tyramine hydrogels. *Macromol. Biosci.* **15**, 1423–1432 (2015). 26097045 10.1002/mabi.201500121

[32] Qin, N., Qian, Z. G., Zhou, C., Xia, X. X. & Tao, T. H. 3D electron-beam writing at sub-15 nm resolution using spider silk as a resist. *Nat. Commun.* **12**, 5133 (2021). 34446721 10.1038/s41467-021-25470-1 PMC8390743

[33] Bernal, P. N. et al. Volumetric Bioprinting of Complex Living-Tissue Constructs within Seconds. *Adv. Mater.* **31**, e1904209 (2019). 31423698 10.1002/adma.201904209

[34] Wang, M., Li, W., Tang, G., Garciamendez, C. & Zhang, Y. Engineering (Bio)Materials through Shrinkage and Expansion. *Adv. Health. Mater.* **10**, 2100380 (2021). 10.1002/adhm.202100380 PMC8295236 34137213

[35] Guo, C. et al. Thermoplastic moulding of regenerated silk. *Nat. Mater.* **19**, 102–108 (2020). 31844276 10.1038/s41563-019-0560-8 PMC6986341

[36] Kim, D. K. et al. New fabrication method of silk fibroin plate and screw based on a centrifugal casting technique. *J. Tissue Eng. Regen. Med.* **12**, 2221–2229 (2018). 30265448 10.1002/term.2752

[37] Xie, M. et al. Supercritical carbon dioxide-developed silk fibroin nanoplatform for smart colon cancer therapy. *Int J. Nanomed.* **12**, 7751–7761 (2017). 10.2147/IJN.S145012 PMC5659230 29118580

[38] Fitzpatrick, V. et al. Functionalized 3D-printed silk-hydroxyapatite scaffolds for enhanced bone regeneration with innervation and vascularization. *Biomaterials* **276**, 120995 (2021). 34256231 10.1016/j.biomaterials.2021.120995 PMC8408341

[39] Qi, C. et al. Photo-crosslinkable, injectable sericin hydrogel as 3D biomimetic extracellular matrix for minimally invasive repairing cartilage. *Biomaterials* **163**, 89–104 (2018). 29455069 10.1016/j.biomaterials.2018.02.016

[40] Mu, X. et al. 3D Printing of Silk Protein Structures by Aqueous Solvent-Directed Molecular Assembly. *Macromol. Biosci.* **20**, e1900191 (2020). 31433126 10.1002/mabi.201900191 PMC6980242

[41] Zhao, Z., Li, Y. & Xie, M. B. Silk fibroin-based nanoparticles for drug delivery. *Int J. Mol. Sci.* **16**, 4880–4903 (2015). 25749470 10.3390/ijms16034880 PMC4394455

[42] Pritchard, E. M., Hu, X., Finley, V., Kuo, C. K. & Kaplan, D. L. Effect of silk protein processing on drug delivery from silk films. *Macromol. Biosci.* **13**, 311–320 (2013). 23349062 10.1002/mabi.201200323 PMC3761156

[43] Tang, M. et al. Evaluating single-cell cluster stability using the Jaccard similarity index. *Bioinformatics* **37**, 2212–2214 (2021). 33165513 10.1093/bioinformatics/btaa956 PMC8352506

[44] Sahoo, J. K. et al. Silk degumming time controls horseradish peroxidase-catalyzed hydrogel properties. *Biomater. Sci.* **8**, 4176–4185 (2020). 32608410 10.1039/d0bm00512f PMC7390697

