# Supplementary material for: Volumetric additive manufacturing of pristine silk-based (bio)inks
Source: Nat Commun. 2023 Jan 13;14:210. doi: 10.1038/s41467-023-35807-7 (PMC9839706; doi:10.1038/s41467-023-35807-7)
Supplement: Supplementary file 1 — Supplementary Information [file 41467_2023_35807_MOESM1_ESM.pdf]

Supplementary Information for

Volumetric Additive Manufacturing of Pristine Silk-Based (Bio)inks

Maobin Xie[†], Liming Lian[†], Xuan Mu, Zeyu Luo, Carlos Ezio Garciamendez-Mijares, Zhenrui Zhang, Arturo López, Jennifer Manríquez, Xiao Kuang, Junqi Wu, Jugal Kishore Sahoo, Federico Zertuche González, Gang Li, Guosheng Tang, Sushila Maharjan, Jie Guo, David L. Kaplan, Yu Shrike Zhang[*]

*Corresponding author. Email: yszhang@research.bwh.harvard.edu

This PDF file includes:

Supplementary Figures 1 to 39

Supplementary Tables 1 to 4

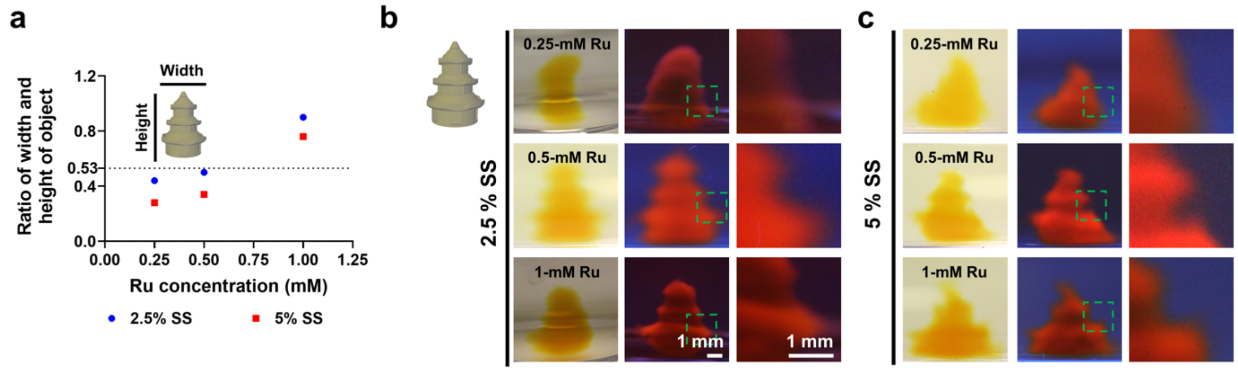

Supplementary Fig. 1.

Printability of SS (bio)ink with different formulations. (**a**) Ratios of width to height of the volumetric prints (temple of heaven). The value close to 0.53 indicated matching that of the designed CAD model. (**b**, **c**) Printability of 2.5% and 5% SS with different Ru concentrations. SS: silk sericin. Ru: ruthenium (II) hexahydrate.

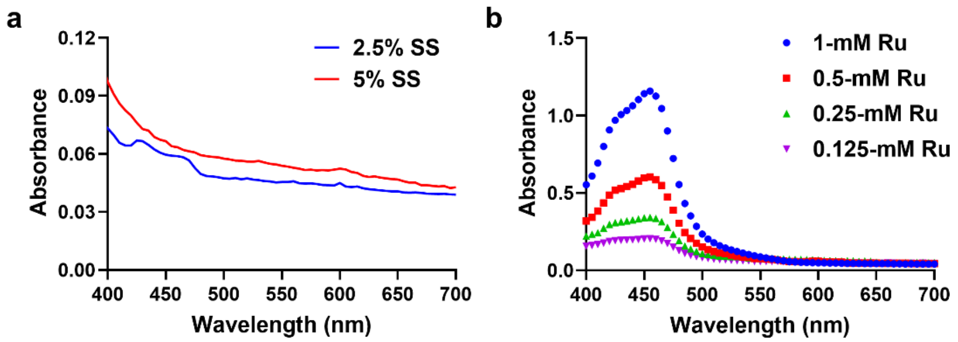

Supplementary Fig. 2.

Absorbance profiles of the SS (bio)ink formulations. (**a**) Absorbance profiles of 2.5% and 5% SS at wavelengths from 400 nm to 700 nm. (**b**) Absorbance profiles of Ru with different concentrations at wavelengths from 400 nm to 700 nm. SS: silk sericin. Ru: ruthenium (II) hexahydrate.

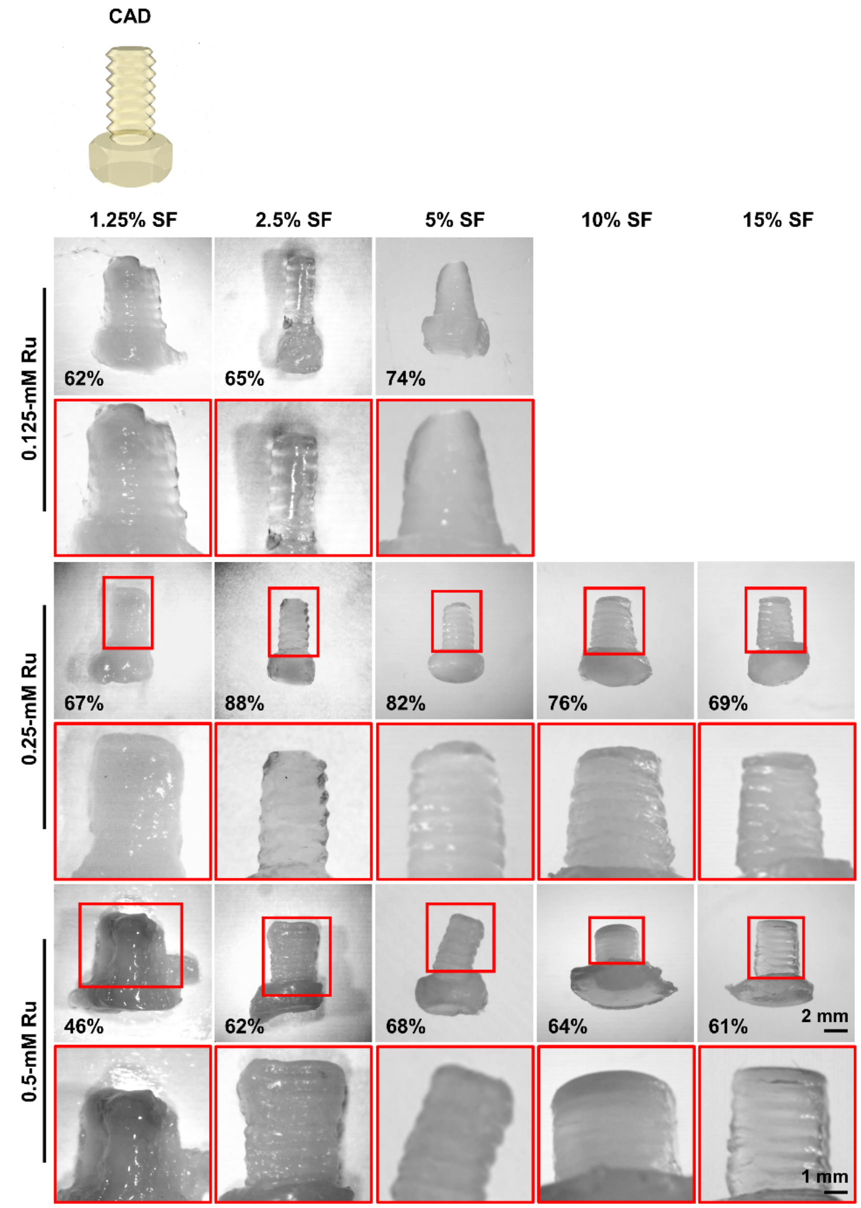

Supplementary Fig. 3.

Printing performance of SF (bio)ink formulations. CAD and photographs of volumetrically printed SF screws with different SF (bio)ink formulations. The printing parameters were 1.25-15% SF, 0.125-0.5 mM of Ru/1.25-5 mM of SPS, and 3 mW cm^{-2} of light intensity. The numbers in the images represent the Jaccard similarity indices between the CADs and the corresponding resulting prints. SF: silk fibroin. Ru: ruthenium (II) hexahydrate. CAD: computer-aided design.

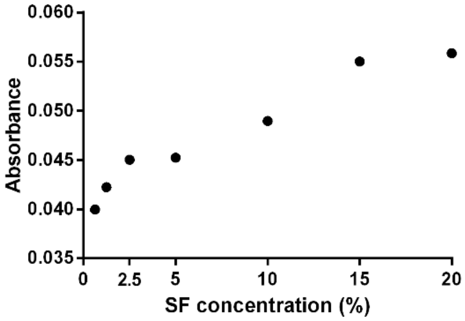

Supplementary Fig. 4.

Absorbance value comparison of the SF (bio)ink formulations with different SF concentrations at 525 nm of wavelength. SF: silk fibroin.

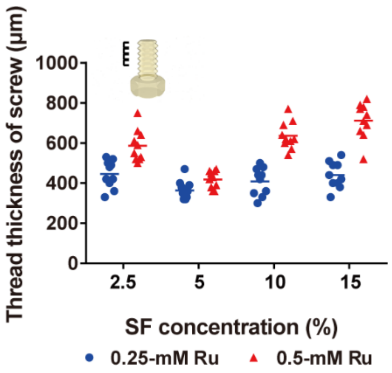

Supplementary Fig. 5.

Thread thicknesses of the printed SF screws with different SF (bio)ink formulations. The smaller values indicate the better resolutions. SF: silk fibroin. Ru: ruthenium (II) hexahydrate. *n*=10 independent experiments.

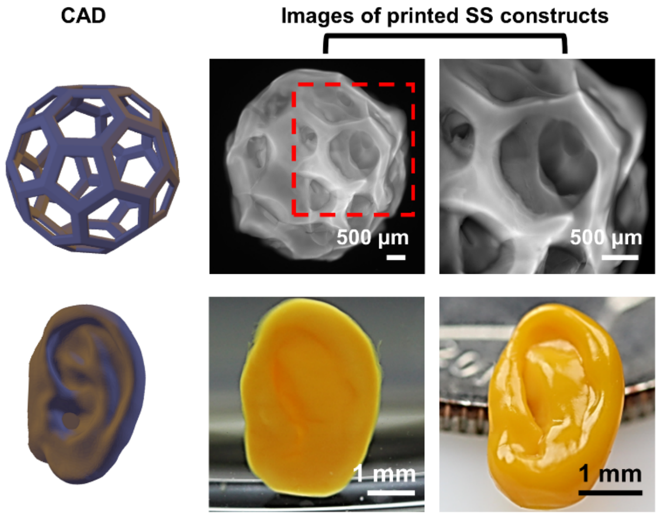

Supplementary Fig. 6.

Printing performances of SS (bio)ink. CAD images of C60 and ear-like structures; microscopic images of printed C60 (Jaccard similarity index at 67%) and ear-like (Jaccard similarity index at 92%) structures with parameters of 2.5% SS, 0.5-mM Ru/5-mM SPS, 3 mW cm^{-2} of light intensity and 57 s of printing time. SS: silk sericin. CAD: computer-aided design.

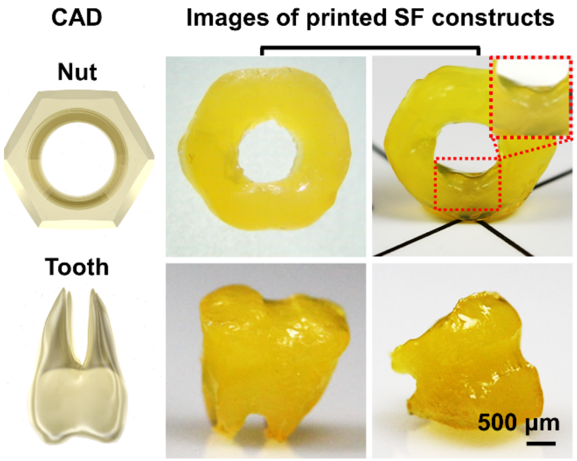

Supplementary Fig. 7.

Photographs of printed SF constructs. CADs and photographs of volumetrically printed SF objects. Nut construct: 10% SF, printing time: ~228 s, Jaccard similarity index at 90%; tooth-like construct: 5% SF, printing time: ~168 s, Jaccard similarity index at 90%. The printing parameters were 0.25-mM Ru/2.5-mM SPS, and 3 mW cm^{-2} of light intensity. SF: silk fibroin. CAD: computer-aided design.

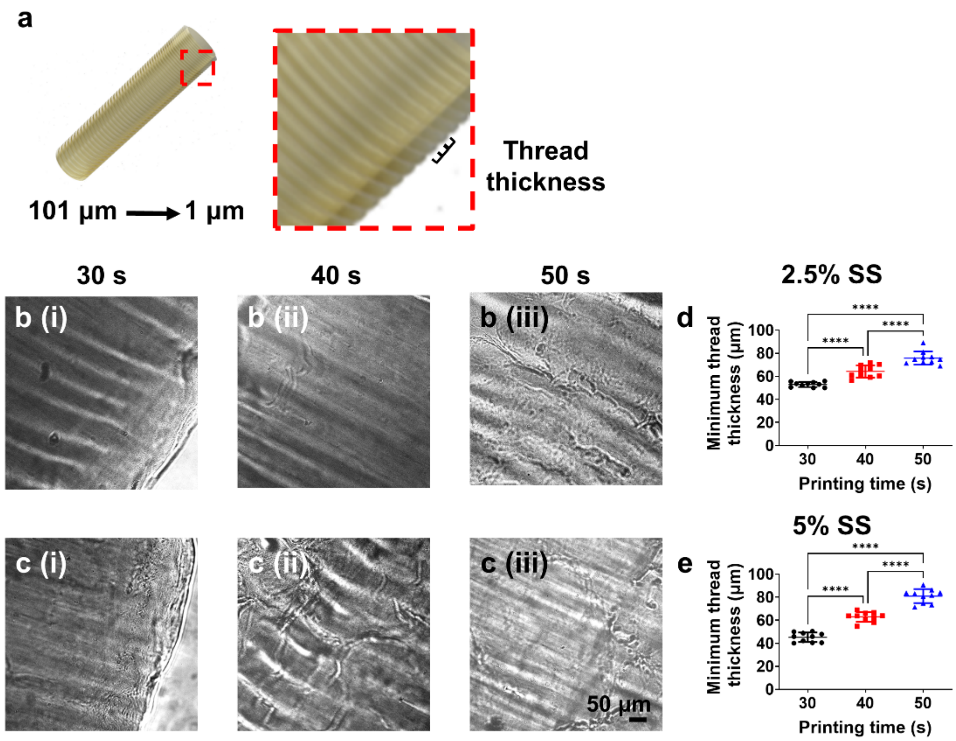

Supplementary Fig. 8.

Resolution with VAM printing of SS (bio)ink formulations. (a) CAD image of a solid bar with threads. (**b(i)-b(iii) and c(i)-c(iii)**) Microscopic images of printed solid bars of 2.5% and 5% SS at different times (30 s, 40 s, and 50 s). The printing parameters were 0.5-mM Ru/5-mM SPS and 3 mW cm^{-2} of light intensity. (**d and e**) Comparisons of minimum thickness of threads printable observed from images in **b(i)-b(iii)** and **c(i)-c(iii)**. SS: silk sericin. Statistical significances are expressed as ****p<0.0001. One-way ANOVA. Data are presented as mean values ± SDs. n=10 independent experiments.

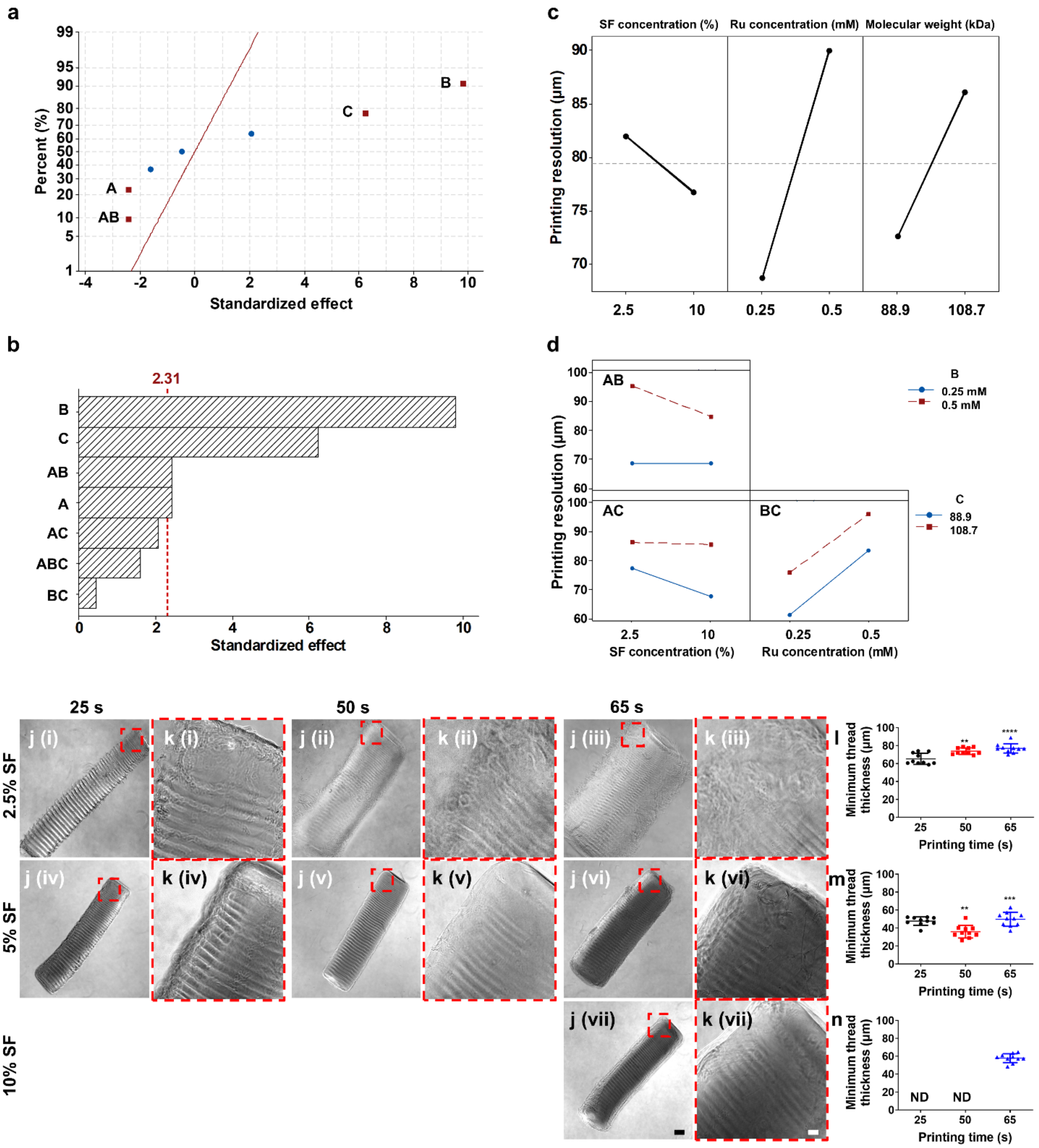

Supplementary Fig. 9.

Full-factorial analyses of three SF factors on volumetric printing resolution. (**a**) Normal plot of the standardized effects. The standardized effect means the importance of factors to the response value (*i.e.*, printing resolution). Response is volumetric printing resolution, α=0.05. The red squares indicate that these effectors had a significant impact on printing resolution. The blue circles indicate these effectors had an insignificant impact on printing resolution. A: SF concentration, B:

Ru concentration, C: M_w of SF. (**b**) Pareto chart of standardized effects of SF concentration, M_w of SF, and Ru concentration on volumetric printing resolution. The values passing 2.31 indicate these effectors have significant impacts on volumetric printing resolution. (**c**) Plots suggesting main effectors in volumetric printing resolution. (**d**) Interaction plots of SF concentration, M_w of SF, and Ru concentration effects on volumetric printing resolution. A: SF concentration, B: Ru concentration, C: M_w of SF. (**j**(**i**)-**j**(**vii**) **and k**(**i**)-**k**(**vii**)) Microscopic images of printed solid bars of 2.5%, 5%, and 10% SF at different printing times (25 s, 50 s, and 65 s). The printing parameters were 0.25-mM Ru/2.5-mM SPS, 88.9-kDa SF, 3 mW cm^{-2} of light intensity. Scale bar in **j(i)-j(vii)**: 500 μm; in **k(i)-k(vii)**: 100 μm. (**l-n**) Comparisons of minimum thicknesses of threads printable observed from images in **k(i)-k(vii)**. SS: silk sericin. SF: silk fibroin. Ru: ruthenium (II) hexahydrate. Statistical significances are expressed as $^{**}p<0.01$, $^{***}p<0.001$, and $^{****}p<0.0001$, compared to the 25-s group for each SF concentration. One-way ANOVA. Data are presented as mean values ± SDs. n=10 independent experiments.

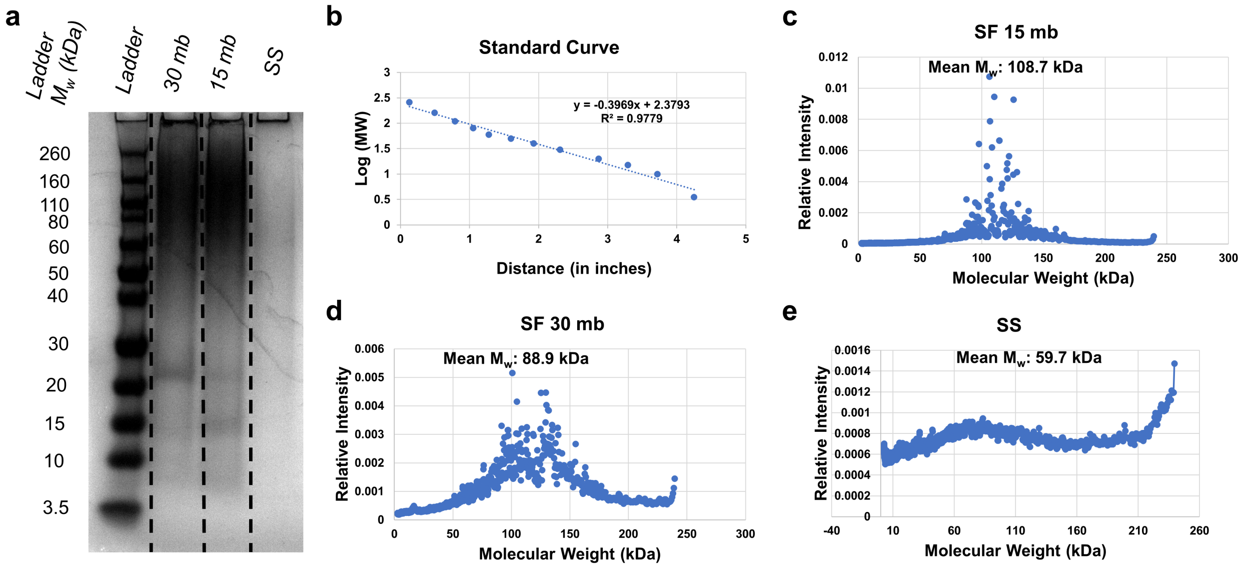

Supplementary Fig. 10.

Molecular weights of silk materials. (**a**) Representative polyacrylamide gel images of SF (15 and 30 min-boiled, *i.e.,* 15 mb and 30 mb) and SS with the reference protein ladder. (**b**) Standard curve was generated by plotting log-scale molecular weights (M_w) from the reference ladder versus distance in inches from the starting point in the lanes. The relation between the log (M_w) and distance was determined by simple linear regression analysis ($R^2 = 0.9779$) and the resulting linear equation was used to convert distance in gel images in inches to molecular weight. (**c and d**) Molecular weight distributions of 15-mb and 30-mb SF indicating mean M_w at 108.7 kDa and 88.9 kDa, respectively. (**e**) Molecular weight distribution of SS indicating mean M_w at 59.7 kDa. SS: silk sericin. SF: silk fibroin. M_w: molecular weight. n=3 independent experiments.

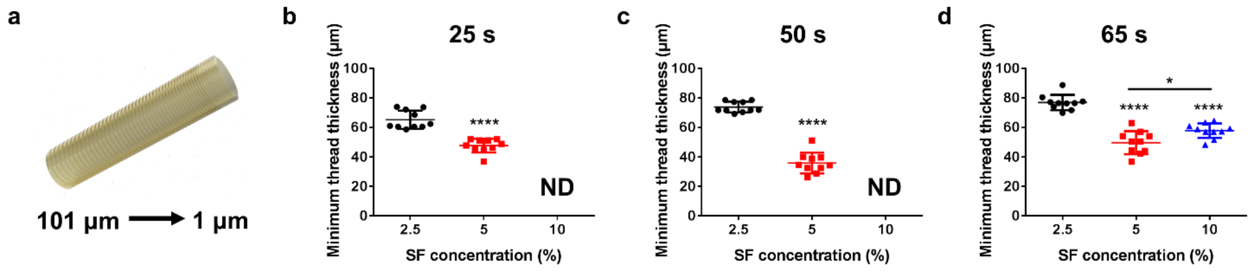

Supplementary Fig. 11.

Resolution with VAM printing of SF (bio)ink formulations. (**a**) CAD model of the solid bar, where the thicknesses of the threads were from 1 μm to 101 μm. (**b-d**) Minimum thread thickness comparisons of volumetrically printed solid bars at 2.5%, 5%, and 10% SF under different printing times (25 s, 50 s, and 65 s, respectively), quantified from **Supplementary Fig. 9k(i)-k(vii)**. The printing parameters were 0.25-mM Ru/2.5-mM SPS, 88.9-kDa SF, and 3 mW cm^{-2} of light intensity. SF: silk fibroin. Statistical significances are expressed as *p<0.05 and ****p<0.0001, compared to the 2.5% SF group. One-way ANOVA. Data are presented as mean values ± SDs. n=10 independent experiments.

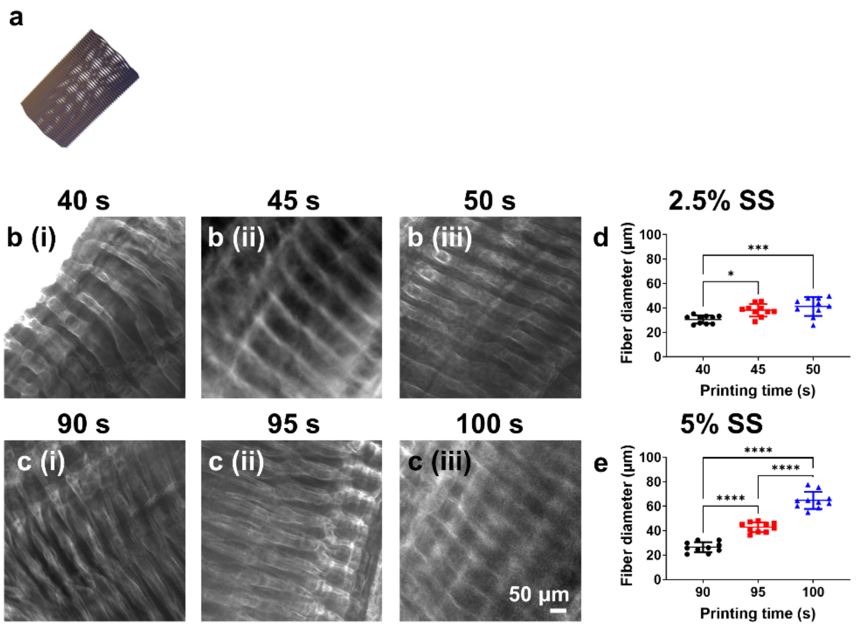

Supplementary Fig. 12.

Resolution with VAM printing of SS (bio)ink formulations. (**a**) CAD image of tubular mesh. (**b(i)-b(iii) and c(i)-c(iii)**) Microscopic images of printed tubular meshes of 2.5% and 5% SS at different times (40 s, 45 s, 50 s, 90 s, 95 s and 100 s). The printing parameters were 0.5-mM Ru/5-mM SPS and 3 mW cm^{-2} of light intensity. (**d and e**) Comparisons of fiber dimeters printable observed from images in **b(i)-b(iii)** and **c(i)-c(iii)**. SS: silk sericin. Statistical significances are expressed as *adjusted p=0.0168, ***adjusted p=0.0008, ****adjusted p<0.0001. One-way ANOVA. Data are presented as mean values ± SDs. n=10 independent experiments.

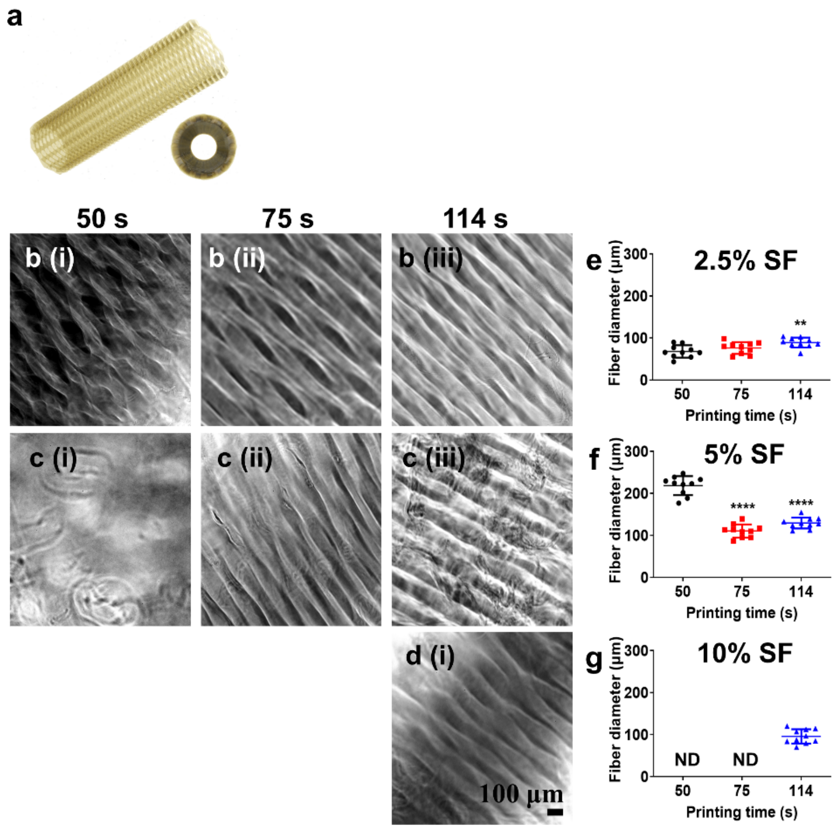

Supplementary Fig. 13

Resolution with VAM printing of SF (bio)ink formulations. (**a**) CAD image of tubular mesh. (**b(i)-b(iii), c(i)-c(iii) and d(i)**) Microscopic images of printed tubular meshes of 2.5%, 5% and 10% SF at different times (50 s, 75 s and 114 s). The printing parameters were 0.25-mM Ru/2.5-mM SPS and 3 mW cm^{-2} of light intensity. (**e-g**) Comparisons of fiber dimeters printable observed from images in **b(i)-d(i)**. SF: silk fibroin. Statistical significances are expressed as $^{**}p<0.01$, $^{****}p<0.0001$, compared to the 50-s group. One-way ANOVA. Data are presented as mean values ± SDs. n=10 independent experiments.

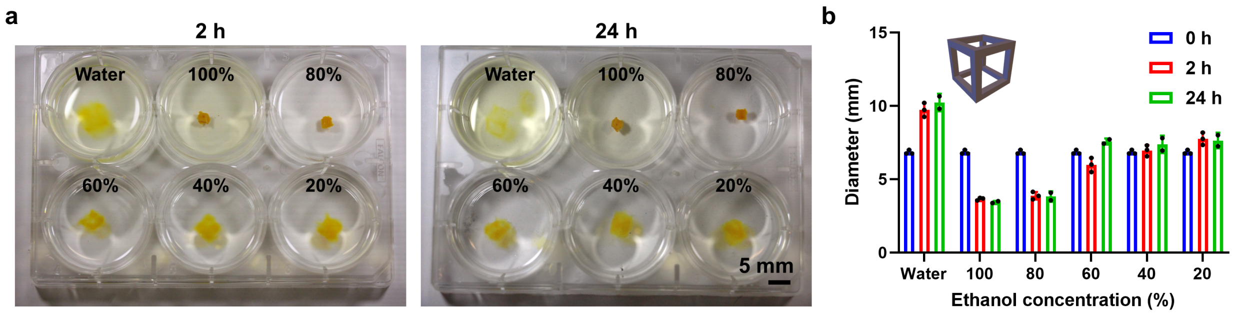

Supplementary Fig. 14.

Effect of different ethanol concentrations on shrinking of SS prints. (**a**) Photographs of the volumetric SS prints (hollow square, 2.5% SS with 0.5-mM Ru/5-mM SPS, 3 mW cm^{-2} of light intensity, printing time: 57 s) immersed in ethanol solution with different concentrations for 2 h and 24 h. (**b**) Diameter changes of the volumetric prints immersed in ethanol solution with different concentrations for 2 h and 24 h. Data are presented as mean values ± SDs. n=3 independent experiments.

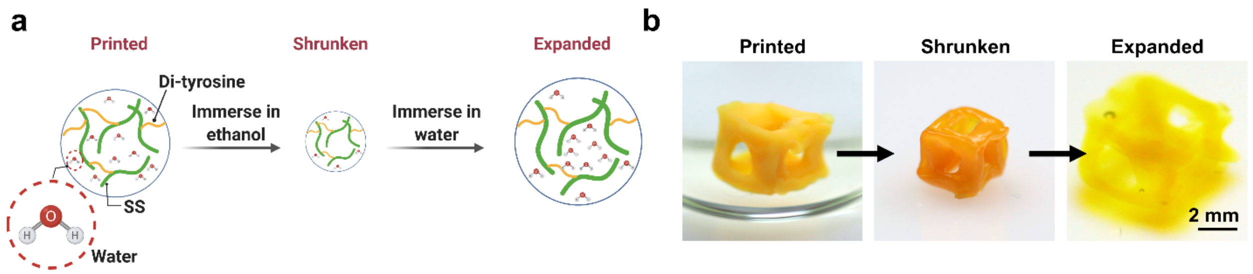

Supplementary Fig. 15.

Shrinkage and expansion properties of volumetrically printed SS objects. (**a**) Proposed swelling mechanism for shrinkage and expansion properties of volumetric printed SS structures. Created with BioRender.com. (**b**) Photographs of volumetrically printed hollow square (57 s of printing time) in the as-printed, shrunken (induced by immersion in 100% ethanol for 2 h), and re-expanded (induced by immersion in 100% ethanol for 2 h first, and then immersion in water for 2 h) states. SS: silk sericin.

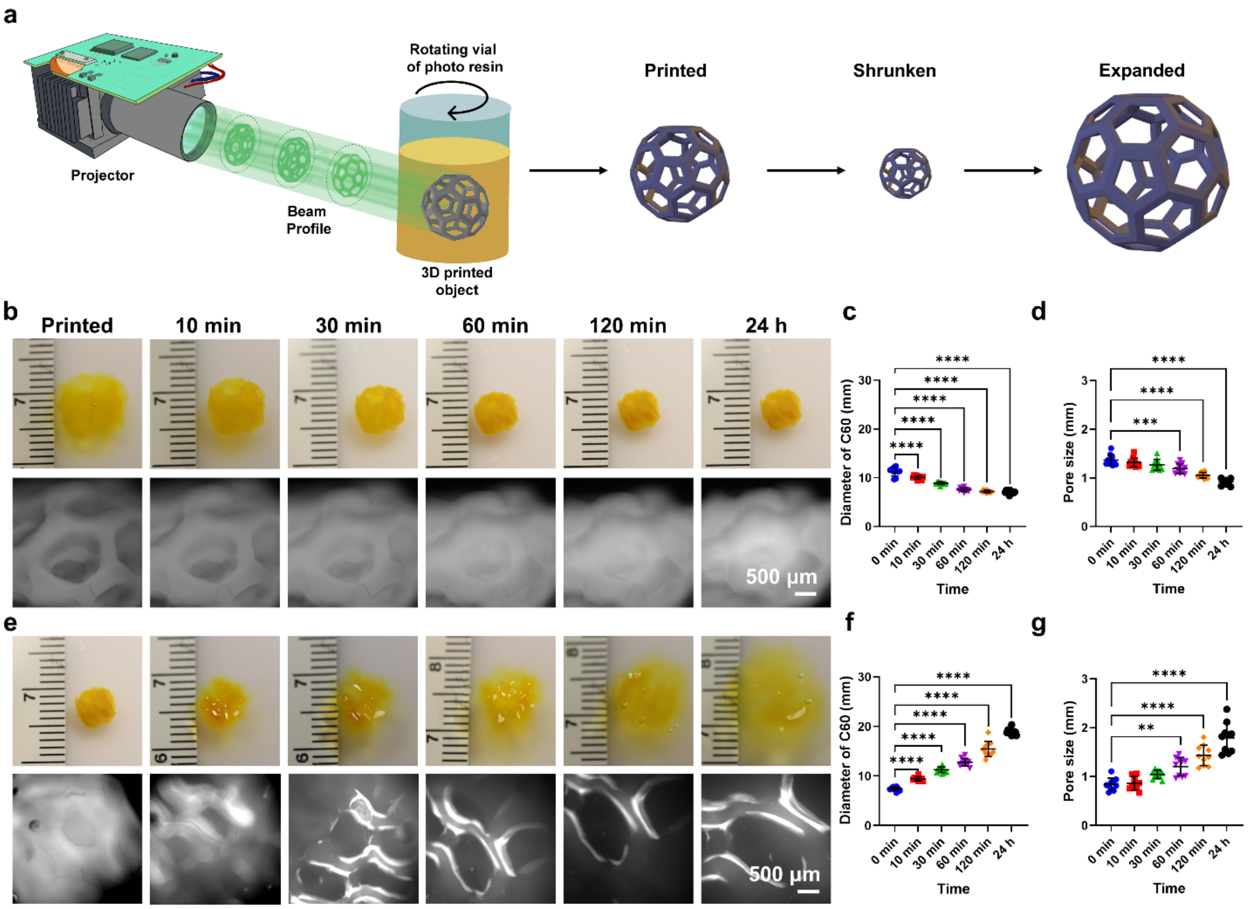

Supplementary Fig. 16.

Reversible shrinkage and expansive property of volumetrically printed SS objects. (**a**) Illustration showing VAM of a C60 structure. Created with BioRender.com. (**b**) Photographs of volumetrically printed SS objects immersed in 100% ethanol for 24 h for inducing shrinkage. Printing parameters: 2.5% SS with 0.5-mM Ru/5-mM SPS, 3 mW cm^{-2} of light intensity, printing time: 57 s. (**c and d**) Diameter and pore size profiles of the printed C60 structure during the shrinking process. (**e**) Photographs of the same shrunken SS object immersed in water for 24 h for inducing re-expansion. (**f and g**) Diameter and pore size profiles of the shrunken C60 structure during the re-expanding process. Statistical significances are expressed as **adjusted p=0.001, ***adjusted p=0.0005, and ****adjusted p<0.0001. One-way ANOVA. Data are presented as mean values ± SDs. n=10 independent experiments.

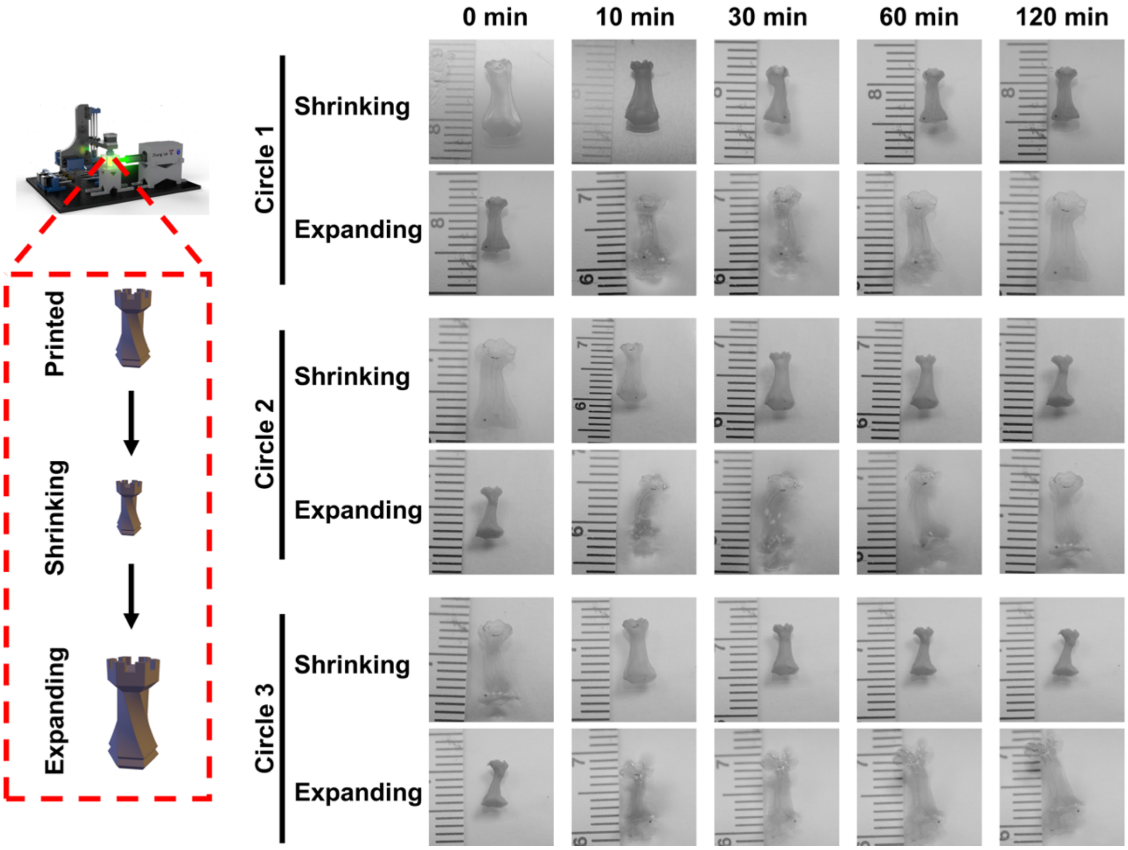

Supplementary Fig. 17.

The repeated shrinking and expanding processes of volumetrically printed SS constructs. Printing parameters: 2.5% SS with 0.5-mM Ru/5-mM SPS, 3 mW cm^{-2} of light intensity, printing time: 57 s. The shrinking process was induced by immersion in 100% ethanol for 2 h; the re-expanding process was induced by immersion in water for 2 h.

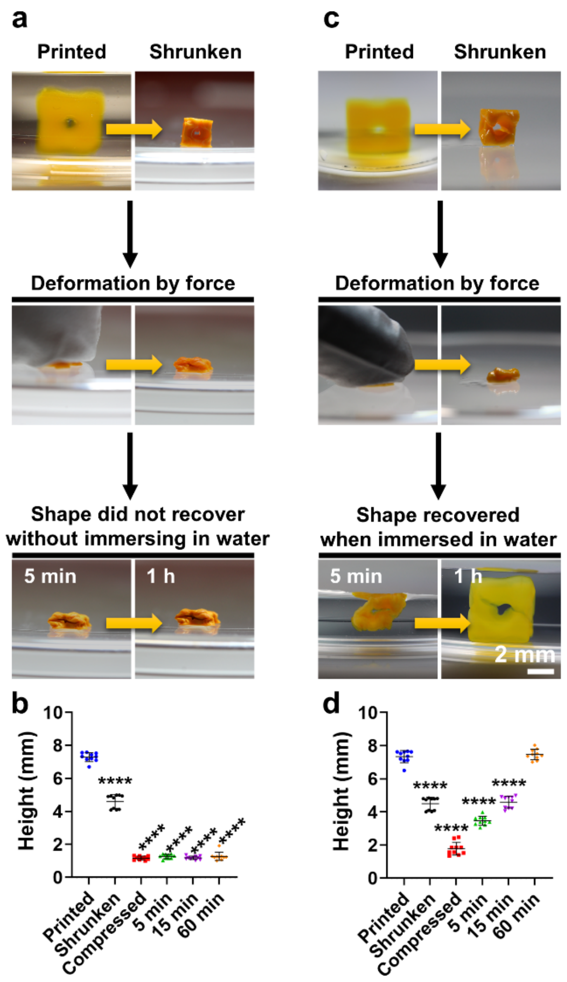

Supplementary Fig. 18.

Shape memory function of volumetrically printed SS objects. Printing parameters: 2.5% SS, 0.5-mM Ru/5-mM SPS, and 3 mW cm^{-2} of light intensity. (**a and c**) Photographs of volumetrically printed SS structures during the shape-memory process. The volumetrically printed a "channel-in-cube" structures were shrunken first (induced by immersion in 100% ethanol for 2 h), then pressed to a deformed shape, and followed by without or with immersion in water to observe their shape-recovery processes. (**b and d**) Height-change profiles of volumetrically printed SS structures during the shape-memory processes. Statistical significances are expressed as ****adjusted $p<0.0001$, where all groups were compared to the printed group. One-way ANOVA. Data are presented as mean values ± SDs. n=10 independent experiments.

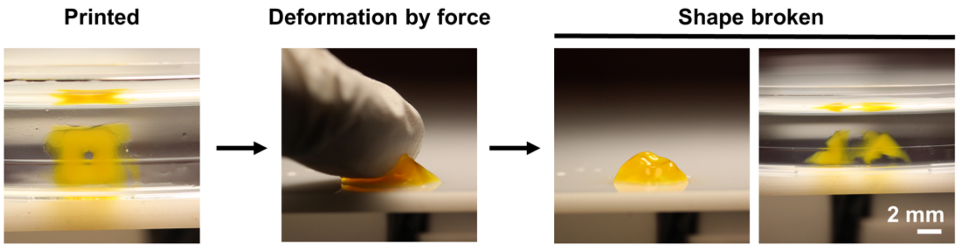

Supplementary Fig. 19.

Shape-memory property of the volumetrically as-printed SS construct. Printing parameters: 2.5% SS with 0.5-mM Ru/5-mM SPS, and 3 mW cm^{-2} of light intensity. The volumetrically as-printed SS construct was directly pressed by force to a deformed, broken shape.

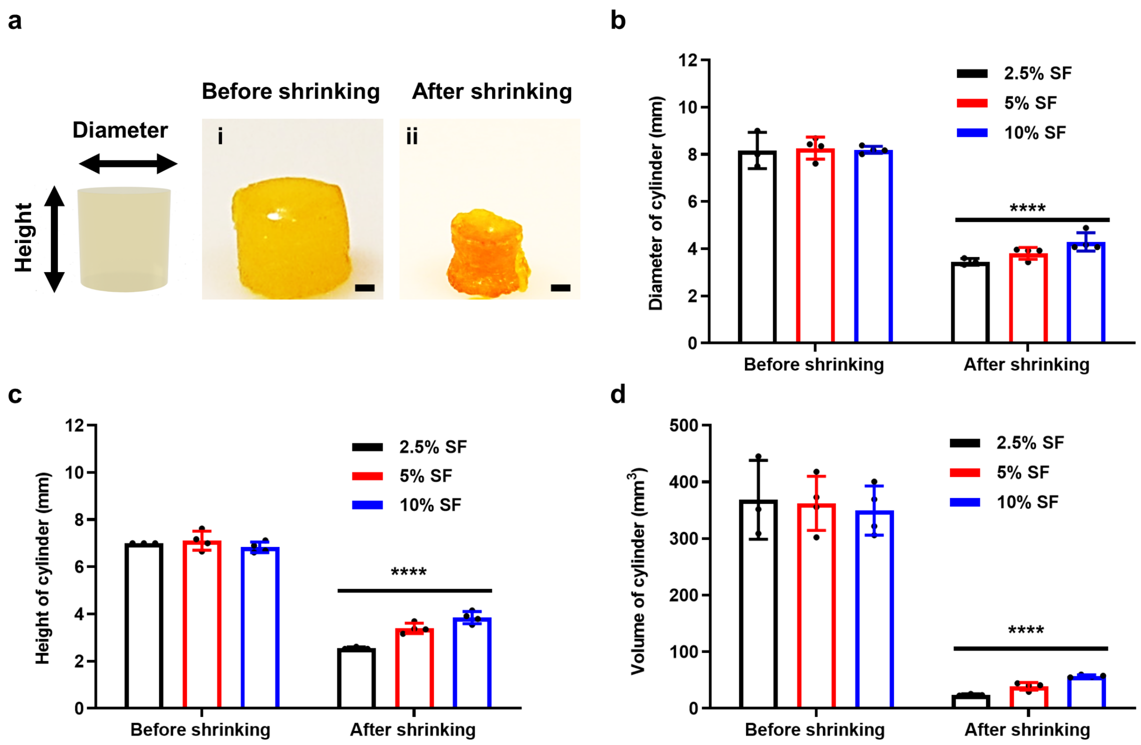

Supplementary Fig. 20.

Dimensional shrinking comparisons of volumetrically printed SF cylinders with a double-crosslinked network. (**a**) CAD model and photographs showing the size-changes of volumetrically printed 2.5% SF cylinders (**i**) before (*i.e.*, after 24 h-ethanol treatment but before air-drying) and (**ii**) after shrinking (*i.e.*, induced by 72 h of air-drying after 24 h-ethanol treatment). The printing parameters were 0.25-mM Ru/2.5-mM SPS, and 3 mW cm^{-2} of light intensity. Scale bar in i: 1.5 mm; in ii: 1 mm. (**b**) Quantified diameter changes of the printed 2.5-10% SF cylinders. (**c**) Quantified height changes of the printed 2.5-10% SF cylinders. (**d**) Quantified volume changes of the printed 2.5-10% SF cylinders. SF: silk fibroin. Statistical significances are expressed as ****adjusted $p<0.0001$, compared to corresponding groups before shrinking. One-way ANOVA. Data are presented as mean values ± SDs. n=4 independent experiments.

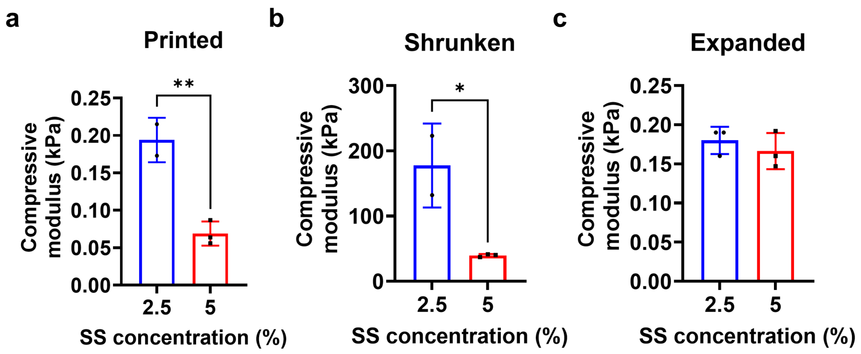

Supplementary Fig. 21.

Mechanical strengths of SS constructs. (**a-c**) Compressive moduli of volumetrically printed structures at different SS concentrations in the printed, shrunken (induced by immersion in 100% ethanol for 2 h), and re-expanded (induced by immersion in 100% ethanol for 2 h first, and then immersion in water for 2 h) states. SS: silk sericin. Statistical significances are expressed as *p=0.0269 and **p=0.0079. Unpaired t-test. Data are presented as mean values ± SDs. n=3 independent experiments.

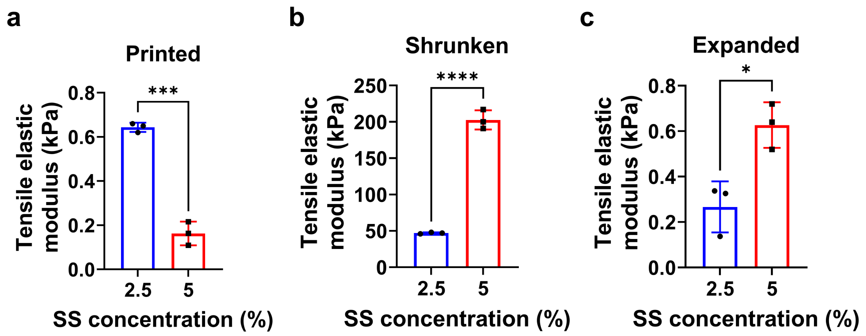

Supplementary Fig. 22.

Mechanical strengths of SS constructs. (**a-c**) Tensile moduli of volumetrically printed structures at different SS concentrations in the printed, shrunken (induced by immersion in 100% ethanol for 2 h), and re-expanded (induced by immersion in 100% ethanol for 2 h first, and then immersion in water for 2 h) states. SS: silk sericin. Statistical significances are expressed as *p=0.0145, ***p=0.0001, and ****p<0.0001. Unpaired t-test. Data are presented as mean values ± SDs. n=3 independent experiments.

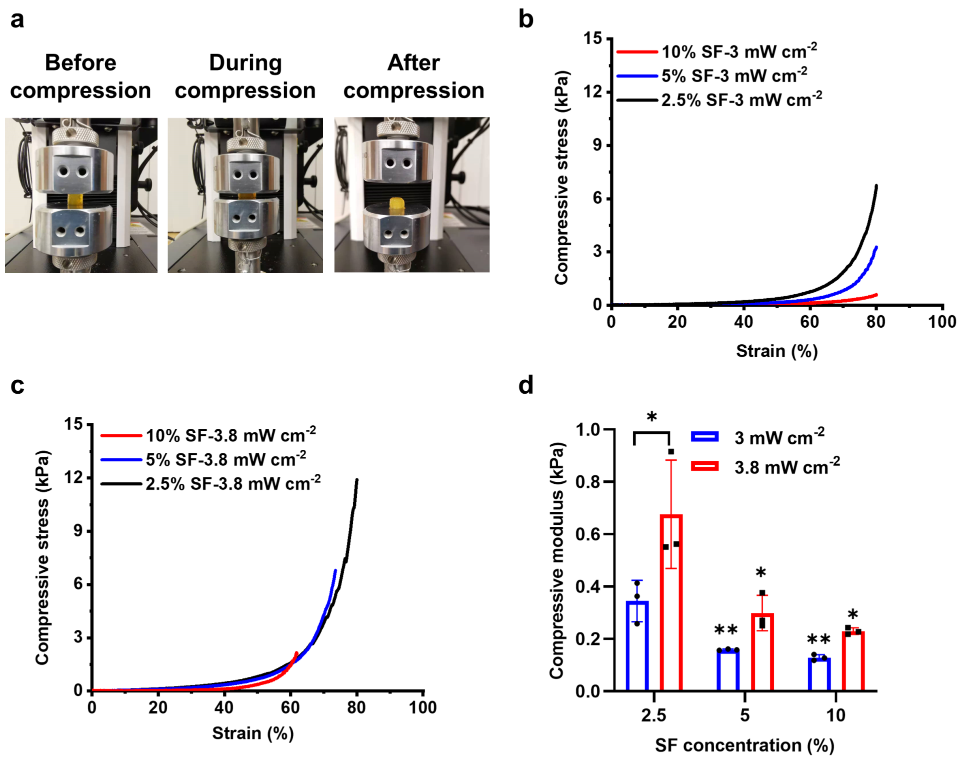

Supplementary Fig. 23.

Compressive strength of volumetrically printed SF cylinders with a single, photocrosslinked network. (**a**) Photographs showing the volumetrically printed 2.5% SF cylinders (0.25-mM Ru/2.5-mM SPS, 88.9-kDa SF, and 3 mW cm^{-2} of light intensity) for the compression test. (**b, c**) Compressive stress-strain curves of 2.5%-10% silk cylinders (0.25-mM Ru/2.5-mM SPS) printed under (**b**) 3 mW cm^{-2} of light intensity and (**c**) 3.8 mW cm^{-2} of light intensity. (**d**) Corresponding compressive modulus comparisons of printed silk cylinders. SF: silk fibroin. Statistical significances are expressed as *p<0.05 and **p<0.01, compared to the 2.5% SF corresponding groups. One-way ANOVA. Data are presented as mean values ± SDs. n=3 independent experiments.

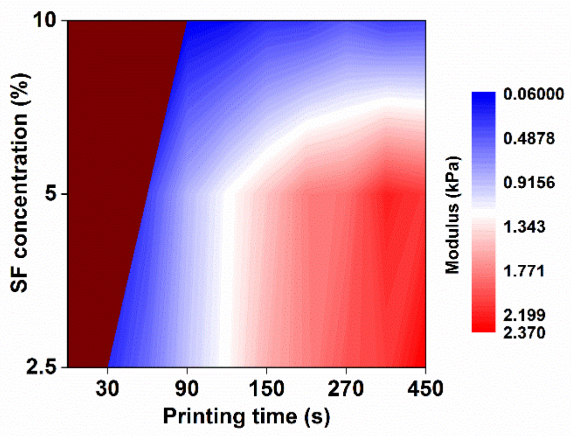

Supplementary Fig. 24.

Surface plot of compressive moduli of volumetrically printed SF cylinders with a single, photocrosslinked network corresponding to different SF concentrations and printing times. The printing parameters were 2.5-10% SF, 0.25-mM Ru/2.5-mM SPS, and 3 mW cm^{-2} of light intensity. SF: silk fibroin.

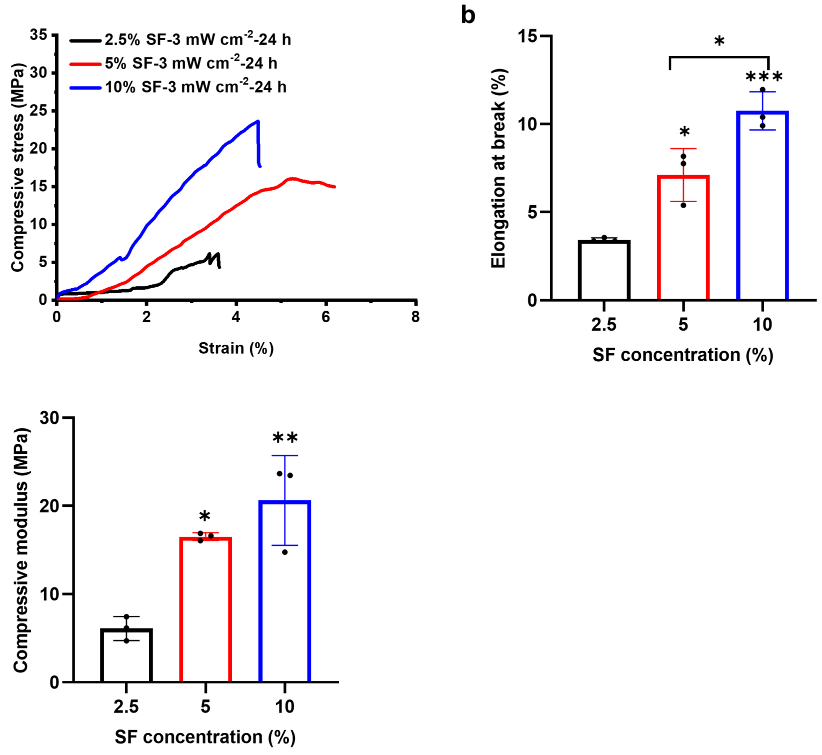

Supplementary Fig. 25.

Compressive strengths of volumetrically printed and double-crosslinked SF cylinders. (**a**) Compressive stress-strain curves of volumetrically printed and double-crosslinked silk cylinders, where the double-crosslinked network was induced by immersion in 70% ethanol solution for 24 h followed by 72 h of air-drying. (**b**) Quantified elongation at break comparison of the corresponding SF cylinders. (**c**) Quantified compressive stress at break comparison of the corresponding SF cylinders. The printing parameters were 2.5-10% SF, 0.25-mM Ru/2.5-mM SPS, and 3 mW cm^{-2} of light intensity. SF: silk fibroin. Statistical significances are expressed as *p<0.05, **p<0.01, ***p<0.001, compared to the corresponding 2.5% SF groups. One-way ANOVA. Data are presented as mean values ± SDs. n=3 independent experiments.

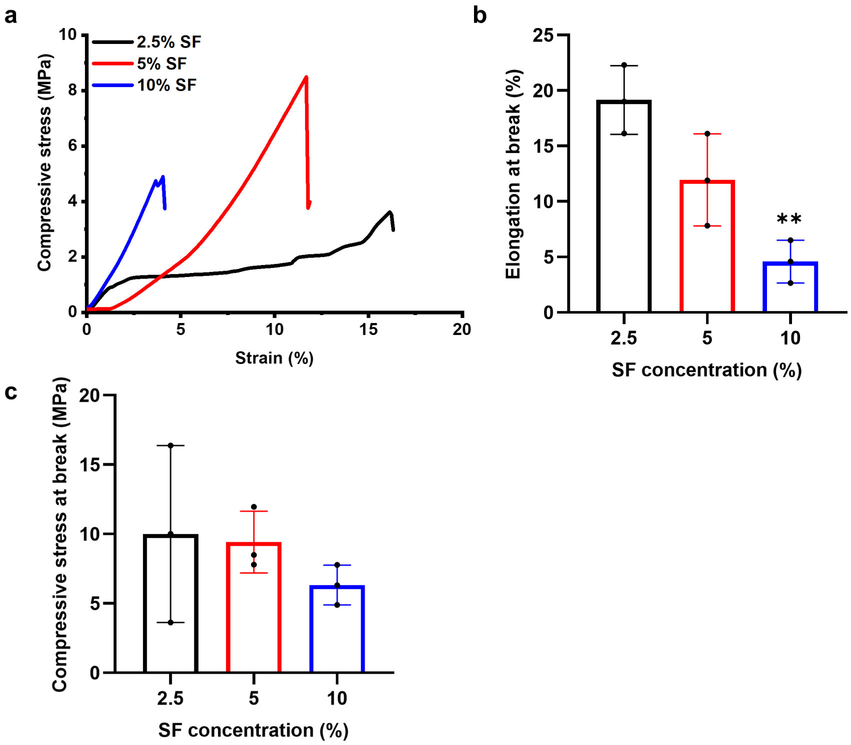

Supplementary Fig. 26.

Compressive strength of SF cylinders with a single, physically crosslinked *β*-sheet network induced by immersion in 70% ethanol solution for 24 h followed by 72 h of air-drying. (**a**) Compressive stress-strain curves of the 2.5-10% SF cylinders. (**b**) Quantified elongations at break of the corresponding SF cylinders. (**c**) Quantified compressive stresses at break of the corresponding SF cylinders. SF: silk fibroin. Statistical significance is expressed as $^{**}p<0.01$, compared to the corresponding 2.5% SF groups. One-way ANOVA. Data are presented as mean values ± SDs. $n=3$ independent experiments.

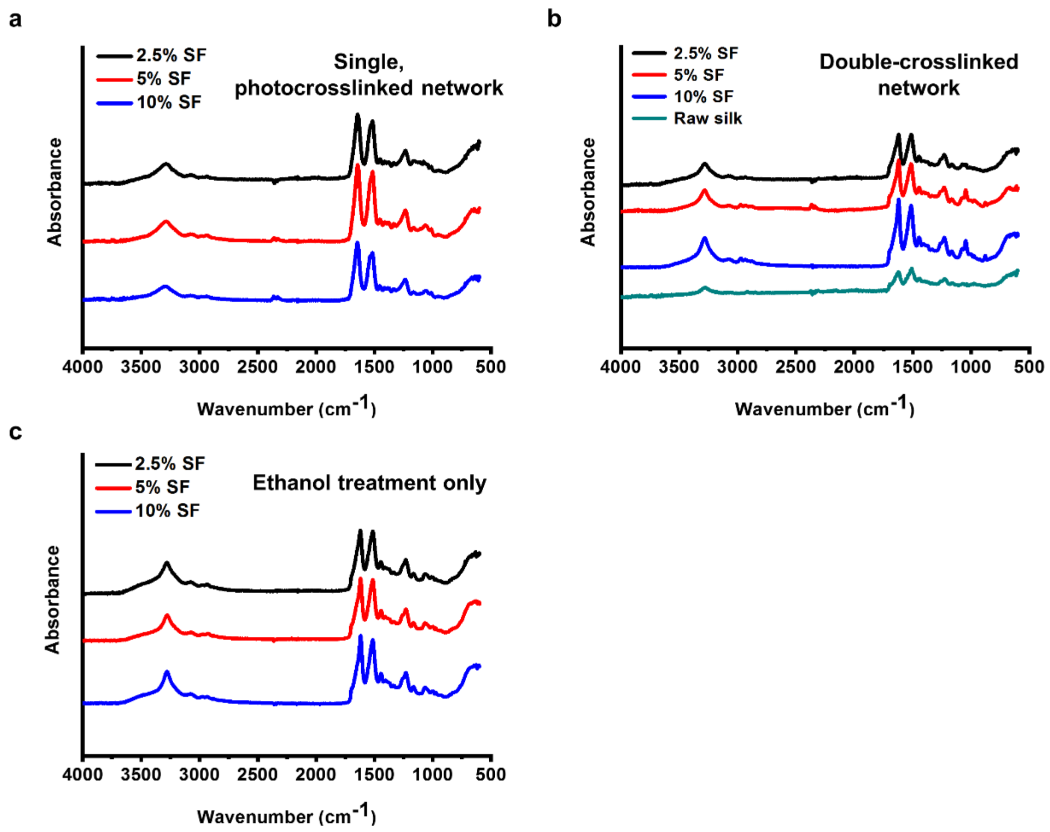

Supplementary Fig. 27.

FTIR spectra of silk objects with different SF concentrations and crosslinking configurations. (**a**) Volumetrically manufactured SF objects with a single, photocrosslinked network. (**b**) Raw silk fibers, and volumetrically manufactured SF objects with a double-crosslinked network induced by immersion in 70% ethanol solution for 24 h followed by 72 h of air-drying. The printing parameters for (**a**) and (**b**) were 2.5-10% SF, 0.25-mM Ru/2.5-mM SPS, and 3 mW cm^{-2} of light intensity. (**c**) SF objects with a single, physically crosslinked *β*-sheet network induced by immersion in 70% ethanol solution for 24 h followed by 72 h of air-drying. SF: silk fibroin.

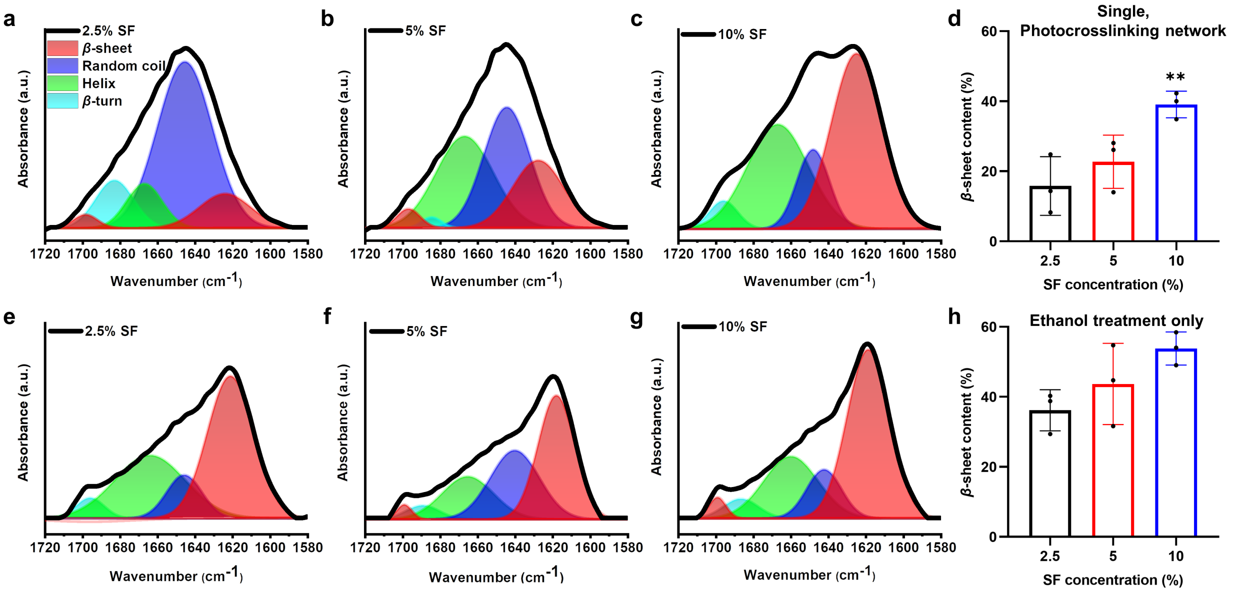

Supplementary Fig. 28.

FTIR spectra and *β*-sheet content comparisons of silk objects with different SF concentrations and crosslinking configurations. (**a-d**) Spectra of volumetrically printed 2.5-10% SF objects with a single, photocrosslinked network. The printing parameters were 0.25-mM Ru/2.5-mM SPS, and 3 mW cm^{-2} of light intensity. (**e-h**) Spectra of 2.5-10% SF objects with a single, physically crosslinked *β*-sheet network induced by immersion in 70% ethanol solution for 24 h followed by 72 h of air-drying. SF: silk fibroin. Statistical significance is expressed as $^{**}p<0.01$, compared to the corresponding 2.5% SF groups. One-way ANOVA. Data are presented as mean values ± SDs. n=3 independent experiments.

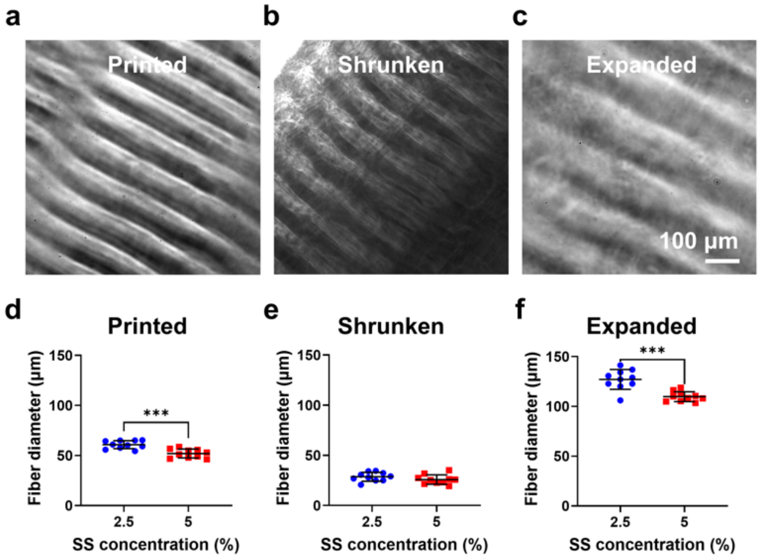

Supplementary Fig. 29.

Resolution changes of volumetrically printed SS objects during shrinking and expanding process. (**a-c**) SEM images of volumetrically printed tubular mesh structure (5% SS, 0.5-mM Ru/5-mM SPS, 3 mW cm^{-2} of light intensity, and 95 s of printing time.) in the printed, shrunken (induced by immersion in 100% ethanol for 2 h), and re-expanded (induced by immersion in water for 2 h) states. (**d-f**) Fiber diameter profiles of the volumetrically printed tubular mesh structures with different SS concentrations in the as-printed, shrunken, and re-expanded states. SS: silk sericin. Statistical significances are expressed as (**d**) ***p=0.0002, (**f**) ***p=0.0001. Unpaired t-test. Data are presented as mean values ± SDs. n=10 independent experiments.

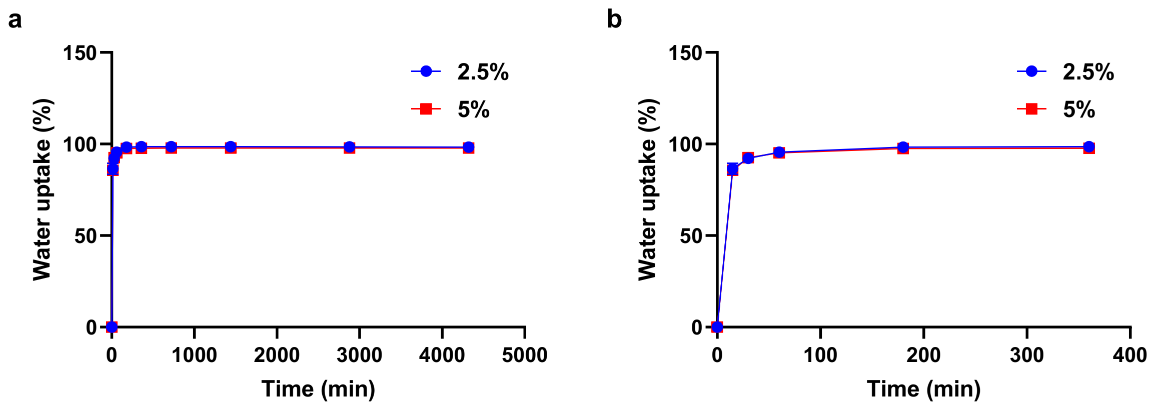

Supplementary Fig. 30.

Water-uptake profiles of the volumetrically printed SS cylinders in the shrunken state (induced with immersion in 100% ethanol for 24 h followed by 72 h of air-drying). (**a**) Over 72 h and (**b**) over 6 h. Printing parameters: 2.5% and 5% SS with 0.5-mM Ru/5-mM SPS, 3 mW cm^{-2} of light intensity, printing time: 28 s.

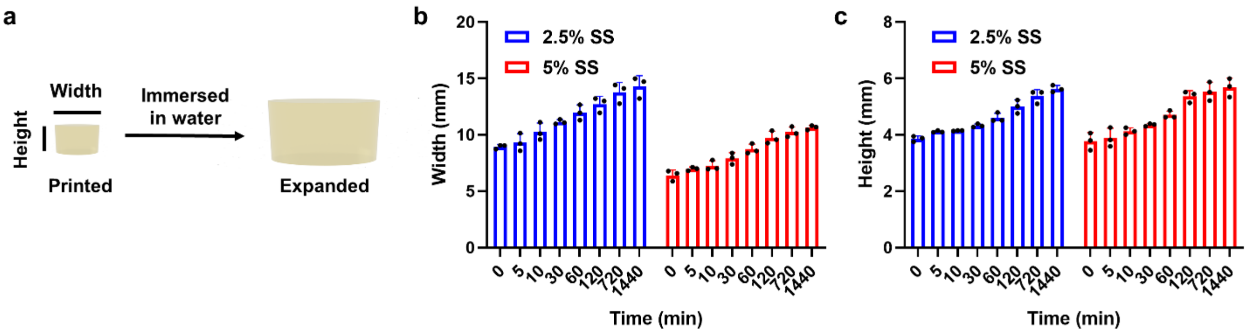

Supplementary Fig. 31.

Dimensional changes of the printed SS cylinders after immersion in water for 3 days. (**a**) Illustration of dimensional changes of the printed SS cylinders after immersion in water. (**b**) Width changes. (**c**) Height changes. Printing parameters: 2.5% and 5% SS with 0.5-mM Ru/5-mM SPS, 3 mW cm^{-2} of light intensity, printing time: 28 s. SS: silk sericin. Data are presented as mean values ± SDs. *n*=3 independent experiments.

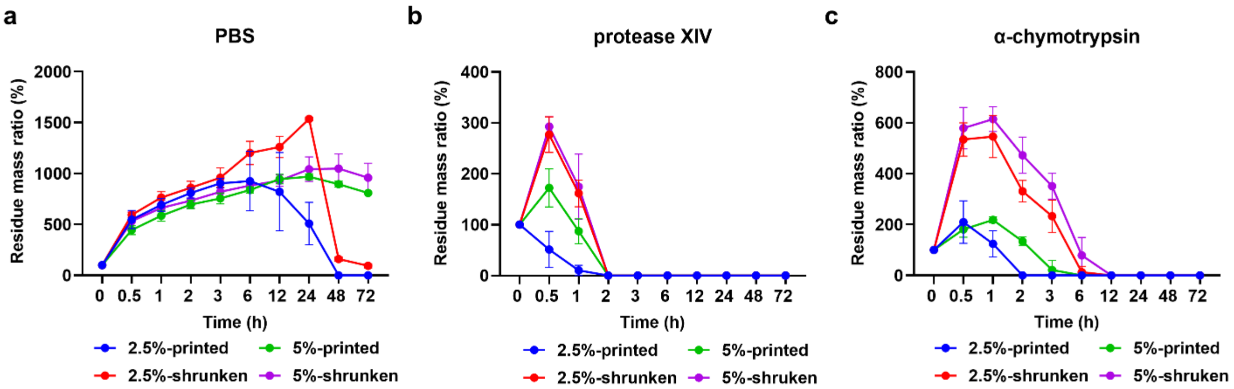

Supplementary Fig. 32.

***In vitro* degradation profiles of SS prints.** *In vitro* degradation profile of the volumetrically printed SS constructs in the as-printed and shrunken states (induced with immersion in 100% ethanol for 24 h followed by 72 h of air-drying) in (**a**) PBS solution, (**b**) 5 U mL^{-1} of protease XIV PBS solution, and (**c**) 40 U mL^{-1} of α-chymotrypsin PBS solution for up to 72 h. PBS: phosphate-buffered saline. Data are presented as mean values ± SDs. n=3 independent experiments.

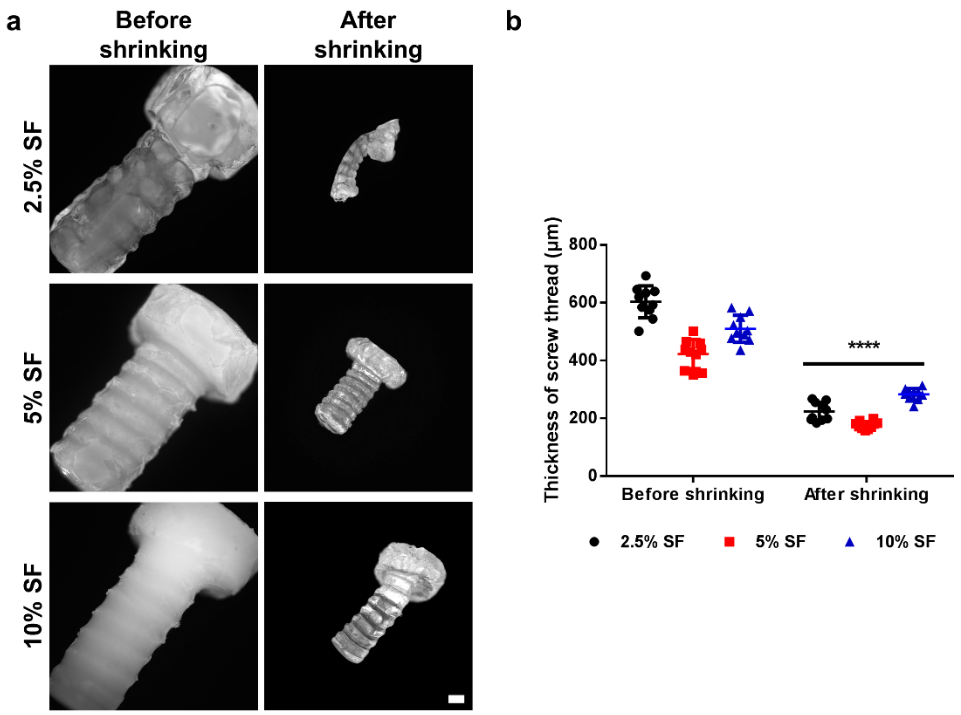

Supplementary Fig. 33.

Shape and resolution changes of volumetrically printed SF screws with a double-crosslinked network after 70% ethanol treatment for 24 h followed by 72 h of air-drying. (**a**) Microscopic images show volumetrically printed 2.5-10% SF screws before (*i.e.*, after 24 h-ethanol treatment but before air-drying) and after shrinking (*i.e.*, induced by 72 h of air-drying after 24 h-ethanol treatment). The printing parameters were 0.25-mM Ru/2.5-mM SPS, and 3 mW cm^{-2} of light intensity. Scale bar: 500 µm. (**b**) Quantified thread thickness changes of the same SF screws. SF: silk fibroin. Statistical significance is expressed as ****p<0.0001, compared to the corresponding groups before shrinking. One-way ANOVA. Data are presented as mean values ± SDs. n=10 independent experiments.

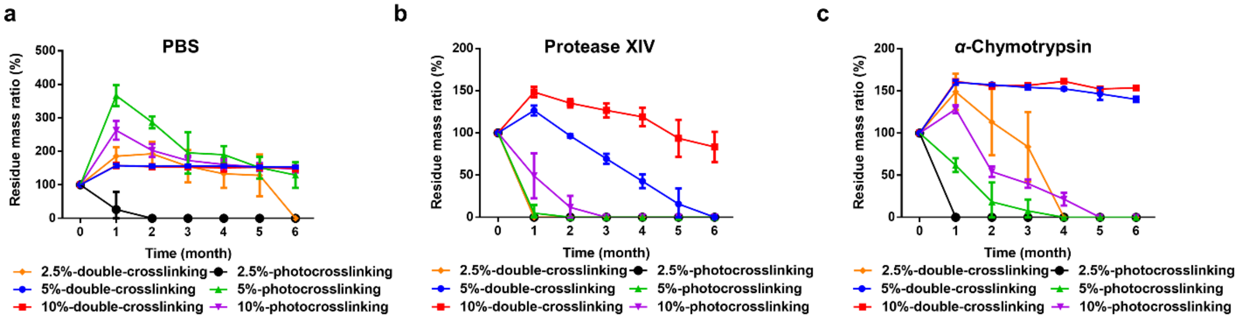

Supplementary Fig. 34.

***In vitro* degradation profiles of SF prints.** *In vitro* degradation profile of the volumetrically printed SF screws with a single, photocrosslinked network and a double-crosslinked network (treated with 70% ethanol for 24 h followed by 72 h of air-drying) in (**a**) PBS solution, (**b**) 5 U mL^{-1} of protease XIV PBS solution, and (**c**) 40 U mL^{-1} of α-chymotrypsin PBS solution for up to 6 months. PBS: phosphate-buffered saline. Data are presented as mean values ± SDs. n=3 independent experiments.

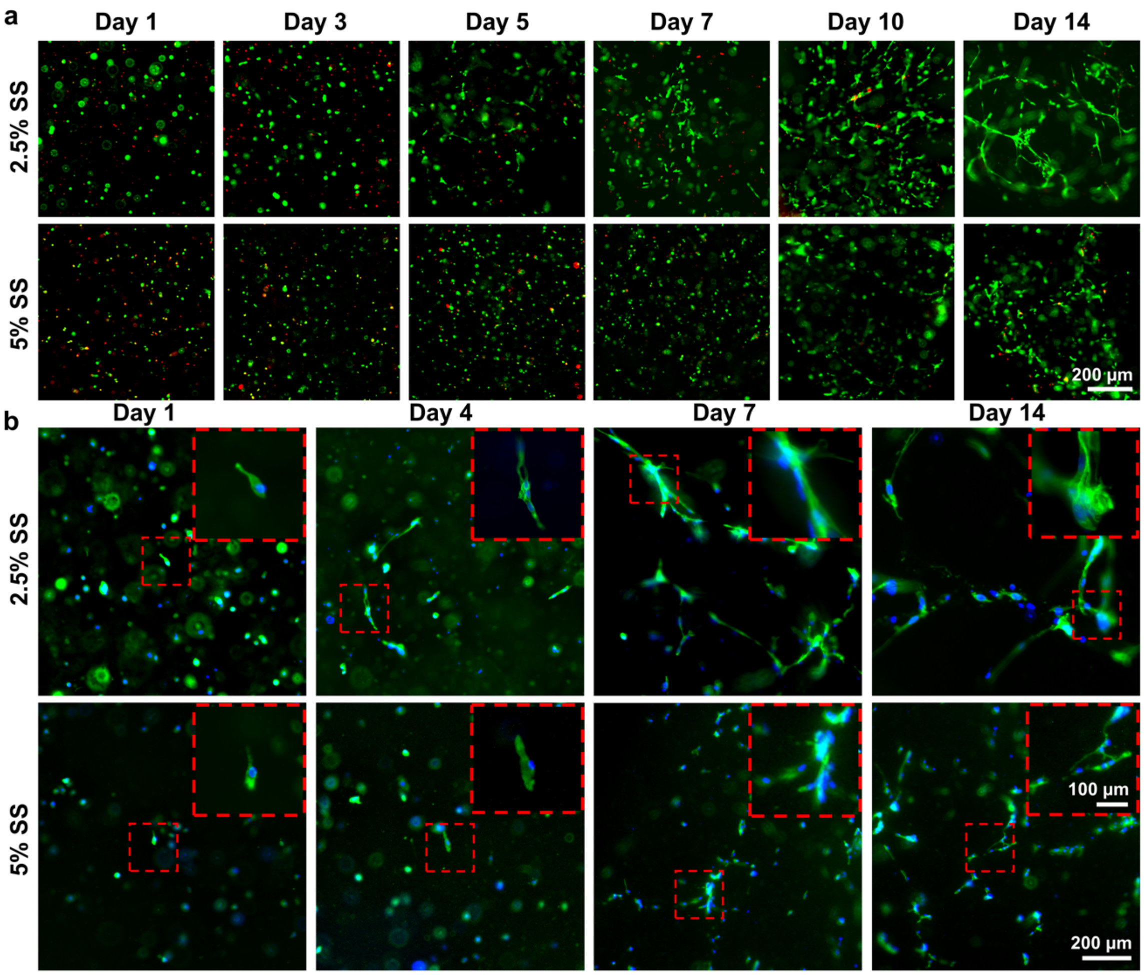

Supplementary Fig. 35.

Cytocompatibility of SS (bio)ink formulations with myoblasts (C2C12) cells. (**a**) Live/dead images (live cells in green and dead in red) of the corresponding cells in the volumetrically printed 2.5% and 5% SS hydrogels over 14 days of culture. Printing parameters: 5×10^6 cells mL^{-1}, 0.5-mM Ru/5-mM SPS, 3 mW cm^{-2} of light intensity, printing time: 28 s. (**b**) Microscopic images showing the growth of C2C12 cells stained for F-actin (green) and nuclei (blue) within the volumetrically printed 2.5% and 5% SS hydrogels. SS: silk sericin. n=3 independent experiments.

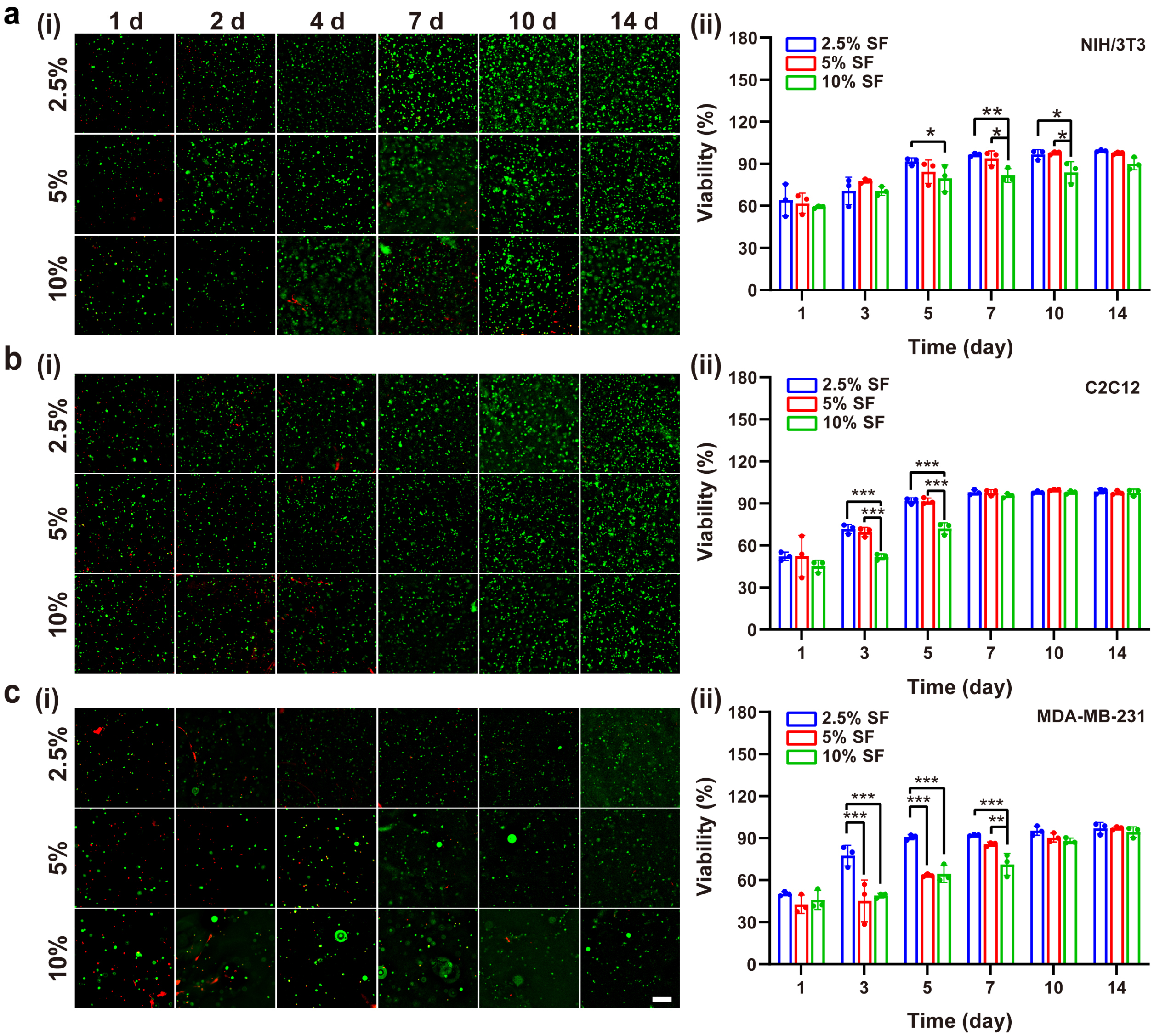

Supplementary Fig. 36.

Cytocompatibility of SF (bio)ink formulations with different cell types. (**a**) Fibroblasts (NIH/3T3), (**b**) myoblasts (C2C12), and (**c**) breast cancer cells (MDA-MB-231). (**i**) Live/dead images (live cells in green and dead in red) and (**ii**) quantified cell viability values of the corresponding cells in the volumetrically printed 2.5-10% SF hydrogels over 14 days of culture. The printing parameters were 0.25 mM-Ru/2.5-mM SPS, 5×10^6 cells mL^{-1}, and 3 mW cm^{-2} of light intensity. Scale bar: 200 µm. SF: silk fibroin. C2C12: Myoblasts. NIH/3T3: fibroblasts. MDA-MB-231: breast cancer cells. Statistical significances are expressed as (**a(ii)**) *adjusted p=0.0493 (5 day); *adjusted p=0.0391 (7-day), **p=0.01 (7-day); *adjusted p=0.0332 (10-day, 5% vs 10%), *adjusted p=0.0188 (10-day, 2.5% vs 10%). (**b(ii)**) ***adjusted p<0.0001. (**c(ii)**) **adjusted

=0.007, ***adjusted p=0.0001. Two-way ANOVA. Data are presented as mean values ± SDs. n=3 independent experiments.

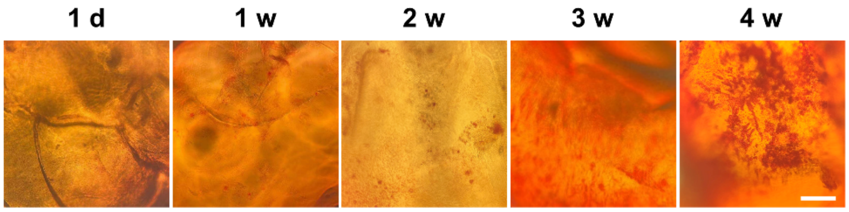

Supplementary Fig. 37.

ARS staining images showing deposition of calcium on the surface of volumetrically printed 10% SF screws with a double-crosslinked network (induced by immersed in 70% ethanol for 24 h) at 4 weeks of culture. Scale bar: 100 μm. *n*=3 independent experiments.

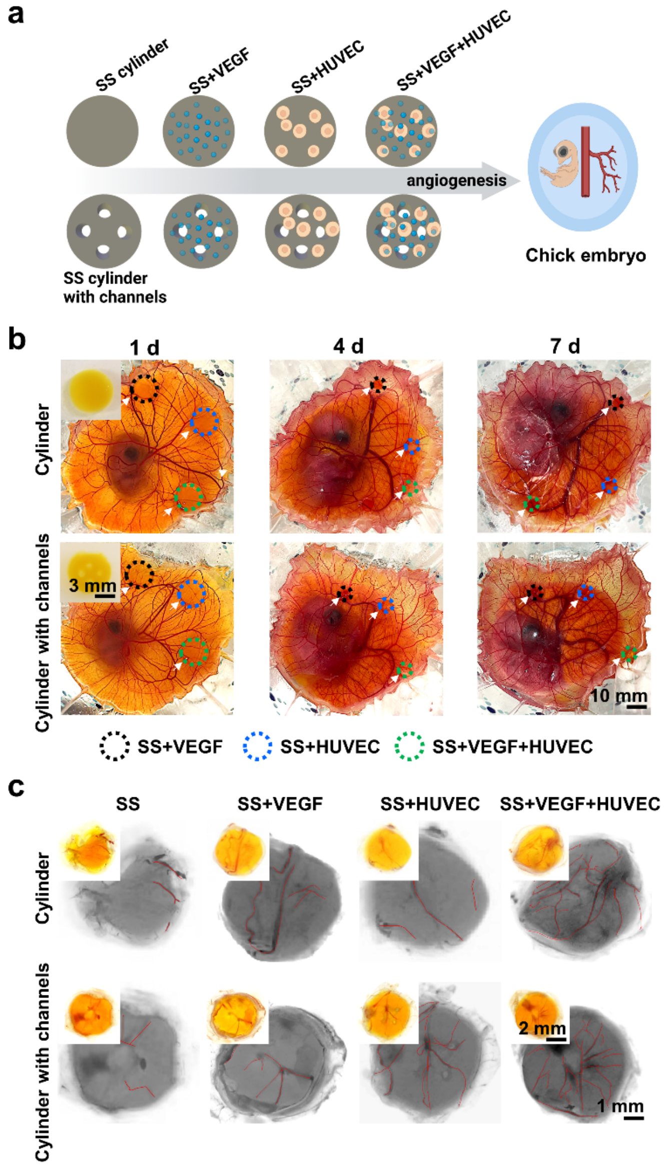

Supplementary Fig. 38.

***Ex ovo* chick CAM assay**. The printing parameters for the following tests were 2.5% SS, 0.5-mM Ru/5-mM SPS, 5×10^{6} cells mL^{-1}, and 3 mW cm^{-2} of light intensity. (**a**) Illustration of volumetrically bioprinted SS cylinders and cylinders with channels with HUVECs or/and VEGF embedded for *ex ovo* chick CAM assay. Created with BioRender.com. (**b**) Representative images of chick *ex ovo* culture with implanted volumetrically bioprinted constructs for CAM assay in different timesteps. White arrows indicated the implanted volumetrically bioprinted constructs. (**c**) Compatibility of the volumetrically bioprinted SS constructs after cultured for 7 days. SS: silk sericin. HUVEC: human umbilical vein endothelial cell. VEGF: vascular endothelial growth

factor.

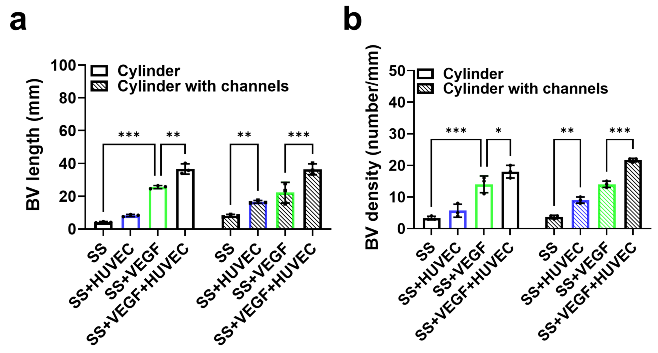

Supplementary Fig. 39.

Quantification of the BV growth surrounding and within the volumetrically bioprinted constructs. (**a**) Lengths of the BVs surrounding the constructs, (**b**) BV densities within the constructs. SS: silk sericin. HUVEC: human umbilical vein endothelial cell. VEGF: vascular endothelial growth factor. Statistical significances are expressed as (**a**) **adjusted p=0.001, ***adjusted p<0.001. (**b**) *adjusted p=0.024, **adjusted p=0.003, ***adjusted p<0.001. Two-way ANOVA. Data are presented as mean values ± SDs. n=3 independent experiments.

Supplementary Table 1.

Penetration depth, printability, and printing time of the SF (bio)ink formulations used. The printing parameters were 1:10 of Ru-SPS ratio, and 3 mW cm^{-2} of light intensity. Circles represent printable; triangles represent printable, but the shape was not agreement with CAD; crosses represent non-printable.

SF/Ru concentrations	Penetration depth (cm)	Printability
**20% SF**	2.43	
0.0625-mM Ru	1.97	×
0.125-mM Ru	0.78	×
0.25-mM Ru	0.62	×
0.5-mM Ru	0.50	×
1-mM Ru	0.35	×
1.5-mM Ru	0.25	×
2-mM Ru	0.20	×
**15% SF**	2.47	
0.0625-mM Ru	2.10	×
0.125-mM Ru	1.17	×
0.25-mM Ru	0.94	△
0.5-mM Ru	0.66	△
1-mM Ru	0.42	×
1.5-mM Ru	0.29	×
2-mM Ru	0.22	×
**10% SF**	2.77	
0.0625-mM Ru	2.29	×
0.125-mM Ru	1.32	×
0.25-mM Ru	1.12	○
0.5-mM Ru	0.69	△
1-mM Ru	0.43	×
1.5-mM Ru	0.30	×

2-mM Ru	0.29	×
**5% SF**	3.00	
0.0625-mM Ru	2.48	×
0.125-mM Ru	1.49	△
0.25-mM Ru	1.12	○
0.5-mM Ru	0.71	○
1-mM Ru	0.45	×
1.5-mM Ru	0.35	×
2-mM Ru	0.29	×
**2.5% SF**	3.01	
0.0625-mM Ru	2.55	×
0.125-mM Ru	2.00	△
0.25-mM Ru	1.53	○
0.5-mM Ru	0.96	△
1-mM Ru	0.60	△
1.5-mM Ru	0.42	×
2-mM Ru	0.30	×
**1.25% SF**	3.21	
0.0625-mM Ru	2.56	×
0.125-mM Ru	2.03	△
0.25-mM Ru	1.64	△
0.5-mM Ru	0.96	△
1-mM Ru	0.63	×
1.5-mM Ru	0.44	×
2-mM Ru	0.34	×
**0.625% SF**	3.40	
0.0625-mM Ru	2.56	×

0.125-mM Ru	2.05	×
0.25-mM Ru	1.69	×
0.5-mM Ru	1.02	×
1-mM Ru	0.66	×
1.5-mM Ru	0.48	×
2-mM Ru	0.40	×

Supplementary Table 2.

Full-factorial design for exploring the three SF factors on volumetric printing resolution.

Factor	Levels	
SF concentration (%)	2.5	5
SF Mw (kDa)	88.9	108.7
Ru concentration (mM)	0.25	0.5

Supplementary Table 3.

Wavenumbers and vibrational assignments of the volumetrically printed SS constructs.

Wavenumber (cm^{-1})	Approximate assignment of vibrational mode
1,658-1,666	(CO) amide I
1,616	(C=C) olefinic
1,605	(C=C) aromatic ring
1,449	(CH_2) scissoring
1,404	$C(CH_3)_2$ asymmetric
1,334	CH_3
1,313	CH_2
1,260	(CN) amide III disordered
1,231	CN; (CH_2) disordered
1,172	CC
1,161	CC; COH
1,085	(CC) skeletal; random configuration
1,046	(CC) skeletal
1,034	(CC) skeletal
1,003	(CC) aromatic ring
997	CH_3
946	CH_3
880	CH_2
854	(CCH) aromatic; tyrosine
829	Tyrosine

Supplementary Table 4.

Primer sequences of osteogenesis-related genes.

Gene	Primer sequence (5‘-3’)	Annealing temperature (°C)	Product size (bp)	Cycle	$MgCl_2$
**Reference gene**					
GAPDH	F: GCCAAAAGGGTCATCATCTC<br>R: GGCCATCCACAGTCTTCT	58	20	40	1×
**Osteogenic marker genes**					
Runx	F: CAACCCACGAATGCACTATCC<br>R: TGATAGGATCCTGACGAAGTG	58	21	40	1×
ALP	F: CCTCCTCGGAAGACACTCTGA<br>R: TGATAGGATCCTGACGAAGTG	58	21	40	1×
Col1α1	F: AGGGCCAAGACGAAGACATCC<br>R: TTCTTGGTCGGTGGGTGACTC	58	21	40	1×
BMP2	F: GTGACGTGGGGTGGAATGACT<br>R: ACAGCATCGAGATAGCACTGA	58	21	40	1×
OPN	F: ATAGTGTGGTTTATGGACTGA<br>R: CGTTTCATAACTGTCCTTCCC	58	21	40	1×
OCN	F: CAACCCACGAATGCACTATCC<br>R: TGCCTGGAGAGGAGCAGAACT	58	21	40	1×
Osx	F: TGCTTGAGGAGGAAGTTCACT<br>R: AGTGCTTGTAAAGGGGGCTGG	58	21	40	1×

bp: base pair; F: forward primer; R: reverse primer; *GAPDH*: glyceraldehyde-3-phosphate dehydrogenase; *Runx*: runt-related transcription factor; *ALP*: alkaline phosphatase; *COL1α1*: collagen type I alpha 1 chain; *BMP2*: bone morphogenetic protein 2; *OPN*: osteopontin; *OCN*: osteocalcin; *Osx*: osterix

Supplementary Catalog Numbers for Commercial Reagents

Material	Vendor	Catalog #
4',6-diamidino-2-phenylindole (DAPI)	MilliporeSigma	D9542-10MG
α-chymotrypsin	MilliporeSigma	c4129
Alexa 488-phalloidin	Thermo Fisher Scientific	A12379
antibiotic-antimycotic (Anti-Anti)	Thermo Fisher Scientific	15240-062
anti-RUNX2 antibody	Abcam	ab76956
Bis-Tris- gel (NuPAGE 4-12%)	Invitrogen	NP0326BOX
breast cancer cell (MDA-MB-231)	ATCC	HTB-26
dialysis membrane	Thermo Fisher Scientific	08-700-166
DMSO	MilliporeSigma	CC-3162
Dulbecco's modified Eagle medium (DMEM)	Thermo Fisher Scientific	11965-118
Dulbecco's PBS (DPBS)	Thermo Fisher Scientific	14190-250
ethanol	MilliporeSigma	V1101
fetal bovine serum (FBS)	Thermo Fisher Scientific	10437-028
fibroblast (NIH/3T3)	ATCC	CRL-1658
fluorescent color dyes	CreateX Colors	5801-00
formalin	MilliporeSigma	MKCG9856
goat anti-mouse IgG H&L (Alexa Fluor 488)	Abcam	ab150113
hMSC differentiation medium	Lonza	PT-3002
HUVEC	ATCC	CRL-1730
lithium bromide (LiBr)	MilliporeSigma	213225-100G
Live/dead viability/cytotoxicity kit	Thermo Fisher Scientific	L3224
mouse anti-osteocalcin antibody	Abcam	ab198228
MTS assy kit	Thermo Fisher Scientific	PRG-3581
myoblast (C2C12)	ATCC	CRL-1772
PrestoBlue	Thermo Fisher Scientific	A13261
protease XIV	MilliporeSigma	p5147
RNeasy mini kit and QuantiTect reverse transcription kit	Qiagen	205311
Ru / SPS	Advanced Biomatrix, USA	5248
sodium carbonate Na2CO3	MilliporeSigma	13418-1KG-R
Sterile syringe filters	VWR International	S2GPU05RE
SYBR™ Green qPCR Master Mix	Thermo Fisher Scientific	A25742
Triton X-100	MilliporeSigma	1003287133
trypsin-ethylenediaminetetraacetic acid (EDTA)	Thermo Fisher Scientific	15400-054
vacuum filtration systems	VWR International	CLS430186-1EA
