# Supplementary material for: Volumetric additive manufacturing of pristine silk-based (bio)inks
Source: Nat Commun. 2023 Jan 13;14:210. doi: 10.1038/s41467-023-35807-7 (PMC9839706; doi:10.1038/s41467-023-35807-7)
Supplement: Supplementary file 2 — Description of Additional Supplementary Files [file 41467_2023_35807_MOESM2_ESM.pdf]

Description of Additional Supplementary Files

File Name: Supplementary Movie 1
Description: VAM of a-channel-in-a-cube structure with the printing parameters: 2.5% SS, 0.5-mM Ru/5-mM SPS, and 3 mW cm^{-2} of light intensity.

File Name: Supplementary Movie 2
Description: VAM of a hollow triangle structure with the printing parameters: 2.5% SS, 0.5-mM Ru/5-mM SPS, and 3 mW cm^{-2} of light intensity.

File Name: Supplementary Movie 3
Description: VAM of a diamond ring structure with the printing parameters: 2.5% SS, 0.5-mM Ru/5-mM SPS, and 3 mW cm^{-2} of light intensity.

File Name: Supplementary Movie 4
Description: VAM of a C60-like structure with the printing parameters: 2.5% SS, 0.5-mM Ru/5-mM SPS, and 3 mW cm^{-2} of light intensity

File Name: Supplementary Movie 5
Description: VAM of a brain structure with the printing parameters: 2.5% SS, 0.5-mM Ru/5-mM SPS, and 3 mW cm^{-2} of light intensity.

File Name: Supplementary Movie 6
Description: VAM of an ear structure with the printing parameters: 2.5% SS, 0.5-mM Ru/5-mM SPS, and 3 mW cm^{-2} of light intensity.

File Name: Supplementary Movie 7
Description: VAM of a lobster structure with the printing parameters: 2.5% SF, 0.25-mM Ru/2.5-mM SPS, and 3 mW cm^{-2} of light intensity.

File Name: Supplementary Movie 8
Description: VAM of a heart structure with the printing parameters: 2.5% SF, 0.25-mM Ru/2.5-mM SPS, and 3 mW cm^{-2} of light intensity.

File Name: Supplementary Movie 9
Description: VAM of a hollow square structure with the printing parameters: 2.5% SS, 0.5-mM Ru/5-mM SPS, and 3 mW cm^{-2} of light intensity.

File Name: Supplementary Movie 10
Description: VAM of a terracotta warrior structure with the printing parameters: 2.5% SF, 0.25-mM Ru/2.5- mM SPS, and 3 mW cm^{-2} of light intensity.

File Name: Supplementary Movie 11
Description: VAM of a screw structure with the printing parameters: 2.5% SF, 0.25-mM Ru/2.5-mM SPS, and 3 mW cm^{-2} of light intensity.
