# Supplementary material for: Volumetric additive manufacturing of pristine silk-based (bio)inks
Source: Nat Commun. 2023 Jan 13;14:210. doi: 10.1038/s41467-023-35807-7 (PMC9839706; doi:10.1038/s41467-023-35807-7)
Supplement: Supplementary file 14 — Reporting Summary [file 41467_2023_35807_MOESM14_ESM.pdf]

nature portfolio

Corresponding author(s): Yu Shrike Zhang

Last updated by author(s): Nov 28, 2022

Reporting Summary

Nature Portfolio wishes to improve the reproducibility of the work that we publish. This form provides structure for consistency and transparency in reporting. For further information on Nature Portfolio policies, see our Editorial Policies and the Editorial Policy Checklist.

Statistics

For all statistical analyses, confirm that the following items are present in the figure legend, table legend, main text, or Methods section.

n/a	Confirmed	
☐	☒	The exact sample size (*n*) for each experimental group/condition, given as a discrete number and unit of measurement
☐	☒	A statement on whether measurements were taken from distinct samples or whether the same sample was measured repeatedly
☐	☒	The statistical test(s) used AND whether they are one- or two-sided<br>*Only common tests should be described solely by name; describe more complex techniques in the Methods section.*
☒	☐	A description of all covariates tested
☒	☐	A description of any assumptions or corrections, such as tests of normality and adjustment for multiple comparisons
☐	☒	A full description of the statistical parameters including central tendency (e.g. means) or other basic estimates (e.g. regression coefficient) AND variation (e.g. standard deviation) or associated estimates of uncertainty (e.g. confidence intervals)
☐	☒	For null hypothesis testing, the test statistic (e.g. *F*, *t*, *r*) with confidence intervals, effect sizes, degrees of freedom and *P* value noted<br>*Give P values as exact values whenever suitable.*
☒	☐	For Bayesian analysis, information on the choice of priors and Markov chain Monte Carlo settings
☒	☐	For hierarchical and complex designs, identification of the appropriate level for tests and full reporting of outcomes
☒	☐	Estimates of effect sizes (e.g. Cohen's *d*, Pearson's *r*), indicating how they were calculated

Our web collection on statistics for biologists contains articles on many of the points above.

Software and code

Policy information about availability of computer code

Data collection	The main software used includes Image J 1.53K
Data analysis	The main software used includes Image J, Origin8, Graphpad9

For manuscripts utilizing custom algorithms or software that are central to the research but not yet described in published literature, software must be made available to editors and reviewers. We strongly encourage code deposition in a community repository (e.g. GitHub). See the Nature Portfolio guidelines for submitting code & software for further information.

Data

Policy information about availability of data

All manuscripts must include a data availability statement. This statement should provide the following information, where applicable:

- Accession codes, unique identifiers, or web links for publicly available datasets
- A description of any restrictions on data availability
- For clinical datasets or third party data, please ensure that the statement adheres to our policy

The raw RNA-Seq data generated in this study have been deposited in the National Center for Biotechnology Information (NCBI) Sequence Read Archive (SRA) as a BioProject under the Accession Number “PRJNA836719”. All other relevant data supporting the key findings of this study are available within the article and its Supplementary Information files as well as Source Data. Requests for additional raw images and materials will be promptly reviewed by the Brigham and Women’s Hospital, and will be released via a Material Transfer Agreement. Source data are provided with this paper.

Human research participants

Policy information about studies involving human research participants and Sex and Gender in Research.

Reporting on sex and gender	n/a
Population characteristics	n/a
Recruitment	n/a
Ethics oversight	n/a

Note that full information on the approval of the study protocol must also be provided in the manuscript.

Field-specific reporting

Please select the one below that is the best fit for your research. If you are not sure, read the appropriate sections before making your selection.

☒ Life sciences ☐ Behavioural & social sciences ☐ Ecological, evolutionary & environmental sciences

For a reference copy of the document with all sections, see nature.com/documents/nr-reporting-summary-flat.pdf

Life sciences study design

All studies must disclose on these points even when the disclosure is negative.

Sample size	Sample size was at least n=3 independent experiments throughout the whole experiments. It allowed for adequate analysis to reach meaningful conclusions of the data. One-way analysis of variance (ANOVA) or two-way ANOVA with Tukey's post hoc multiple comparison test or unpaired t test were used to determine statistical significance.
Data exclusions	There was no sample excluded.
Replication	All attempts of replication were from at least 3 independent experiments. All experiments were repeatable and could be reproduced.
Randomization	Samples were collected randomly throughout the whole experiments.
Blinding	Blinding was not used in all experiments. The bias would not be changed, and all data were quantifiable.

Reporting for specific materials, systems and methods

We require information from authors about some types of materials, experimental systems and methods used in many studies. Here, indicate whether each material, system or method listed is relevant to your study. If you are not sure if a list item applies to your research, read the appropriate section before selecting a response.

Materials & experimental systems

n/a	Involved in the study	
☐	☒	Antibodies
☐	☒	Eukaryotic cell lines
☒	☐	Palaeontology and archaeology
☒	☐	Animals and other organisms
☒	☐	Clinical data
☒	☐	Dual use research of concern

Methods

n/a	Involved in the study	
☒	☐	ChIP-seq
☒	☐	Flow cytometry
☒	☐	MRI-based neuroimaging

Antibodies

Antibodies used	anti-RUNX2 antibody, Catalog number: ab76956, monoclonal (2B9);<br>anti-osteocalcin antibody, Catalog number: ab198228, polyclonal;<br>goat anti-mouse IgG H&L (Alexa Fluor 488), catalog number: ab150113, monoclonal.
Validation	anti-RUNX2:https://www.citeab.com/antibodies/760248-ab76956-anti-runx2-antibody-2b9

Validation	anti-osteocalcin: https://www.abcam.com/osteocalcin-antibody-ab198228.html<br>goat anti-mouse IgG H&L: https://www.abcam.com/goat-mouse-igg-hl-alexa-fluor-488-ab150113.html

Eukaryotic cell lines

Policy information about cell lines and Sex and Gender in Research

Cell line source(s)	C2C12, Catalog number: CRL-1772, this is a subclone (produced by H. Blau, et al) of the mouse myoblast cell line established by D. Yaffe and O. Saxel.<br>NIH/3T3, Catalog number: CRL-1658, NIH/3T3 is a fibroblast cell line that was isolated from a mouse NIH/Swiss embryo.<br>MDA-MB-231, Catalog number: CRM-HTB-26, this cell line is aneuploid female (modal number = 64, range = 52 to 68), with chromosome counts in the near-triploid range. Normal chromosomes N8 and N15 were absent.<br>HUVEC, Catalog number: CC-2517. ATCC primary pooled HUVECs were derived from 10 individual donors, minimizing the lot-to-lot variability associated with cells derived from single donors.
Authentication	All the cells were purchased from ATCC with authentication. C2C12: https://www.atcc.org/products/crl-1772. NIH/3T3: https://www.atcc.org/products/crl-1658. MDA-MB-231: https://www.atcc.org/products/crm-htb-26. Huvec: https://www.atcc.org/products/pcs-100-013
Mycoplasma contamination	All cells test negative for mycoplasma.
Commonly misidentified lines (See ICLAC register)	None
