# Supplementary figures and images for: Volumetric additive manufacturing of pristine silk-based (bio)inks
Source: Nat Commun. 2023 Jan 13;14:210. doi: 10.1038/s41467-023-35807-7 (PMC9839706; doi:10.1038/s41467-023-35807-7)

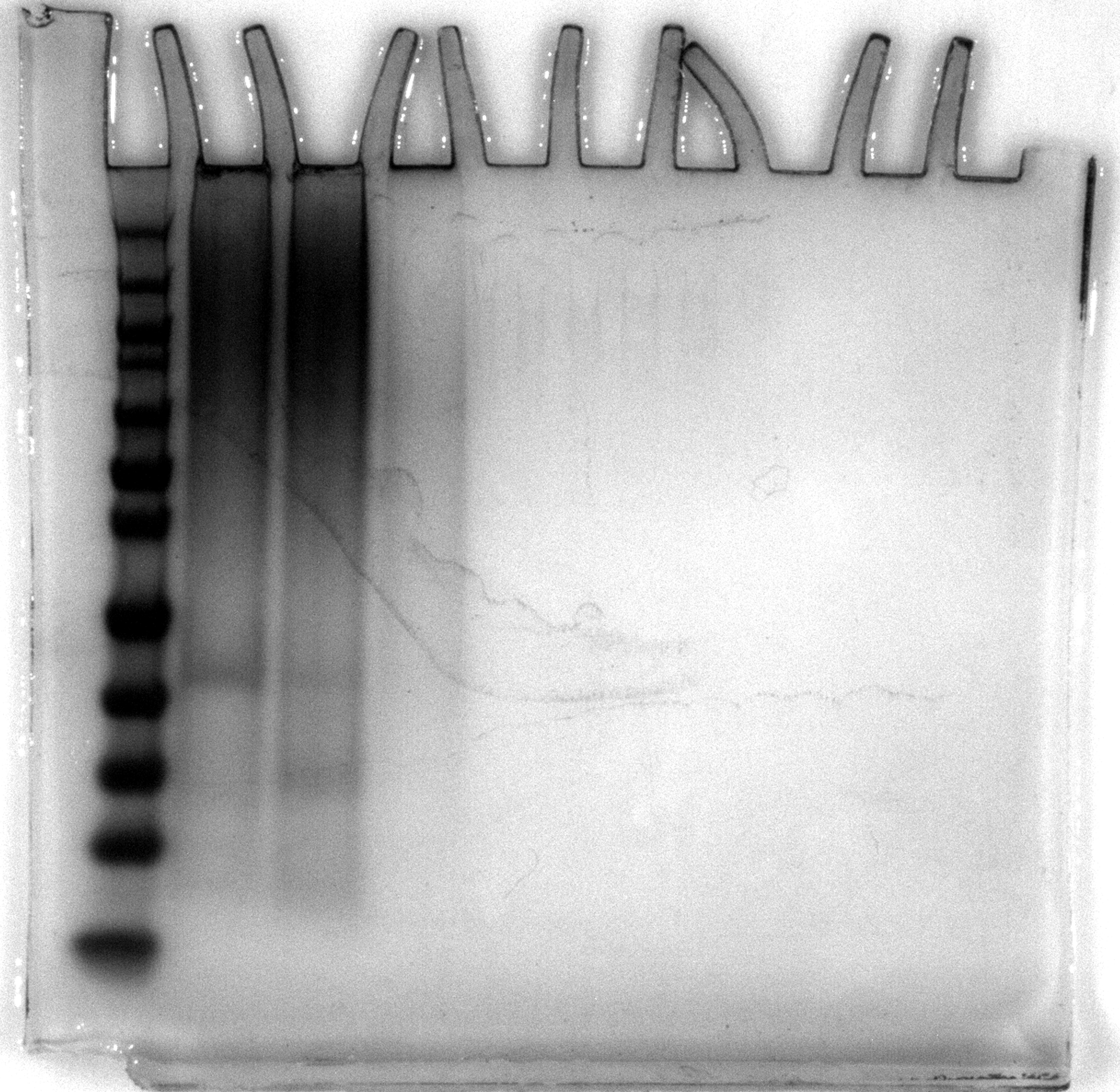

Supplement: Supplementary file 15 — Source Data [file 41467_2023_35807_MOESM15_ESM.zip › Source data/Supplementrary fig. 10a SDS PAGE Image uncropped_.tif]
